# Single-Cell Chemistry of Photoactivatable Platinum Anticancer Complexes

**DOI:** 10.1021/jacs.1c08630

**Published:** 2021-11-22

**Authors:** Elizabeth M. Bolitho, Carlos Sanchez-Cano, Huayun Shi, Paul D. Quinn, Maria Harkiolaki, Cinzia Imberti, Peter J. Sadler

**Affiliations:** † Department of Chemistry, University of Warwick, Gibbet Hill Road, Coventry CV4 7AL, United Kingdom; ‡ Diamond Light Source, Harwell Science and Innovation Campus, Fermi Avenue, Didcot OX11 0DE, United Kingdom; § Center for Cooperative Research in Biomaterials (CIC biomaGUNE), Basque Research and Technology Alliance (BRTA), Paseo de Miramon 182, 20014 San Sebastián, Spain

## Abstract

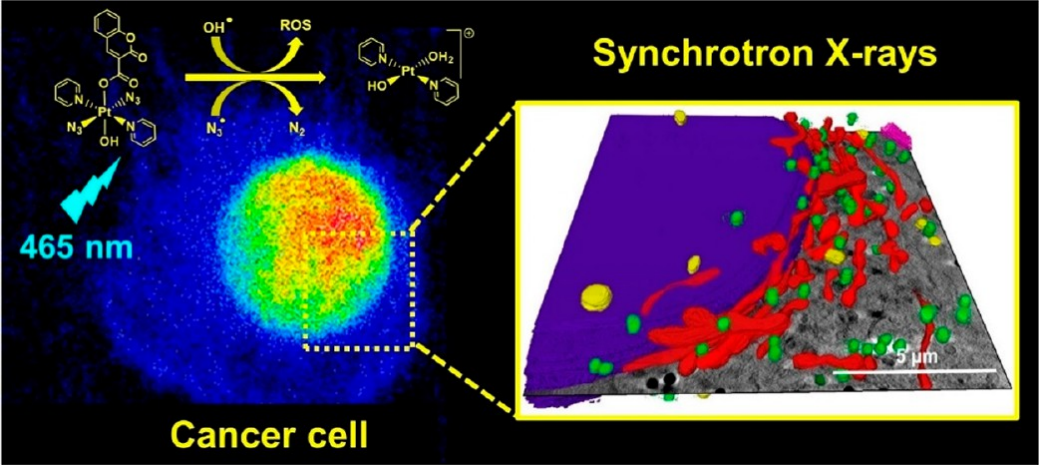

The Pt(IV) prodrug *trans, trans, trans*-[Pt(pyridine)_2_(N_3_)_2_(OH)_2_] (**Pt1**) and its coumarin derivative *trans, trans, trans*-[Pt(pyridine)_2_(N_3_)_2_(OH)(coumarin-3-carboxylate)] (**Pt2**) are promising agents for photoactivated chemotherapy. These complexes are inert in the dark but release Pt(II) species and radicals upon visible light irradiation, resulting in photocytotoxicity toward cancer cells. Here, we have used synchrotron techniques to investigate the in-cell behavior of these prodrugs and visualize, for the first time, changes in cellular morphology and Pt localization upon treatment with and without light irradiation. We show that photoactivation of **Pt2** induces remarkable cellular damage with extreme alterations to multiple cellular components, including formation of vacuoles, while also significantly increasing the cellular accumulation of Pt species compared to dark conditions. X-ray absorption near-edge structure **(**XANES) measurements in cells treated with **Pt2** indicate only partial reduction of the prodrug upon irradiation, highlighting that phototoxicity in cancer cells may involve not only Pt(II) photoproducts but also photoexcited Pt(IV) species.

## Introduction

The use of light to activate otherwise inert molecules selectively and generate a localized antiproliferative effect is a concept that has been used in cancer treatment for several decades in the form of photodynamic therapy (PDT). PDT has been successfully translated into clinical practice for the treatment of several types of accessible cancers, including skin, head and neck, prostate, and bladder cancer.^1^ Metal-based photosensitizers are now the focus of increasing interest in this rapidly expanding field.^1−6^

One major issue with PDT agents is their dependence on oxygen for antiproliferative activity, through the conversion of ground state ^3^O_2_ to excited state ^1^O_2_, although some recent metal-based photosensitizers have seemingly overcome this problem.^3,7,8^ A different type of light-activated therapy, whose mechanism of action does not require oxygen, is photoactivated chemotherapy (PACT), where light is used to modify chemically the structure of a prodrug, thus releasing active agents intracellularly. Light-activation mechanisms for metal-based PACT agents can be tuned through the choice of the metal and its ligand set to affect different photo(bio)chemical pathways through ligand exchange, photodissociation, and photoredox processes.^2^ Although currently the development of PACT therapies is not as advanced as PDT, there is substantial current preclinical research in the field.^9−11^

**Figure 1:**
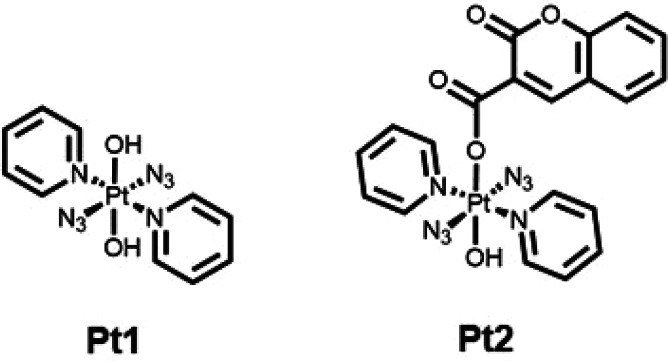
Structures of photoactivatable *trans* dihydroxido Pt(IV) complex **Pt1** and coumarin derivative **Pt2**.^16^

We have reported photoactivatable diazido Pt(IV) complexes that are inert in the dark but release cytotoxic Pt(II) species and azidyl radicals upon irradiation with visible light. The complex *trans,trans,trans*-[Pt(pyridine)_2_(N_3_)_2_(OH)_2_] (**Pt1**, Figure 1), for example, has potent photocytotoxic activity with micromolar half-maximal inhibitory concentrations (IC_50_) in several cancer cell lines using a short clinically-relevant treatment protocol (1 h incubation and 1 h irradiation with blue light), conditions under which cisplatin does not display antiproliferative activity (IC_50_ > 100 μM).^12^ While DNA is a target for this *trans*-diamine complex, the type of DNA-Pt lesions formed by photoactivated **Pt1** (mainly bifunctional interstrand cross-links) is markedly different from those formed by cisplatin.^13^ In addition to DNA damage, platinum binding to proteins and reactions mediated by reactive oxygen species (ROS) generated in the photoreduction process are observed for **Pt1**, highlighting the complexity and multitargeting mechanism of action of photoactivatable prodrugs.^14,15^ Importantly, **Pt1** is able to circumvent cisplatin resistance, maintaining photocytotoxicity in cisplatin-resistant cell lines.^1,12^

Derivatization of **Pt1** at either one or both axial hydroxyl positions can modulate the properties of this class of photoactivatable agents, introducing, for example, cancer-related receptor ligands,^17,18^ increased delivery to cancer cells,^19−21^ and hydrogel formulation.^22^ Conjugation to the axial hydroxide ligands can introduce an additional payload and increase the efficacy of these agents. In particular, the **Pt1** derivative containing coumarin-3 carboxylate **Pt2** (Figure 1)^16^ exhibits increased photocytotoxicity in cancer cell lines in which **Pt1** is only moderately active. Coumarin itself can act as a light-harvesting antenna and also possesses intrinsic anticancer activity. Interestingly, **Pt2** generates blue fluorescence upon irradiation (λ_em_= 440 nm), attributable to the formation of fluorescent coumarin species upon photoactivation with 465 nm light in aqueous solution.^16^ UV–vis spectroscopic studies of the photodecomposition of **Pt2** have suggested rapid loss of the azide ligands (within 15 min) upon irradiation with blue light (λ = 420 nm)^16^ but high stability under dark conditions. Likewise, electron paramagnetic resonance (EPR) studies revealed the formation of ^•^OH and ^•^N_3_ radicals upon photoactivation.^16^

**Figure 2:**
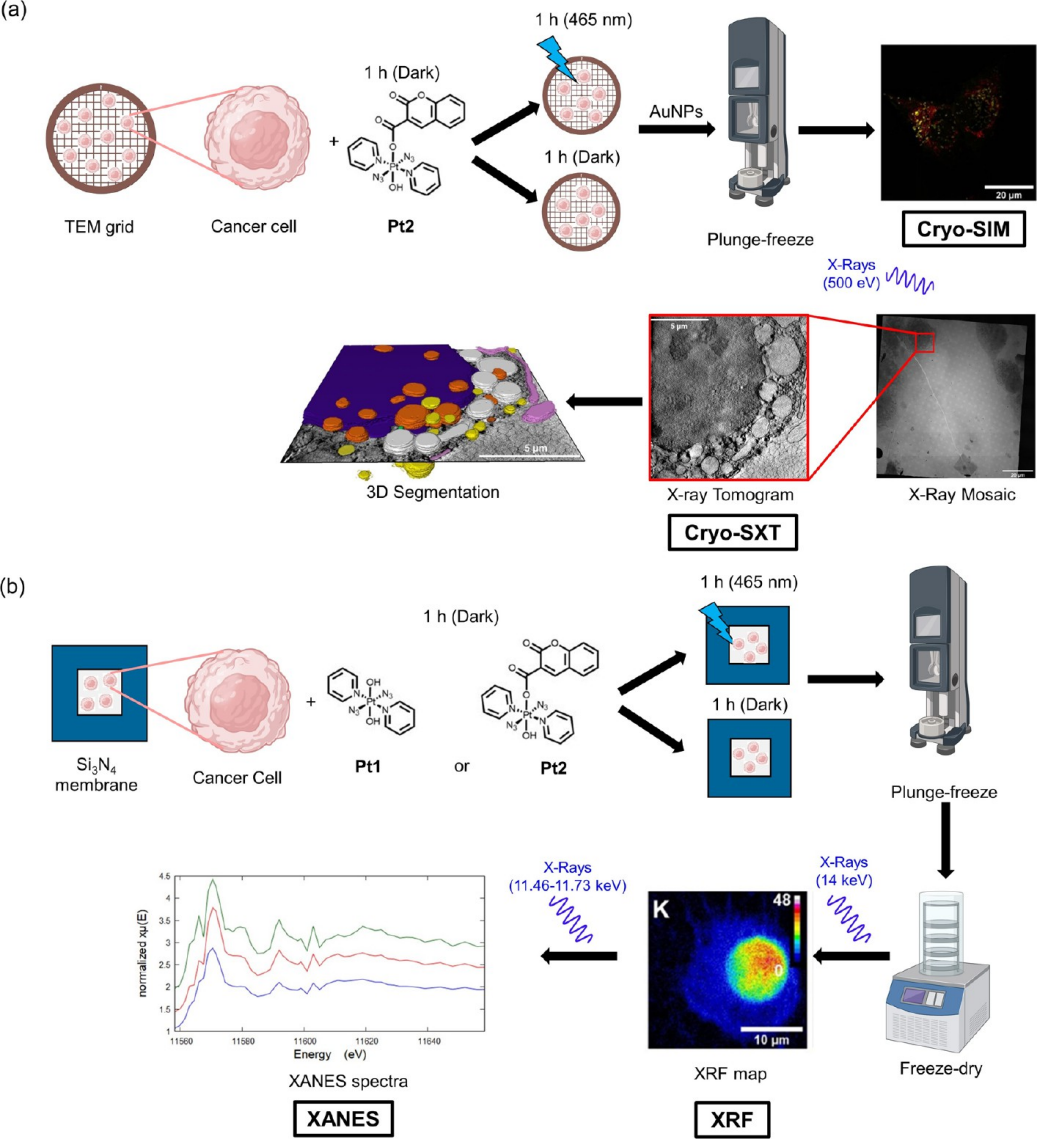
Summary schematic for methods used in this work. (a) cryo-structured illumination microscopy (SIM) and cryo-soft X-ray tomography (cryo-SXT). Cancer cells were grown on TEM carbon–gold grids before exposure to **Pt2** under dark and irradiated conditions then washed with buffer, incubated with fluorophores (MitoTracker and LysoTracker), blotted with gold nanoparticle (AuNP) fiducials (*d* = 250 nm), and plunge-frozen in liquid ethane. These cryopreserved cells were then analyzed by super-resolution fluorescence microscopy (cryo-SIM) down to 200 nm resolution. The same cryopreserved cells were imaged using cryo-SXT using X-rays in the water window (500 eV) to obtain 3D information down to 40 nm resolution. (b) X-ray fluorescence (XRF) and X-ray absorption near edge structure (XANES) spectroscopy. Cancer cells were grown on silicon nitride (Si_3_N_4_) membranes before exposure to **Pt1** or **Pt2** under dark and irradiated conditions then washed with buffer followed by sterile water, blotted, and plunge-frozen in liquid propane–ethane mixture. These cryopreserved samples were then freeze-dried for XRF and XANES analysis at ambient temperature. XRF elemental maps of cells were acquired using hard X-rays (14 keV) above the L_3_M_5_ absorption edge of Pt by raster scanning the nanobeam across the cell in 2D, achieving 100 nm resolution. XANES spectra of Pt in cellular regions were collected by scanning the energy around the Pt L_3_-edge (11.46–11.73 keV) and either averaging the XRF maps at each energy or taking a reduced number of selected energies from which an approximation of the XANES could be extracted. This image was created using biorender.com.

A range of methods, including NMR, mass spectrometry, Raman, and IR techniques, have been employed to investigate the mechanism of photoactivation and cytotoxicity of Pt(IV) azido complexes, including biological assays to identify their cellular targets.^12,15,23,24^ However, the information provided by these studies is limited to chemical models and may differ from the in-cell behavior of these agents in environments which are significantly more complicated. Previously, cellular studies of Pt(IV)-diazido complexes using ^195^Pt have been performed, quantifying the average Pt per cell through analysis of bulk cell pellets.^25^ Here we study, for the first time, their chemistry in single cancer cells in their near-native states using cryo-soft X-ray tomography (Cryo-SXT), nanofocused X-ray fluorescence (XRF), and X-ray absorption near edge structure (XANES) spectroscopy (Table 1, Figure 2).

**Table 1:** Summary of the Techniques Used in This Work

technique	resolution (nm)	information
SIM,^a^ structured illumination microscopy	100	cellular localization of fluorescently labeled organelles^a^
cryo-SXT, cryo-soft X-ray tomography	40	3D imaging of cryopreserved cells to monitor changes in morphology and organelle structure
XRF, X-ray fluorescence	100	2D cellular distribution and quantification of platinum and endogenous elements
XANES, X-ray absorption near edge structure	100	oxidation state of platinum

a Details of fluorophores in Table S1.

In cryo-SXT, cells are illuminated with soft X-rays in the “water” window, the region between the K-absorption edges of carbon (285 eV) and oxygen (543 eV), where carbon-rich biological matter absorbs photons more than the oxygen-rich medium that surrounds it, providing natural contrast for imaging.^26−28^ This allows 3D imaging of vitrified cell populations under cryogenic conditions and the investigation of drug-induced changes in cancer cells at near physiological states, thus avoiding the need to use chemical or mechanical treatments.^29−31^ Cryo-SXT has previously been used to probe drug-induced morphological changes to cancer cells treated with iridium complexes,^32,33^ iron nanoparticles,^34^ and cisplatin (in combination with other chemotherapeutics),^35^ allowing the monitoring of cancer-related cellular events which cannot be achieved using conventional light or electron microscopy.^36^ Complementary to this, structured illumination microscopy (SIM) is a powerful 3D imaging technique that pushes the resolution of optical microscopy past the Abbe diffraction limit,^37−39^ and under cryogenic conditions can provide high resolution fluorescence imaging of cells in their near-native state, and high sensitivity and contrast with minimal reconstruction artifacts.^40^ Cryo-SIM provides a facile route to 3D fluorescence imaging, as it offers (a) a doubling in resolution gain with respect to the best achievable, given the numerical aperture of the objective and the diffraction limit of the light used, (b) a large field of view (170 μm^2^ in this case), (c) low photobleaching, (d) rapid collection times (a few minutes for dual channel acquisition over depths of >10 μm),^41^ (e) processing with standards-guided reconstruction, and (f) data which are largely impervious to severe artifacts (provided the setup is properly calibrated). The primary advantage of the method within the context of this study, is that the setup at beamline B24 has been constructed specifically to accommodate cryopreserved samples that can be used to collect further data in other microscopes such as the cryo-SXT microscope and, therefore, allow the unambiguous association of variable-contrast imaging data from the same sample.

Synchrotron-XRF can be used to investigate the biodistribution of the heavy metal components of metallodrugs in cancer cells (in addition to endogenous biological elements). The use of synchrotron-XRF for the analysis of metal anticancer complexes *in vitro* and *ex vivo* (e.g., in tissue from xenograft models) has been reported for platinum,^44−46^ ruthenium,^47,48^ iridium,^33^ and osmium^49−52^ anticancer agents, among others. Notably, cryo-XRF is emerging at various beamlines for the analysis of frozen-hydrated cells or tissues close to their native state, including ID16A (ESRF, Grenoble)^33^ and 9-ID-B (APS, Illinois).^53^

Complementary to XRF, X-ray absorption spectroscopy (XAS) can be used to gain insights into the oxidation state, speciation, and coordination environments of metals to elucidate their in-cell chemical forms and their potential targets or binding sites. In particular, XANES monitors the region within ca. 50–100 eV of the absorption edge. XANES has been used to investigate the *in vitro* and *ex vivo* speciation of metal complexes in cancer cells, including platinum,^54^ ruthenium,^54^ and osmium, among others.^55^ Such studies include Pt^IV^-prodrugs of cisplatin,^56^ ruthenium clinical candidate drug **KP1019**,^57^ and half-sandwich arene Os^II^ azopyridine complex **FY26**.^58^ In addition, *in situ* chemical reactions between metallodrugs and biomolecules (e.g., DNA, GSH, ascorbate and albumin) have been studied for various metallodrugs.^54,55^ More recently, Hambley et al. used XANES spectroscopy to demonstrate the stability of Pt^IV^ prodrugs in human blood serum in addition to their rapid in-cell activation by reduction (to Pt^II^).^59^

Here, we have used high-resolution X-ray imaging methods (namely synchrotron XRF, XANES, and cryo-SXT) in conjunction with super-resolution visible-light fluorescence imaging (cryo-SIM)^42^ to elucidate the behavior of photoactivatable *all*-*trans* diazido, dihydroxido, dipyridine Pt(IV) complex **Pt1** and its monoaxial coumarin carboxylate conjugate **Pt2** in single PC3 human prostate cancer cells (Figure 2). We have investigated their effects on cancer cell structure, their subcellular localization, and Pt oxidation states, without and with blue light irradiation. This combination of methods allowed studies of the intracellular distribution of the complexes and their effects on single cells in a state close to their native cellular environment, by analysis of either intact frozen-hydrated cells using cryo-facilities^28^ or of dehydrated cryo-fixed cells.^60^ This appears to be the first study of in-cell behavior of photoactivated platinum pro-drugs using these three X-ray technqiues.

## Results

### Antiproliferative Activity of **Pt1** and **Pt2**

**Figure 3:**
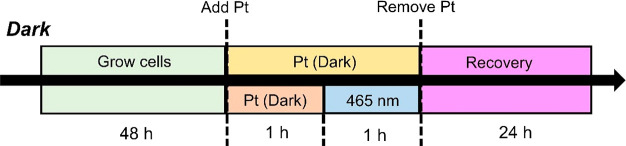
Protocols used to determine the half-maximal inhibitory (IC_50_) concentrations of diazido-Pt(IV) complexes under dark (2 h drug exposure, protected from light) and photoactivated (1 h drug exposure, followed by 1 h irradiation with 465 nm light).

First, we compared the cytotoxicity of these complexes toward PC3 (human prostate) adenocarcinoma cells in the dark and upon irradiation with visible light. Half-maximal inhibitory concentrations in the absence of irradiation (DarkIC_50_) were determined by treating PC3 cells for 2 h protected from light (Figure 3) and then under irradiation (PhotoIC_50_/μM) by treating cells for 1 h (protected from light) followed by 1 h irradiation with blue light (465 nm, 17 J/cm^2^). The coumarin complex **Pt2** was non-toxic to PC3 prostate cancer cells under dark conditions but exhibited high potency (IC_50_ = 6.48 ± 0.84 μM) upon irradiation, ca. 9× higher photocytotoxicity than the dihydroxido complex **Pt1** (Table 2, Figure S1).

**Table 2:** Half-Maximal Inhibitory Concentrations (IC_50_/μM) of **Pt1**, **Pt2**, and Cisplatin toward PC3 Cancer Cells in the Dark and on Exposure to Blue Light, and Standard Deviations from Duplicates of Triplicate Determinations^a^

complex	dark IC_50_^b^ (μM)	photo IC_50_^c^ (μM)
Pt1	>100^d^	55.6 ± 0.9
Pt2	>100^d^	6.48 ± 0.84
cisplatin	>100^d^	>100^d^

a 100 μM concentration (higher than the test range) is deemed inactive.

b 1 h exposure to complexes to allow uptake (protected from light) followed by a further 1 h protected from light and 24 h recovery in fresh medium.

c 1 h exposure to complexes to allow uptake (protected from light) followed by 1 h irradiation with blue light (λ = 465 nm, 17 J/cm^2^) and 24 h recovery in fresh medium.

d Exceeds the concentration range used for IC_50_ determination.

### Cryo-SIM and Cryo-SXT

**Figure 4:**
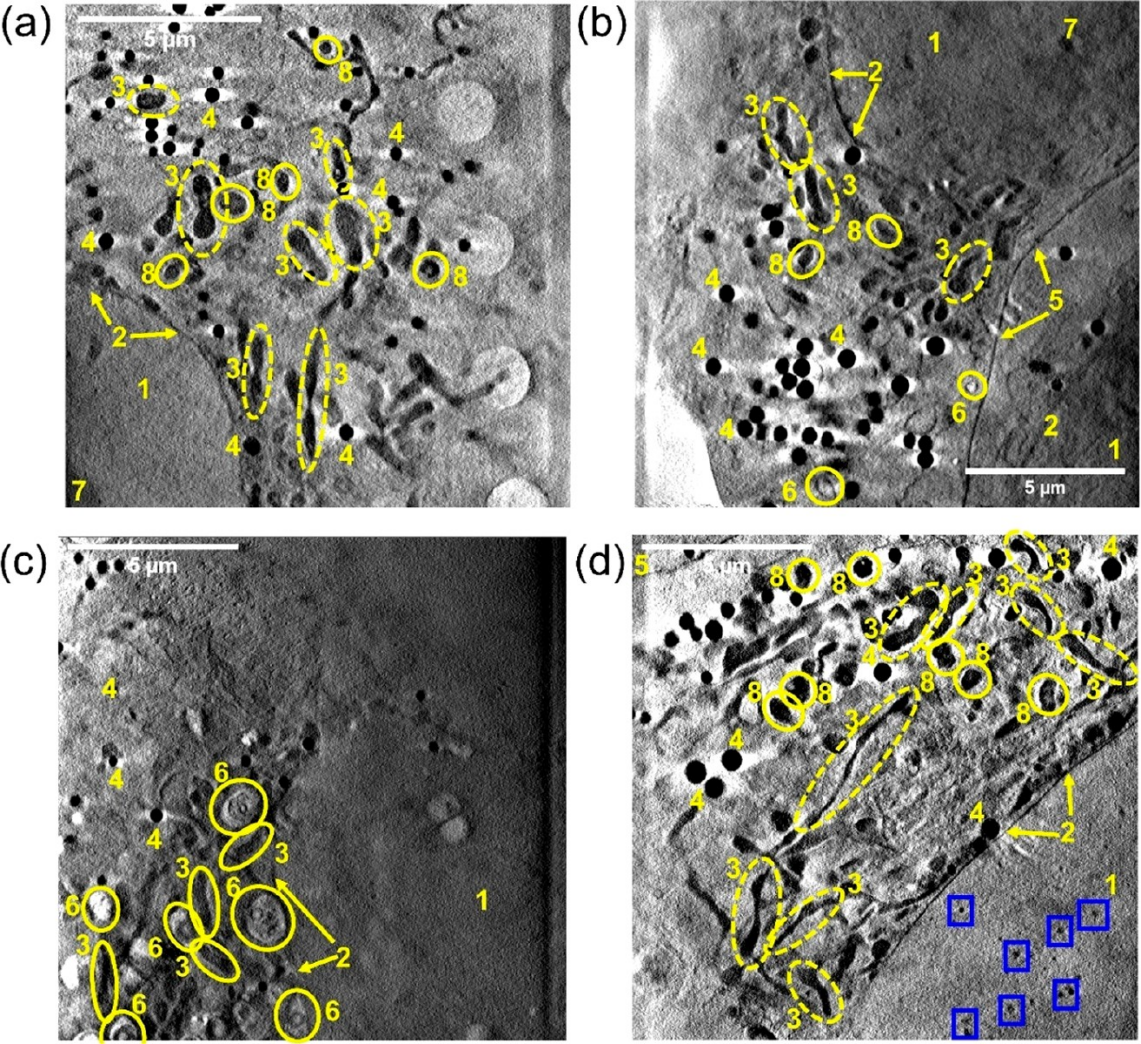
X-ray tomograms of cryopreserved PC3 human prostate cancer cells grown on Quantifoil TEM grids: (a) cell exposed to dark conditions (Figure S12, Video_T1); (b) cell exposed to blue light (465 nm) for 1 h (Figure S13, Video_T4); (c,d) cells exposed to 1× photoIC_50_ (6.5 μM) of **Pt2** for 2 h protected from light (Figures S14, S15, Video_T7, Video_T8). Distinct cellular features: (1) nucleus; (2) nuclear membrane; (3) mitochondria; (4) lipid droplets; (5) plasma membrane; (6) endosomes/lysosomes; (7) nucleolus; (8) dense organelles: Images were generated in IMOD software.^61^ No differences in cell morphology were observed between cells exposed to dark (a) or irradiated conditions (b). Multiple endosomes were observed in (c). Tiny black spots (high X-ray absorption) were observed in the nucleus of the cell mapped in (d) as indicated by blue boxes.

Next, we studied the effects of **Pt2** on PC3 cell morphology on the B24 cryo-SIM microscope and located mitochondria using MitoTracker Deep Red and lysosomes using LysoTracker Red (Figures S2–S11).^39,42^ Cryopreserved PC3 cells incubated with MitoTracker Deep Red (λ_ex/em_ = 644/665 nm) and LysoTracker Red (λ_ex/em_ = 577/590 nm) revealed strong fluorescence emissions, allowing the identification of mitochondrial and lysosomal organelles and subsequently the nucleus (characterized by a lack of fluorescence). Blue fluorescence was not observed in the untreated control cells (dark and light). Additionally, blue fluorescence was not detectable in cells treated with 1× photoIC_50_ (1.6−6.5 μM) of **Pt2** under dark or photoconditions. Detailed 3D structural information on frozen-hydrated PC3 cells grown on carbon–gold TEM grids and treated with 0.25–1 × photoIC_50_ (1.6–6.5 μM) of **Pt2** with or without irradiation (Table 3) was obtained using a full-field X-ray microscope (Figure 4, Figures S12–S23, Table S2), down to a resolution of 40 nm. Typically, it took ca. 20 min to acquire a 15 × 15 μm^2^ tomogram using 0.5° rotation steps and ca. 45 min using 0.2° rotation steps with 1 s exposure.

**Table 3:** Summary of the Drug Exposure, Irradiation, and Recovery Times Used for Cryo-SXT Samples

conditions	**Pt2** dose (μM)	exposure time (h)	irradiation time^a^ (h)	recovery time (h)
1× photoIC_50_	6.5	2		
0.25× photoIC_50_	1.6	1	1	
0.5× photoIC_50_	3.2	1	1	
1× photoIC_50_	6.5	1	1	
1× photoIC_50_	6.5	1	1	2

a Exposure to λ = 465 nm, 17 J/cm^2^

Tomograms of untreated PC3 cells exposed to dark or irradiated conditions showed well-rounded nuclei, clear nuclear membranes, distinct nucleoli, plasma membrane, mitochondria, and lipid droplets (Figures S12 and S13). No differences in cell morphology or ultrastructure were observed between cells exposed to dark or irradiated conditions, Figure 4. Similarly, tomograms of cells treated with 1× photoIC_50_ (6.5 μM) **Pt2** (Figures S14 and S15) under dark conditions revealed features typical of untreated PC3 cells, including well-defined nuclear membranes and fused mitochondrial networks, Figure 4. Additionally, many lipid droplets were observed. Two significant observations were made for cells treated with **Pt2** in the dark, compared to untreated control tomograms (Figures S12 and S14): (i) the presence of endosomes (Figures S14 and S15) and (ii) dark, localized spots in the cell nuclei (Figure 4c,d, Figure S15). This can be seen in two-thirds of the cells imaged, suggesting it might be important for the behavior of the complex under dark conditions.

**Figure 5:**
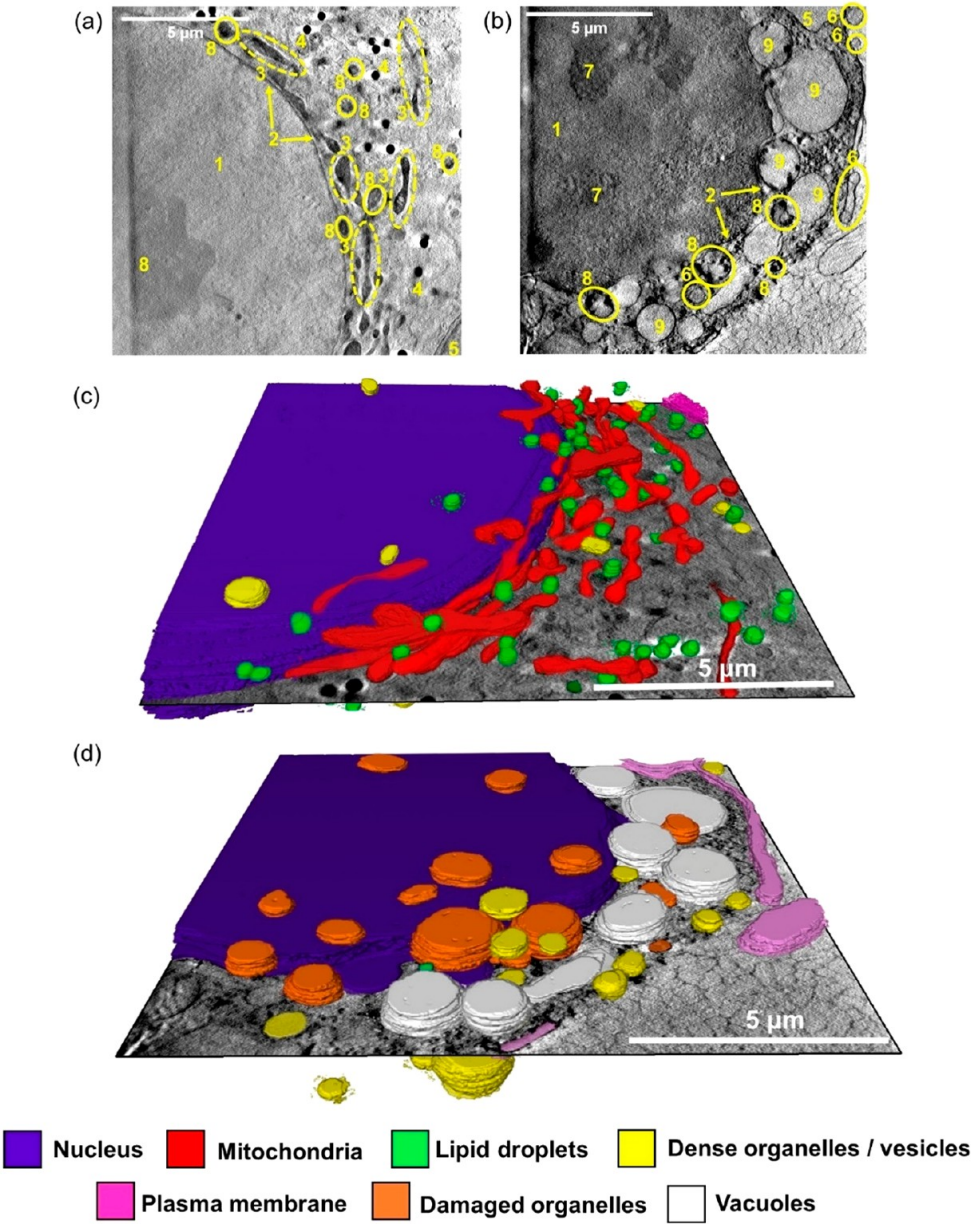
Reconstructed X-ray tomograms and 3D segmented tomograms of two cryopreserved PC3 human prostate cancer cells: untreated control (no drug) under dark conditions (a and c) or exposed to coumarin complex **Pt2** after irradiation with blue light (b and d). (a,b) Reconstructed X-ray of PC3 cells exposed to (a) dark conditions for 2 h (Figure S12, Video_T3), or (b) 1× photoIC_50_ (6.5 μM) **Pt2** for 1 h, followed by 1 h blue light (465 nm, 4.8 mW/cm^2^) irradiation (Figure S21, Video_T16). Distinct cellular features: (1) nucleus; (2) nuclear membrane; (3) mitochondria; (4) lipid droplets; (5) plasma membrane; (6) endosomes/lysosomes; (7) nucleolus; (8) dense organelles; (9) vacuoles. (c-d) 3D segmented tomograms of (a) and (b), respectively (Video_T20 and Video_T21). Images were generated in SuRVoS and visualized in Amira,^62^ showing subcellular features: nucleus (purple); mitochondria (red); lipid droplets (green); dense organelles/vesicles (yellow); plasma membrane (magenta), damaged (unidentifiable) organelles (orange), and vacuoles (white). Significant cellular damage can be observed in (d) compared to the untreated controls (c) including blebbing of the plasma membrane, damage to organelles in the cytoplasm and nuclear membrane, presence of cytoplasmic vacuoles, and reduced number of lipid droplets.

Cells exposed to low concentrations of **Pt2** (0.25× photoIC_50_, 1.6 μM) and irradiated with blue light (Figures S16 and S17) exhibited similar morphologies as the controls, but cytoplasmic vacuoles and membrane-blebbing were observed. A large nuclear vacuole was also observed on treatment with 0.5 × photoIC_50_ (3.2 μM) of **Pt2** (Figures S18 and S19). Significant cellular damage was evident for cells treated with 1× photoIC_50_ (6.5 μM) of **Pt2** and irradiated with blue light (Figure 5b, Figures S20 and S21). Multiple cytoplasmic vacuoles were observed in all the analyzed cells. The outline of the nucleus could be identified but appeared to be damaged, with increased granularity compared to the untreated controls. Mitochondria and lipids were difficult to identify in the cytoplasm, and severe membrane-blebbing at the plasma membrane was evident in the tomogram, Figure S21.

Finally, the morphology of of PC3 cells after recovery in complex-free medium for 2 h after treatment with 1× photoIC_50_ (6.5 μM) **Pt2** for 1 h followed by 1 h irradiation (465 nm) was investigated (Figures S22 and S23). The recovered cells were significantly less damaged than those treated with no recovery. Fused mitochondria, the nuclear membrane and lipid droplets are visible in Figure S22, with very few observable vacuoles. The cell in Figure S23 displayed a relatively healthy morphology, with a significantly high number of lipid droplets observed (total = 88).

Organelles from the reconstructed tomograms were segmented to gain quantitative information on drug-induced morphological changes in 3D. No differences in the size of mitochondria or lipid droplets were observed between all samples analyzed (Tables S3–S5). The sizes of endosomes in two of the PC3 cells treated with **Pt2** under dark conditions (Table S6) were determined to be 0.26 ± 0.13 and 0.33 ± 0.23 μm^3^, respectively. Small, dark spots observed in the cell nucleus of PC3 cells treated with **Pt2** under dark conditions ranged in volume between 0.002 and 0.042 and 0.003–0.031 μm^3^ (Figure S15). Full segmentation of two tomograms (i) untreated control (2 h dark conditions), (ii) 1× photoIC_50_ (6.5 μM) of **Pt2** (1 h + 1 h blue light irradiation) in Figure 5, illustrates the extreme morphological differences in 3D.

### X-ray Fluorescence (XRF) Elemental Mapping

**Figure 6:**
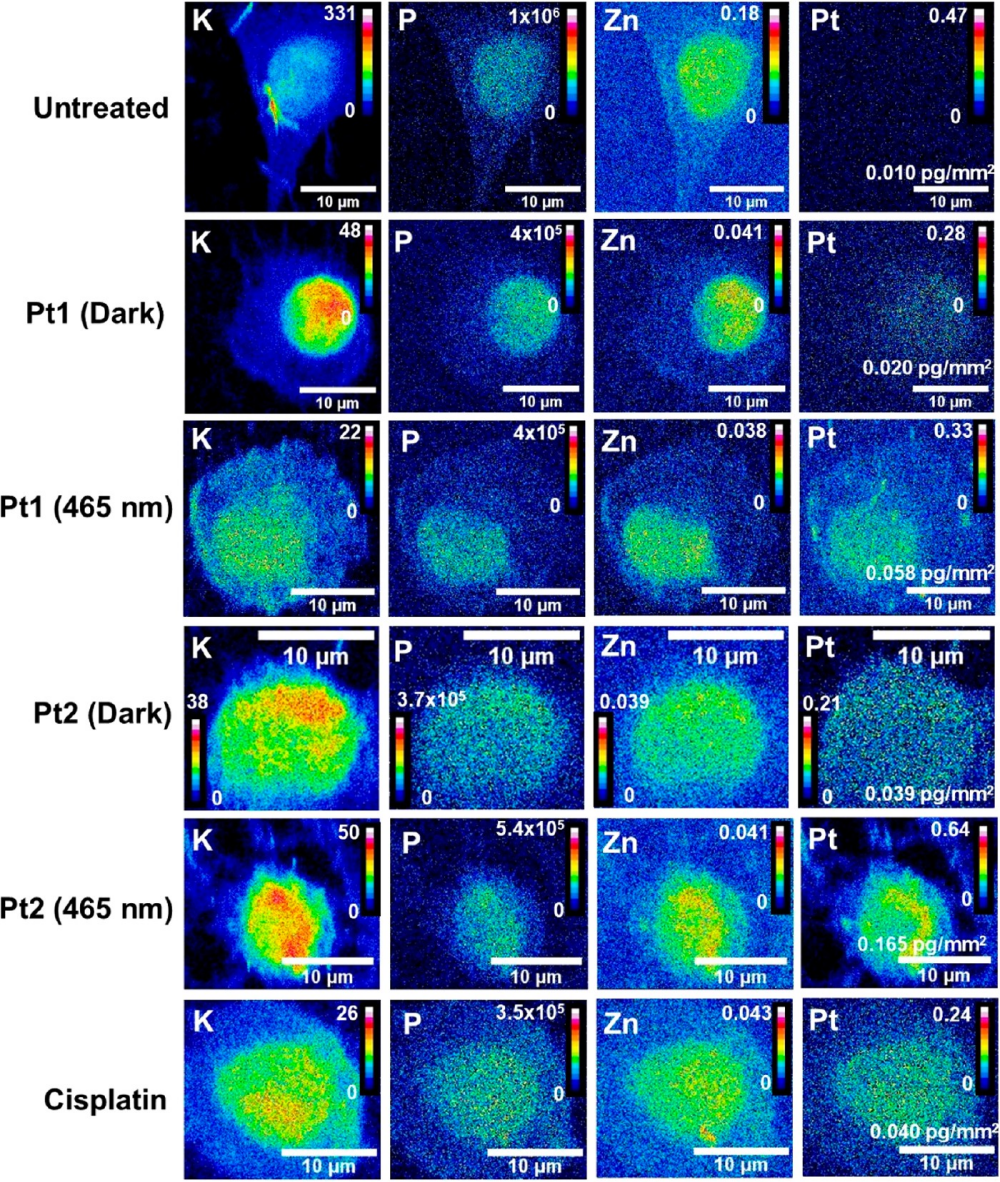
Comparison of the distribution of K, P, Zn, and Pt in whole PC3 human prostate cancer cells before and after treatment with dihydroxido complex **Pt1** or coumarin derivative **Pt2** in the dark and after irradiation with blue light, and cisplatin after irradiation. Synchrotron–XRF elemental maps were analyzed for cryo-fixed and dehydrated PC3 cells before and after treatment with **Pt1** (see also Figures S31–S33) or **Pt2** (Figures S37–39) in the dark and **Pt1** (Figures S34–S36), **Pt2** (Figures S40–S42), and cisplatin after irradiation with blue light (Figures S28–S30), in addition to untreated controls (Figures S25–S27). Conditions: with equipotent (5× photoIC_50_) **Pt1** (275 μM) and **Pt2** (32.5 μM), in the dark (2 h protected from light), or exposed to **Pt1**, **Pt2**, or cisplatin for 1 h, followed by 1 h irradiation with 465 nm light (17 J/cm^2^). Element levels are represented using 16 colors in units of pg/mm^2^ in ImageJ (red = high and blue = low elemental quantities).^69^ Data were acquired using an energy of 14 keV, 0.1 s dwell time, and 100 × 100 nm^2^ step size. Intracellular quantities of Pt in cells treated with **Pt1** or **Pt2** under dark conditions were significantly elevated upon blue light exposure. Intracellular levels of Pt in cells treated with 5× photoIC_50_ of **Pt1** (275 μM) or **Pt2** (32.5 μM) upon irradiation were significantly greater than for cells treated with 5× photoIC_50_ of cisplatin (500 μM) under the same photoconditions.

The intracellular distribution of Pt in cryo-fixed freeze-dried PC3 cells treated with 5× photoIC_50_ of **Pt1** (275 μM) or **Pt2** (32.5 μM) and for comparison, cisplatin with or without irradiation, was determined by monitoring the Pt L_3_M_5_-emission (9.44 keV, Figure S24, Table 4) using an incident energy of 14 keV and 50 × 70 nm beam size, with simultaneous monitoring of the KL_3_ emissions of P, K, S, and Zn (Figure 6, Figures S25–S42). Data were acquired at a spatial resolution of 100 nm. Maps of P, K, and Zn in XRF images were used to locate cell outlines and nuclei of PC3 cells and to correlate their distribution with that of exogenous platinum (Figure 6). Phosphorus is present in the cell in a variety of different forms, from phospholipid bilayers in membranes to the phosphodiester backbone of DNA, RNA, and ATP.^63^ Potassium is the primary cation found intracellularly (ca. 140 mM)^64^ and necessary for the regulation of metabolism and intercellular communication. Intracellular zinc (ca. 0.2–0.3 mM)^65^ plays essential roles in enzyme catalysis, protein regulation, and notably, DNA synthesis. Cell nuclei were readily identified through the presence of highly localized zinc.^66^ Typically, it took ca. 3–4 h to map each cell using a stepsize of 100 nm and 0.1 s exposure.

**Table 4:** Summary of Irradiation and Recovery Times Used for XRF Samples of PC3 Prostate Cancer Cells Treated with 5× PhotoIC_50_ Doses of **Pt1** or **Pt2**

complex	Pt dose (μM)	exposure time (h)	Irradiation time (h)^a^
**Pt1**	275	2	
**Pt1**	275	1	1
**Pt2**	32.5	2	
**Pt2**	32.5	1	1
cisplatin	500	1	1

a Exposure to λ = 465 nm, 17 J/cm^2^

The distribution of intracellular Pt in PC3 cells treated with **Pt1** under dark conditions was difficult to identify due to their low XRF intensity (Figure 6), but showed a moderate colocalization with Zn, as was the case for cisplatin. There was a higher abundance of Pt in cells treated with **Pt2** under dark and irradiated conditions, especially in the nucleus and cytoplasm (Figure 6). Interestingly, colocalization between Pt and Zn was significantly enhanced in cells treated with either **Pt1** or **Pt2** and exposed to blue light compared to dark conditions.

For all treated samples, a moderate colocalization of Pt with Zn, most abundant in the nucleus,^67,68^ was determined using Pearson’s *R*-value (a measure of linearity between two variables, where *R* = 1 is a positive correlation, *R* = 0 no correlation, *R* = −1 negative correlation), Table S10. The colocalization between Pt and Zn was significantly enhanced in cells treated with **Pt1** and exposed to blue light (ranging from *r* = 0.40–0.49; mean = 0.44 ± 0.05) when compared to dark conditions (*r* = 0.19 ± 0.05, *p* = 0.0036). The same trend was observed for cells treated with **Pt2** in the dark (mean *r* = 0.34 ± 0.04) and irradiated (mean *r* = 0.58 ± 0.08; *p* = 0.0423).

Cells treated with **Pt1** under dark or irradiated (465 nm) conditions appeared more rounded (compared to the untreated controls), with spherical nuclei, whereas cells treated with **Pt2** displayed a variety of morphologies (Figures S25–S42). No differences in cell areas (μm^2^) were observed between any of the analyzed cells (*p* > 0.05, Table S7) or in the mean area of nuclei with respect to the whole cell area for cells treated with **Pt1**, **Pt2**, or cisplatin (Table S7).

**Figure 7:**
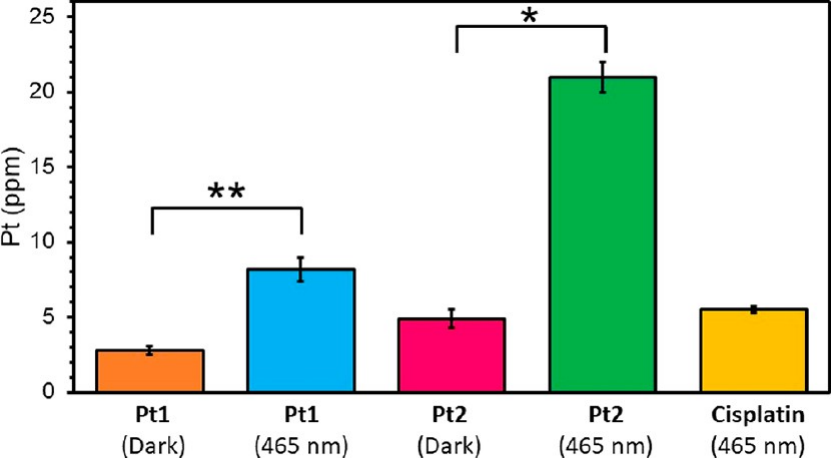
Comparison of the quantities (ppm) of Pt in whole PC3 human prostate cancer cells after treatment with 5× photoIC_50_ of dihydroxido complex **Pt1** (275 μM), coumarin complex **Pt2** (32.5 μM), or cisplatin (500 μM) in the dark and after irradiation with blue light as obtained from XRF. Data were fitted to an AXO calibration standard (elements deposited on a very thin silicon nitride window) in PyMCa (ESRF),^70^ and analyzed using ImageJ.^69^

Mass fraction quantities of Pt were determined for PC3 cells treated with cisplatin, **Pt1**, and **Pt2** by calibrating the flux to an AXO standard (Figure 7, Table S9). PC3 cells treated with **Pt1** under irradiation conditions (465 nm) showed ca. 3× higher Pt levels than cells under dark conditions (*p* = 0.0002). The same trend was observed for **Pt2** under irradiation conditions, with 3.5× more intracellular Pt detected under irradiation with blue light compared to dark conditions (*p* = 0.0001). In general, significantly more Pt was observed in cells treated with Pt complexes under photoconditions when compared with those treated under dark conditions, *p* < 0.01. The mass fraction of Pt in cells treated with cisplatin under irradiation closely resembled that of **Pt2** under dark conditions, despite the significantly lower concentration of **Pt2** used for treatment.

### XANES - Pt Oxidation State

**Figure 8:**
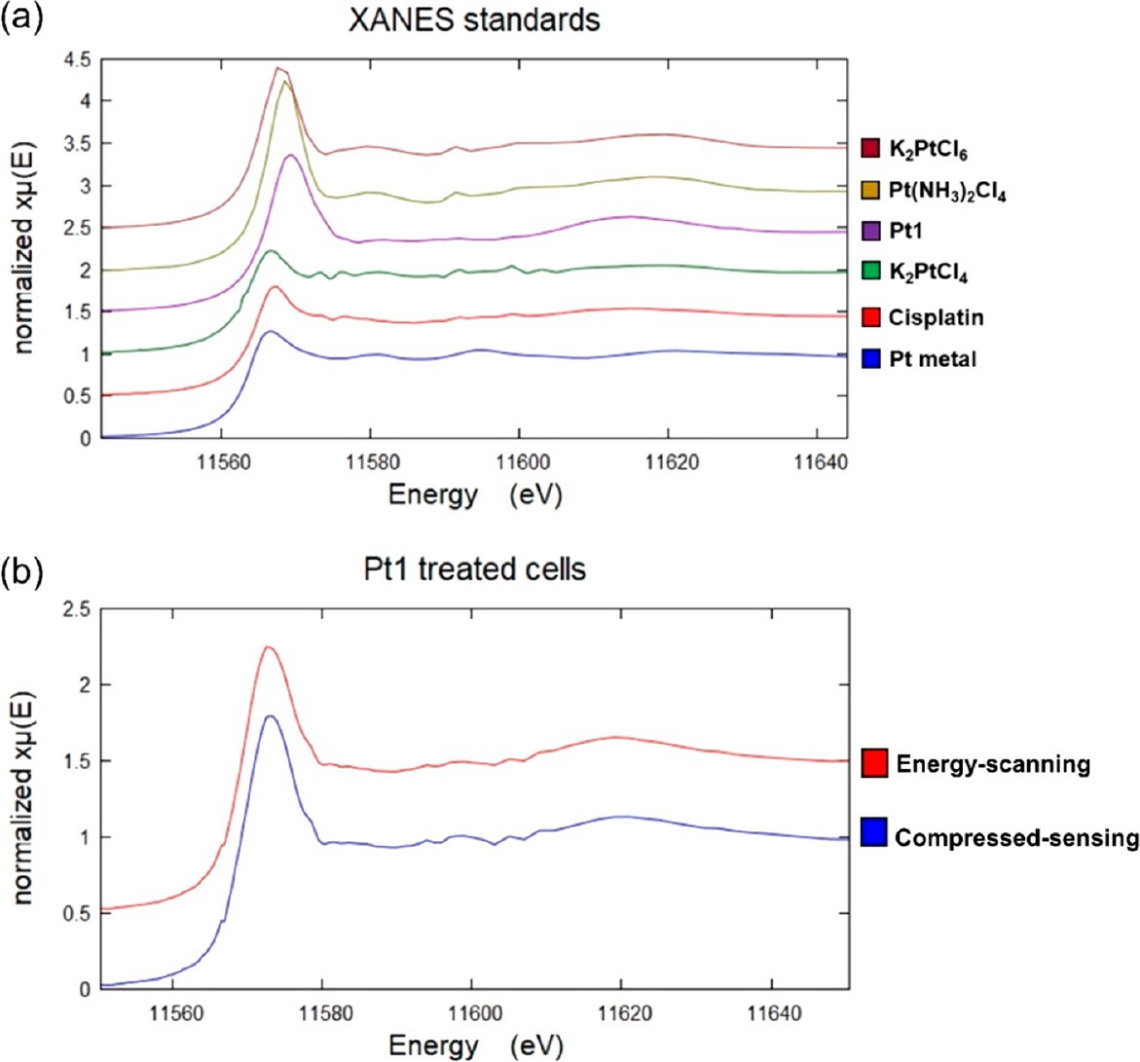
(a) Stacked plot of normalized XANES spectra of solid pellets of K_2_PtCl_6_ (burgundy), Pt(NH_3_)_2_Cl_4_ (yellow), **Pt1** (purple), K_2_PtCl_4_ (green), cisplatin (red), and Pt metal (blue). The spectra were analyzed using Athena XAS Data Analysis Software.^71^ Pre-edge normalization was performed in the energy range 11.47–11.54 keV, and the post-edge normalization was performed in the range 11.59–11.75 keV with a normalization order = 2. (b) Stacked plot of normalized XANES spectra of intracellular Pt in two independent 5 × 5 μm^2^ regions of cryo-fixed and dried PC3 cells treated with 5× photoIC_50_ (275 μM) of **Pt1** (1 h exposure +1 h 465 nm): energy-scanning (red) and compressed-sensing (blue). The spectra were analyzed in Athena XAS Data Analysis Software.^71^ Pre-edge normalization was performed in the energy range 11.47–11.54 keV, and the post-edge normalization was performed in the range 11.59–11.75 keV with a normalization order = 2.

Analysis of the XANES region calibrated by standards (Figure 8a) was used to probe the oxidation state of Pt in PC3 cells treated with 5× photoIC_50_ (32.5 μM) of **Pt2** with or without irradiation (Supporting Information). First, we optimized a compressed-sensing XANES method which selects 8 energy points over the Pt L_3_-edge (11.4692–11.7593 keV). This method is up to 10× faster than traditional energy-scanning and reduces the radiation dose on the cell (see the Supporting Information). In this experiment, the XRF maps at each energy in the range 11.46–11.73 keV were averaged as opposed to obtaining spatially resolved (pixel-by-pixel) maps. The per-pixel spectrum is extremely noisy due to the small region that is being scanned (200 nm step-size; 50 × 70 nm beam size). The averaging of smaller pixels partially removes noise from the spectrum, giving a much higher confidence in the data. As a result, the averaging of spectra provides a ratio of oxidation states (Pt(IV):Pt(II)) for the whole analyzed region (instead of spatially-resolved regions). Optimization for spatially-resolved per-pixel XANES mapping is a planned future development for the I14 beamline.

Two different 5 × 5 μm^2^ Pt-containing regions in cryo-fixed and freeze-dried PC3 cells treated with 5× photoIC_50_ (275 μM) of **Pt1** under blue light conditions, were analyzed using (i) compressed-sensing or (ii) energy-scanning methods (Figure 8b). The energy-scanning and compressed-sensing methods revealed that 76.5 ± 1.7% and 80.3 ± 2.2% using traditional linear combination fitting (LCF), respectively, of the total Pt in the cells was Pt(IV) after treatment with **Pt1** and irradiation with blue light (Table 5, Table S11). This good agreement between compressed-sensing and energy-scanning methods, validates the use of the more rapid compressed-sensing method for XANES analysis.

**Table 5:** Oxidation States, White Line Energies, Normalized Maximal Absorption, and Normalized Peak Ratios between the Normalized Maximum of the White Line (a) and the Post-edge Minimum (b) of Cisplatin, K_2_PtCl_4_, K_2_PtCl_6_, Pt1 Solid Pellets, and Platinum Metal^a^

compound	oxidation state	white line (eV)	peak maximum (*a*)	post-edge minimum (*b*)	normalized peak ratio (*a*/*b*)
Cisplatin	2+	11571.2	1.300 ± 0.001	0.8673 ± 0.0002	1.499 ± 0.002
K_2_PtCl_4_	2+	11570.7	1.226 ± 0.003	0.893 ± 0.003	1.372 ± 0.008
K_2_PtCl_6_	4+	11571.9	1.898 ± 0.003	0.859 ± 0.002	2.21 ± 0.01
Pt(NH_3_)_2_Cl_4_	4+	11572.3	2.237 ± 0.007	0.799 ± 0.006	2.80 ± 0.02
**Pt1**	4+	11573.4	1.861 ± 0.004	0.8198 ± 0.0004	2.270 ± 0.005
Pt metal	0	11570.6	1.271 ± 0.004	0.946 ± 0.009	1.34 ± 0.01
In-cell **Pt1**^b^,^d^	unknown	11572.7	1.60 ± 0.02	0.96 ± 0.02	1.68 ± 0.02
In-cell **Pt1**^c^,^d^	unknown	11572.9	1.65 ± 0.02	0.95 ± 0.02	1.74 ± 0.03

a In addition, two independent 5 × 5 μm^2^ Pt-containing regions in cells treated with independent 5× photoIC_50_ (275 μM) **Pt1** and irradiation were analyzed using traditional energy-scanning and compressed-sensing for method validation of the latter.

b Energy-scanning.

c Compressed-sensing.

d 5 × 5 μm^2^ Pt-containing regions of interest in cryopreserved, freeze-dried PC3 cells treated with **Pt1** under photoconditions (1 h + 1 h 465 nm, 17 J/cm^2^)

**Figure 9:**
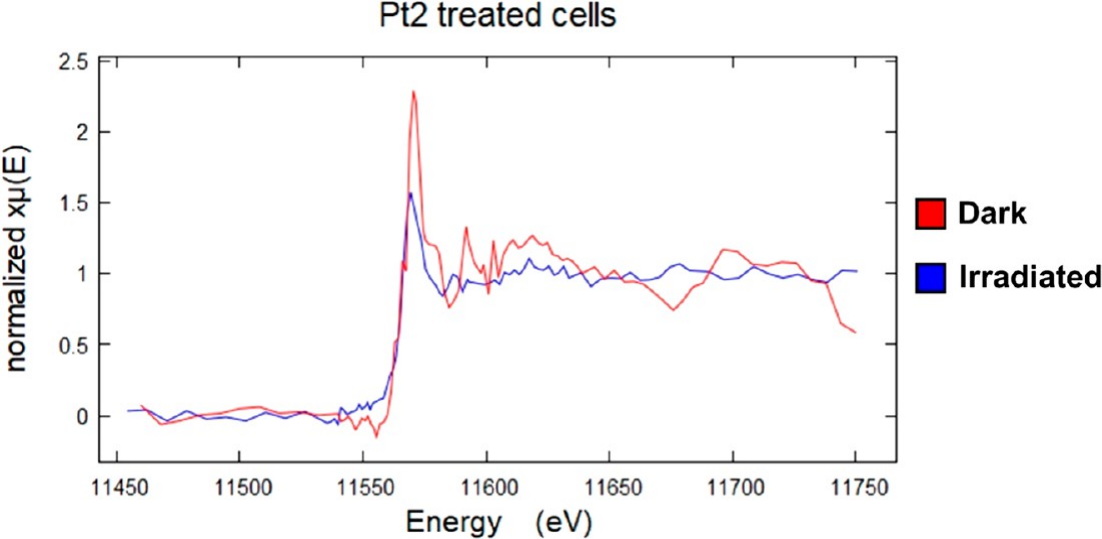
Comparison of X-ray absorption near-edge structure (XANES) features for whole PC3 human prostate cancer cells treated with coumarin complex **Pt2** in the dark or irradiated with blue light, acquired using the compressed-sensing method. Normalized spectra are shown of intracellular Pt in two independent 15 × 15 μm^2^ regions of cryo-fixed and dried PC3 cells treated with 5× photoIC_50_ (32.5 μM) of **Pt2** in the dark (2 h protected from light, red) or irradiated (1 h exposure +1 h 465 nm, 17 J/cm^2^, blue) conditions. Data were acquired using 500 nm step size and 0.4 s dwell time. Spectra were analyzed in Athena XAS Data Analysis Software.^71^ Pre-edge normalization was performed in the energy range 11.47–11.54 keV, and the post-edge normalization was performed in the range 11.59–11.75 keV with a normalization order = 2. A shift in the Pt-edge peak to lower energies is observed upon irradiation with blue light, in addition to a reduction in peak height, suggesting that more Pt(II) is present upon irradiation.

XANES spectra of cryo-fixed and freeze-dried PC3 human prostate cancer cells treated with 5× photoIC_50_ (32.5 μM) of **Pt2** with or without irradiation were recorded using the compressed-sensing method (Figure 9, Figures S43 and S44). Three independent 15 × 15 μm^2^ Pt-containing cellular ROIs per condition (dark and light) were analyzed using either (i) 0.1 s dwell time and 100 nm step size, or (ii) 0.4 s dwell time and 500 nm step size, and analyzed initially using LCF (Table S12).

**Figure 10:**
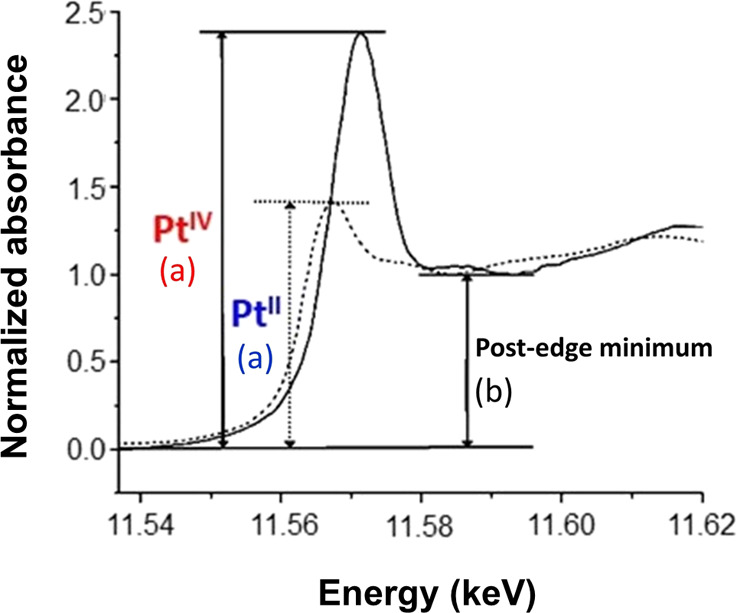
XANES spectra of Pt^IV^ and Pt^II^ compounds showing the maximum peak absorption (a) for each oxidation state and the post-edge minimum (b). Adapted from ref (73). Copyright 2003 American Chemical Society as modified from a literature figure.

It is difficult to determine the relative proportions of Pt(II)/Pt(IV) in a mixture by conventional curve-fitting techniques such as LCF due to the similarities in peak energies and shapes. To overcome this, Hambley et al. proposed a method which normalizes the maximum absorption of the edge (height of peak, *a*) with the post-edge minimum (*b*) immediately after the white line (Figure 10, Table 5).^56,72^ This results in a linear relationship between the *a*/*b* ratio and the proportion of Pt(IV) in a mixture: the greater the ratio, the greater the amount of Pt(IV).^56,72^

For cells treated with **Pt2** under dark conditions, this analysis gave *a*/*b* ratios of 2.36 ± 0.01 and 2.97 ± 0.02 for the two regions analyzed at higher resolution (100 nm stepsize, 0.1 s) and 2.891 ± 0.002 for the single region analyzed at lower resolution (500 nm stepsize, 0.4 s), Table 6. For cells treated with **Pt2** and irradiated, the *a*/*b* ratios were 2.16 ± 0.02 and 2.38 ± 0.01 for the two regions analyzed at higher resolution (100 nm stepsize, 0.1 s), and 1.84 ± 0.01 for the single region analyzed at lower resolution (500 nm stepsize, 0.4 s), Table 6. The lower *a*/*b* ratio observed for treated cells upon irradiation (ca. 22% lower) clearly indicates that irradiation increases the amount of Pt(II) in the cells compared to cells treated in dark conditions. Calibration with standards (Figure S45) gives a 27% decrease in Pt(IV) on irradiation, which is in reasonable agreement.

**Table 6:** Normalized Maximum Absorption and Normalized Peak Ratios between the White Line (a) and the Post-edge Minimum (b) of Cryofixed and Dehydrated PC3 (Human Prostate) Cancer Cells Treated with 5× PhotoIC_50_ (32.5 μM) of **Pt2** under Dark (2 h) or Blue Light Conditions (1 h 465 nm +1 h, 17 J/cm^2^), As Determined from Their Normalized XANES Spectra

conditions	dwell time (s)	step size (nm)	peak maximum (*a*)	post-edge minimum (*b*)	*a*/*b*	average *a*/*b*
dark	0.1	100	1.877 ± 0.001	1.795 ± 0.004	2.36 ± 0.01	2.74 ± 0.05
	0.1	100	2.287 ± 0.001	0.771 ± 0.005	2.97 ± 0.02	
	0.4	500	2.426 ± 0.004	0.839 ± 0.002	2.891 ± 0.002	
light	0.1	100	1.899 ± 0.007	0.880 ± 0.005	2.16 ± 0.02	2.13 ± 0.04
	0.1	100	1.984 ± 0.005	0.835 ± 0.003	2.38 ± 0.01	
	0.4	500	1.567 ± 0.006	1.850 ± 0.003	1.84 ± 0.01	

## Discussion

Platinum(IV) azido complexes are a promising class of photoactivated anticancer agents that can be activated using visible light and do not depend on the presence of oxygen for activity,^74−77^ unlike conventional photodynamic therapy agents, which rely on singlet oxygen generation. While these azido Pt(IV) complexes are inert in the dark, they become toxic to cancer cells upon irradiation with visible light, showing no cross-resistance with cisplatin.

Photocytotoxicity of the prototype dihydroxido complex **Pt1** can be enhanced by fine tailoring its axial substituent(s) with (i) light-harvesting antennae, (ii) cytotoxic moieties that can be released upon photoactivation, or (iii) groups that increase cellular accumulation. Notably, the coumarin substituent in **Pt2** can act both as a light-harvesting antenna and as an anticancer agent. In addition, it increases cancer cell accumulation of the platinum complexes by increasing their hydrophobicity. **Pt2** exhibits higher photocytotoxicity compared to **Pt1** for all the cancer cell lines tested,^16^ the difference being particularly prominent for the PC3 human prostate cancer cell line investigated in this work (Table 2).

Previous chemical characterization of Pt(IV) azido complexes/photoproducts and analysis of their behavior in the presence of biomolecules has shown that these prodrugs release reactive Pt(II) species and radicals upon photoreduction by light irradiation, which can interact with both proteins and DNA.^12,13,78,79^ In the present study, we have used synchrotron techniques to provide new insights into the intracellular photoactivation of Pt(IV) azido complexes in single cancer cells. We analyzed the morphological changes in cells exposed to the coumarin complex **Pt2** in the dark and upon light irradiation using cryo-SXT, and correlated them to the cellular distribution of platinum in both conditions in comparison with the dihydroxido complex **Pt1** and the clinically established drug cisplatin using XRF. We also used XANES to probe the intracellular photoreduction of **Pt2.**

### Morphological Changes to Cancer Cells

Analysis of cells using cryo-SIM revealed no significant morphological differences between cells treated with 1× IC_50_ (6.5 μM) of **Pt2** and exposed to blue light compared to the untreated controls. **Pt2** forms blue fluorescent coumarin radicals upon photoactivation with light (λ_ex/em_ = 405/450 nm);^16^ however, the blue fluorescence could not be observed by cryo-SIM. This implies that, although **Pt2** can generate blue fluorescence in aqueous solution (from 7-hydroxycoumarin-3-carboxylate, formed by reaction of hydroxyl radicals with released coumarin-3-carboxylate)^16^ the fluorescence inside cells is likely not strong enough. Our observations using the B24 cryo-SIM suggest that PC3 cells have a sparse population of lysosomes under physiological conditions, which remain relatively dispersed unless there is extensive cell–cell contact, whereupon a clustering behavior is observed. When the cells were exposed to the compound before activation, the incidence of lysosomal vesicles increased and appeared to polarize in a perinuclear distribution. Activation apparently reinforced this trait, but noticeably, in some cases, it also contracted the overall cell volume (a common response to stress, whereupon a cell retracts cytoplasmic adherence-based expansions and becomes more spherical). Mitochondrial distribution remains largely unaffected by the presence of the inert compound, but appears to be concentrated in smaller enclaves once the compound is activated.

X-ray tomograms were acquired at a remarkable resolution of 40 nm under cryogenic conditions, allowing distinction between organelles and subcellular features in 3D. No significant differences were observed between untreated PC3 prostate cancer cells in the dark and those which had been irradiated. This correlates with previous cryo-SXT data for cancer cells exposed to blue light of this intensity^32^ and confirmed that 1 h exposure to blue light of this dosage (465 nm, 17 J/cm^2^) does not damage the cellular integrity (correlating with cell viability assays).^16^ Tomograms of untreated PC3 cells exhibited well-rounded nuclei, clear nuclear membranes, nucleoli, plasma membrane, mitochondria, and lipid droplets (Figure 4a,b, Figures S12 and S13). A recurring feature for this cell line is the presence of lipid droplets (0.05–0.38 μm^3^ in volume). Cells store lipids for membrane synthesis in the form of droplets, which can play roles in disease progression.^80−84^ Additionally, exosome or vesicle-shedding at plasma membranes was observed, a prevalent trait in many prostate cancer cell lines.^83,85−88^ Overall, PC3 cells incubated in Pt-free medium and kept under dark conditions showed subcellular features typical of this cell line.^89,90^

Similarly, no significant differences were observed for cells treated with **Pt2** in the dark, in good agreement with the limited dark cytotoxicity of **Pt2**. However, these cells presented cytoplasmic, dense endosomal-like structures that were not seen for untreated cells and thus can be attributed to treatment. Endosomes were identified in two independent cells treated with **Pt2** under dark conditions (0.14–0.99 μm^3^ in volume, average: 0.29 ± 0.18 μm^3^), and exhibited a variety of dense or lucent interiors, in agreement with literature cryo-SXT characterizations of endosomes.^35,43^ Some of these endosomal-structures are dark in appearance, which may be indicative of internalized platinum due to the enhanced X-ray absorption of heavy metals compared to endogenous elements. Importantly, diazido-Pt(IV) complexes are relatively inert in the dark. It is therefore plausible that under dark conditions, the cells can remove xenobiotic metal species from intracellular milieu via endosomal-mediated pathways.^91,92^ In addition, small, dark spots in the cell nuclei were observed for cells treated with **Pt2** under dark conditions. Nuclear inclusion bodies arising from administration of bismuth and lead compounds are well described in the literature^93,94^ and are thought to provide protection as inert chemical forms. The presence of small highly absorbing areas (<0.04 μm^3^) in cell nuclei strongly suggests there is uptake and concentration of Pt within nuclei of cells exposed to **Pt2**.

In contrast, when PC3 cells were irradiated with blue light after treatment with **Pt2**, dramatic changes in cell morphology were visible and, notably, were concentration-dependent. Cytoplasmic vacuoles, traditionally associated with ROS damage^95^ and non-apoptotic cell death,^96^ were visible even at the lowest concentration (0.25× photoIC_50_, 1.6 μM). These observations are in agreement with the production of N_2_ and ROS (perhaps arising from chain reactions of released azidyl radicals) upon irradiation of this class of complexes^12,24,74,97^ and with their cell death mechanisms, likely involving autophagy and immunogenic cell death (ICD).^98^ Interestingly, a large nuclear vacuole surrounded by lipid droplets was observed in a cell treated with 0.5× photoIC_50_ (3.2 μM) of **Pt2** with irradiation (Figure S19, **T14**). The physiological significance of nuclear vacuoles is not clear, but it likely reflects morphological changes induced by **Pt2**, with the congregating lipid droplets recruited to repair damage and ensure cell survival.

The effects of treatment became more pronounced at increasing **Pt2** concentrations. In particular, cells treated with 1× photoIC_50_ (6.5 μM) **Pt2** upon irradiation displayed a dramatic change of their morphology. These cells appeared somewhat deflated, perhaps due to cytoskeletal disintegration^99^ or cell death prior to cryopreservation, with undefined mitochondria, lipids, and vesicles, multiple cytoplasmic vacuoles, and extensive plasma membrane-blebbing (Figure 5b).

Likewise, membrane-blebbing and vesicle-shedding were observed upon irradiation of cells treated with even the lowest concentration of **Pt2** (0.25 × photoIC_50_, 1.6 μM), and this became more pronounced at higher concentrations. Irradiation of cells exposed to higher **Pt2** concentration (1× photoIC_50_, 6.5 μM) caused extensive membrane-blebbing and vesicle-shedding, highlighting the dose-dependent effect of irradiated **Pt2**. Furthermore, this suggests that cellular mechanisms associated with membrane-blebbing such as apoptosis or necrosis,^100^ may also contribute to the overall antiproliferative effects of **Pt2**.

Organelle deterioration was also evident (Figure 5b), making the identification of mitochondria, lipid droplets and other vesicles difficult. As this family of diazido-Pt^IV^ complexes can generate toxic azidyl and hydroxyl radicals upon irradiation,^23,74^ the observed damage may be associated with targeting of mitochondria and other organelles. It is likely that **Pt2** exerts a multitargeted anticancer effect on nuclear DNA and other organelles, by generating toxic radicals upon photoactivation.

In addition to organelle damage, increased granularity of cell nuclei was observed for cells after treatment with **Pt2** and irradiation. This can be associated with growth arrest and cell death (particularly autophagy) in response to chemotherapeutics or irradiation,^101^ as previously shown for organic chemotype compounds in LNCaP (Lymph Node Carcinoma of the Prostate) and PC3 cells.^101^ Thus, potentially the formation of nuclear stress granules in response to treatment with **Pt2** might implicate autophagic cell death upon photoactivation. This is in strong agreement with previous studies on photoactivation of the mixed amine ligand complex *trans*,*trans*,*trans*-[Pt^IV^(N_3_)_2_(OH)_2_(py)(NH_3_)], for which increased levels of autophagic proteins were detected in cancer cells,^102^ implicating autophagy in cell death. Equally, the possible autophagy observed here is consistent with reported chemotherapeutically-stimulated ICD induced in cancer cells by photoactivated **Pt1**.^98^

Interestingly, when allowed to recover after drug removal, cells that survived treatment showed healthy nuclei, well-defined lipid droplets and fused mitochondria, and noncompromised plasma membranes. This suggests that, when it does not result in cell death, the effect of PACT can be reverted by cell repair mechanisms. The variation observed in the number of lipid droplets is likely to be a consequence of different stages of cell growth or recovery in response to **Pt2**. The observed cell repair may also implicate a cytostatic (growth-inhibition) contribution to the antiproliferative activity of **Pt2** by altering the rate of cell growth in response to damage, an aspect worthy of further investigation.

Similar to observations from cryo-SXT, untreated PC3 cells imaged by XRF displayed stretched (cobblestone) morphologies (ca. 30–40 μm in length), typical of this cell line.^90,104,105^ This suggests that despite the different state of the samples, cryopreserved versus cryofixed-dehydrated for XRF, the cellular environment remains comparable for both techniques. Cells treated with cisplatin, **Pt1**, or **Pt2** and imaged by XRF did not display any significant differences in size or roundness factor, with the cells predominantly exhibiting stretched-out morphologies typical of this cell line. However, these cell populations were heterogeneous with higher levels of intracellular Pt associated with “rounding” of cells, which may arise from a cellular stress response and programmed cell death.^106^

### Accumulation and Distribution of Pt-diazido PACT Complexes in Cancer Cells

XRF elemental maps were acquired at a spatial resolution of 100 nm, allowing subcellular information to be obtained. For all treated samples, a moderate colocalization of Pt with Zn, most abundant in the nucleus,^67,68^ was observed, while the mean proportion of cellular Pt from **Pt1**, **Pt2** localizing in the nuclei was >30% (Table S9), regardless of the photoconditions. This is consistent with the proposed mechanisms of action involving DNA binding.

While irradiation with blue light had little effect on the dimensions and roundness of cells treated with these Pt complexes, dramatic changes in the cellular accumulation of Pt for both **Pt1** and, especially, **Pt2** were observed. Blue light irradiation increased the accumulation of Pt from **Pt1** by ca. 3× (Figure 7) and for **Pt2** by ca. 3.5× compared to analogous cells protected from the light (Figure 7). In addition to enhanced Pt retention upon irradiation, increased intracellular levels of Pt may also arise from photoinduced membrane damage. Free radicals can alter the physiology of cell membranes,^107,108^ and particularly, hydroxyl radicals (^•^OH) can modulate the membrane permeability of cells.^109^ As diazido-Pt(IV) complexes can generate hydroxyl and azidyl (N_3_^•^) radicals upon irradiation and the antiproliferative screening protocol involves irradiation while **Pt1** or **Pt2** are still present in the cell growth medium, their extracellular formation may give rise to increased cell permeation of the photoproducts.^110^ This correlates with the severe membrane damage observed in cells treated with photoactivated **Pt2** by cryo-SXT. Thus, the extracellular activation of such Pt(IV)-diazido prodrugs by light might provide a means of killing cancer cells via radical-induced membrane damage, in addition to forming Pt(II) species which can bind to DNA. Providing the drug can reach the tumor microenvironment, this might afford a combined extra- and intracellular mechanism to overcome resistance.

Interestingly, cells treated with the more potent coumarin conjugate **Pt2** accumulated >2× more Pt than cells treated with **Pt1** under photoconditions, despite the 8.5× lower concentration used, and thus, increased cellular uptake may play an important role in the enhanced photocytotoxicity of **Pt2**. This also correlates with ICP-MS cellular accumulation studies of **Pt1** and **Pt2** in A2780 (ovarian) and A549 (lung) cancer cells, which revealed significantly higher cellular quantities of Pt for the coumarin derivative.^16^

Overall, these data suggest that blue light not only activates both **Pt1** and **Pt2** intracellularly, but likely promotes cellular Pt uptake/retention by reduction of inert Pt(IV) to more reactive Pt(II) species as well as photoinduced reactions with biomolecules. This dark/light difference in uptake has been reported for Pt levels in human 5637 bladder cancer cells for structurally similar diazido dihydroxido pyridine/NH_3_ and cyclohexylamine/NH_3_ Pt(IV) complexes upon irradiation with UVA light, where intracellular Pt levels were up to 20 ng/10^6^ cells after 2 h, almost 20× greater than under dark conditions.^25,79^

### In-Cell Photoreduction of Pt(IV)

XANES spectra of cisplatin and K_2_PtCl_4_ as Pt(II) standards were comparable as expected, and those of the Pt(IV) standards (K_2_PtCl_6_, Pt(NH_3_)_2_Cl_4_, and **Pt1**) had more intense absorption edges, in agreement with previous reports on Pt(II) and Pt(IV) complexes.^72^

XANES spectra of PC3 cells treated with 5× photoIC_50_ (32.5 μM) of **Pt2** with or without irradiation analyzed with 100 or 500 nm step size, at 0.1 or 0.4 s exposure, respectively, were obtained on a Diamond beamline I14 using a new compressed-sensing approach instead of the traditional data acquisition method of scanning the energy with the monochromator (which is highly time-consuming at the nanoscale). This new method involved acquiring data at only 8 selected energies in the energy range, as opposed to the full range in energy-scanning, allowing ca. 10× faster XANES analysis and reduced possible beam damage caused during the measurements. Pixel-by-pixel spatial resolution was not obtained because of the extreme noise due to the small region being scanned (200 nm step-size; 50 × 70 nm beam size); thus, the spectra were averaged instead.

Using the normalized peak height method developed by Hambley et al., the *a*/*b* ratios found for irradiated cells was ca. 22% lower than that of cells treated in the dark. Similarly, a 27% reduction of Pt(IV) to Pt(II) inside the cells was observed based on the *a*/*b* ratios from the Pt(II) and Pt(IV) standards (Figure S45). This extent of formation of Pt(II) may be sufficient to exert a strong cytotoxic action. However, it is also possible that both Pt(II) and Pt(IV) species contribute to the photochemotherapeutic mechanism action of diazido-Pt(IV) prodrugs. Interestingly, Pt(IV) photoproducts were observed in previous ^195^Pt NMR studies of **Pt1** irradiated in the presence of 5′-guanosine monophosphate, which appeared to arise from partial reoxidation after complete photoreduction to Pt(II) after irradiation.^12^

Overall, these results highlight how synchrotron techniques such as nanofocused XANES can provide new insights into the chemistry of photactivated diazido-Pt(IV) prodrugs inside cancer cells. Our studies highlight the heterogeneous nature of cell compartments and the difficulty of modeling reactions of these photoactivated prodrugs *in vitro*.

## Conclusions

**Figure 11:**
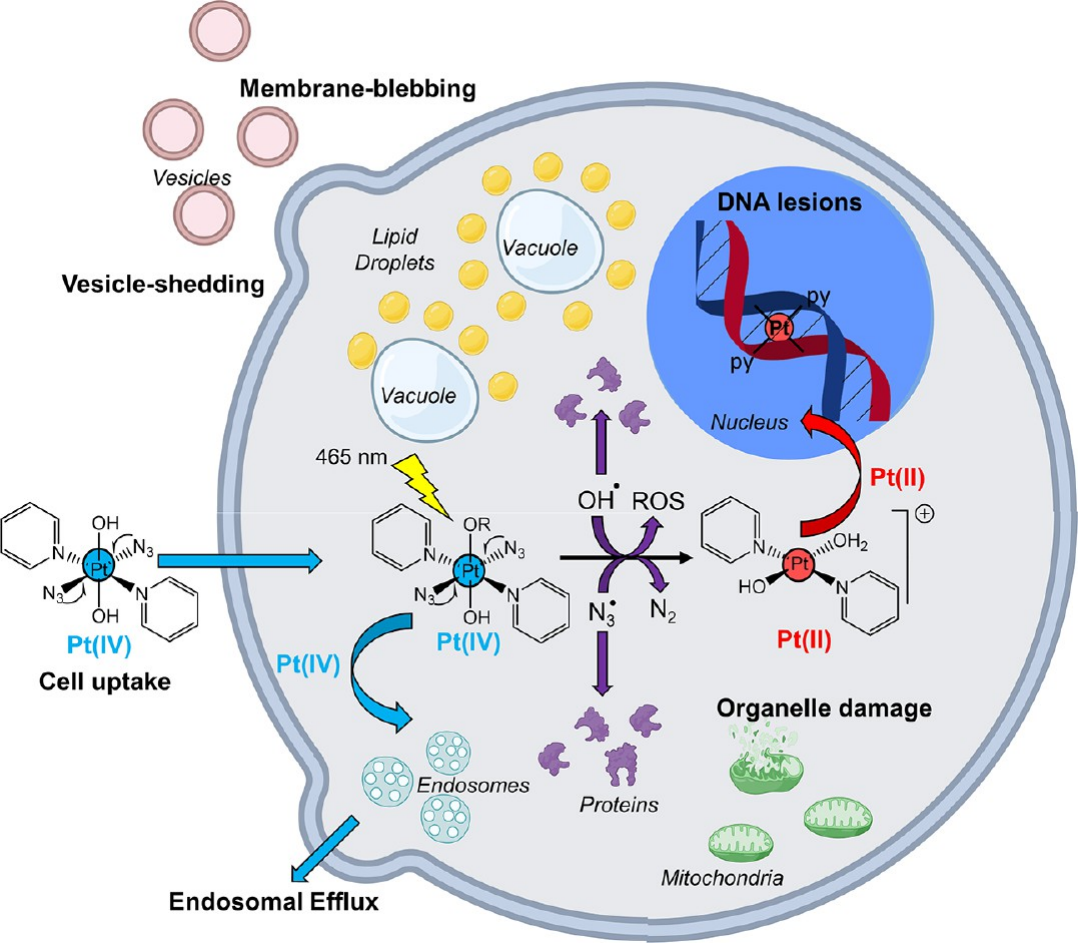
Pictorial illustration of possible cellular events arising from photoactivation of diazido Pt(IV) prodrugs in cancer cells with blue light. Created with biorender.com.

We have combined cryo-SXT, XRF, and XANES for the first time to investigate the anticancer mechanism of action of photoactivatable Pt(IV) PACT agents **Pt1** and **Pt2** in cancer cells in their near-native state. Application of such techniques provided complementary information at subcellular resolution on the biological response of cancer cells to PACT treatment (cryo-SXT) and the cellular distribution and oxidation state of the photoactive complexes and their photoproducts (XRF and XANES), as summarized in Figure 11.

Under dark conditions, diazido-Pt(IV) complexes are sparsely distributed in cancer cells (including the nucleus) and do not cause significant damage to cellular ultrastructure. In contrast, photoactivation of such complexes causes severe concentration-dependent damage including membrane-blebbing, vacuolization (cytoplasmic and nuclear), cell shrinkage, organelle damage, and increased cell granularity. This can be attributed to a combination of cell death mechanisms, including autophagy and paraptosis. Inclusion of a recovery period allowed some cells to recover, triggering lipid-mediated repair mechanisms.

Regardless of the photoconditions, platinum was distributed uniformly through cells (including in the cell nucleus), suggesting multiple cellular targets. Notably, irradiation enhanced the accumulation of platinum in cells treated with diazido-Pt drugs, perhaps due to the formation of photolysis products at or close to the cell membranes (which can alter membrane physiology), correlating with a mechanism of action involving radicals. Complementary to this, partial reduction in Pt(IV) to Pt(II) was observed in cells treated with **Pt2** under photoconditions, suggesting that both Pt(II) and Pt(IV) species may be involved in the mechanism of action. In further work, it will be interesting to design a new generation of diazido Pt(IV) prodrugs which remain stable in the dark, but have enhanced ability to undergo photoreduction in cells under visible-light irradiation.

## Supporting Information Available

Materials, instrumentation, methods, supplemental tables and figures (PDF)
3D segmented tomograms of untreated cryopreserved PC3 human prostate cancer cells (AVI)
3D segmented tomograms of Pt-treated cryopreserved PC3 human prostate cancer cells (AVI)

## Supplementary Material

ja1c08630_si_001.pdf

ja1c08630_si_002.avi

ja1c08630_si_003.avi
