# Supplementary material for: Single-Cell Chemistry of Photoactivatable Platinum Anticancer Complexes
Source: J Am Chem Soc. 2021 Nov 22;143(48):20224–40. doi: 10.1021/jacs.1c08630 (PMC8662725; doi:10.1021/jacs.1c08630)
Supplement: Supplementary file 1 — ja1c08630_si_001.pdf [file ja1c08630_si_001.pdf]

Supporting Information

# Single-cell chemistry of photoactivatable platinum anticancer complexes

Elizabeth M. Bolitho, Carlos Sanchez-Cano, Huayun Shi, Paul D. Quinn*, Maria Harkiolaki*, Cinzia Imberti*, Peter J. Sadler*

*Emails:

paul.quinn@diamond.ac.uk

maria.harkiolaki@diamond.ac.uk

cinzia.imberti@warwick.ac.uk

p.j.sadler@warwick.ac.uk

| Contents | |
|---|---|
| **ES1 Materials** | **S4** |
| Chemical reagents | S4 |
| Biological reagents | S4 |
| PC3 (human prostate) carcinoma cells | S4 |
| Synchrotron membranes and grids | S4 |
| **ES2 Instrumentation** | **S4** |
| Inductively coupled Plasma-Optical Emission Spectroscopy (ICP-OES) | S4 |
| Multiplate reader | S4 |
| Plunge-freezer | S4 |
| Freeze-dryer | S4 |
| **ES3 *In vitro* anticancer activity** | **S5** |
| Culture medium | S5 |
| Defrosting PC3 cells | S5 |
| Cell passaging | S5 |
| Sulforhodamine B (SRB) Assay | S5 |
| ICP-OES of drug stock solutions | S5 |
| **Figure S1:** Dose-response curves | |
| **ES4 CryoSIM and CryoSXT** | **S6** |
| Preparation of TEM grids | S6 |
| Fluorescence mapping | S6 |
| Structured Illumination Microscopy(CryoSIM) | S6 |
| **Table S1**: Summary of excitation and emission wavelengths for fluorophores | S6 |
| CryoSIM Data Analysis | S6 |
| CryoSIM Summary | S7 |
| **Figures S2-S4**: CryoSIM images of PC3 cells under dark conditions | S7 |
| **Figures S5-S7**: CryoSIM images of PC3 cells under irradiated conditions | S8 |
| **Figures S8, S9**: CryoSIM images of PC3 treated with **Pt2** under dark conditions | S10 |
| **Figures S10, S11**: CryoSIM images of PC3 treated with **Pt2** under irradiated conditions | S11 |
| Cryo-SXT sample loading | S12 |
| Tomogram reconstructions in IMOD | S12 |
| Summary of tomograms | S12 |
| **Table S2**: Summary of tomograms | S13 |
| **Figure S12**: Cryo-SXT images of PC3 cells under dark conditions | S14 |
| **Figure S13**: Cryo-SXT images of PC3 cells under irradiated conditions | S15 |
| **Figures S14, 15**: Cryo-SXT images of PC3 cells treated with 1× photoIC$_{50}$ of **Pt2** under dark conditions | S16 |
| **Figures S16, 17**: Cryo-SXT images of PC3 cells treated with 0.25× photoIC$_{50}$ of **Pt2** under irradiated conditions | S18 |
| **Figures S18, 19**: Cryo-SXT images of PC3 cells treated with 0.5× photoIC$_{50}$ of **Pt2** under irradiated conditions | S19 |
| **Figures S20, 21**: Cryo-SXT images of PC3 cells treated with 1× photoIC$_{50}$ of **Pt2** under irradiated conditions | S20 |
| **Figures S22, 23**: Cryo-SXT images of PC3 cells treated with 1× photoIC$_{50}$ of **Pt2** under irradiated conditions with the inclusion of a recovery period | S22 |
| 3D segmentation and visualization | S23 |
| 3D segmentation summary | S23 |

| | |
|---|---|
| **Table S3**: Mitochondrial volumes (µm$^3$) | S24 |
| **Table S4**: Lipid droplet volumes (µm$^3$) | S25 |
| **Table S5**: Mean mitochondrial and lipid droplet volumes (µm$^3$) | S26 |
| **Table S6**: Endosomal volumes (µm$^3$) in PC3 cells treated with **Pt2** under dark conditions | S27 |
| **ES5 Synchrotron-XRF** | **S27** |
| Preparation of silicon nitride membranes | S27 |
| I14 sample loading | S27 |
| I14 beamline settings | S28 |
| Data analysis | S28 |
| **Figure S24**: XRF fitting | S28 |
| Elemental mapping summary | S29 |
| **Figures S25-27**: XRF maps of PC3 cells under irradiated conditions | S29 |
| **Figures S28-30**: XRF maps of PC3 cells treated with cisplatin under irradiated conditions | S31 |
| **Figures S31-33**: XRF maps of PC3 cells treated with 5× photoIC$_{50}$ of **Pt1** under dark conditions | S32 |
| **Figures S34-36**: XRF maps of PC3 cells treated with 5× photoIC$_{50}$ of **Pt1** under irradiated conditions | S34 |
| **Figures S37-39**: XRF maps of PC3 cells treated with 5× photoIC$_{50}$ of **Pt2** under dark conditions | S35 |
| **Figures S40-42**: XRF maps of PC3 cells treated with 5× photoIC$_{50}$ of **Pt2** under irradiated conditions | S37 |
| Distribution of Pt in cells | S38 |
| Cell morphology | S38 |
| Area and roundness factors | S39 |
| **Table S7**: Cell area (µm$^2$) and roundness factors (RF) | S39 |
| **Table S8**: Areas of cell nuclei (µm$^2$) | S40 |
| **Table S9**: Intracellular quantities of Pt | S40 |
| Elemental co-localization | S41 |
| **Table S10**: Co-localization between Pt and Zn | S41 |
| **ES6 XANES** | **S42** |
| Preparation of $Si_3N_4$ membranes | S42 |
| Preparation of solid pellets | S42 |
| Analysis of platinum standards | S42 |
| Platinum standards summary | S42 |
| Validation of compressed-sensing method | S42 |
| Extraction of XANES data | S43 |
| Normalization of XANES spectra | S43 |
| Linear Combination Fitting (LCF) analysis of **Pt1** treated cells | S43 |
| **Table S11**: LCF analysis of intracellular Pt(IV) in 5×5 µm$^2$ regions of PC3 cells treated 5× photoIC$_{50}$ of **Pt1** under irradiated conditions | S43 |
| Peak height normalization | S44 |
| XANES of **Pt2** treated cells | S44 |
| **Figure S43**: Normalized XANES spectra of intracellular Pt in two independent 15×15 µm$^2$ regions of PC3 cells treated with 5× photoIC$_{50}$ of **Pt2** under dark conditions | S45 |
| **Figure S44**: Normalized XANES spectra of intracellular Pt in two independent 15×15 µm$^2$ regions of PC3 cells treated with 5× photoIC$_{50}$ of **Pt2** under irradiated conditions | S45 |
| Linear Combination Fitting (LCF) analysis of **Pt2** treated cells | S46 |
| **Table S12**: LCF analysis of intracellular Pt(IV) in 15×15 µm$^2$ regions of PC3 cells treated 5× photoIC$_{50}$ of **Pt2** under dark and irradiated conditions | S46 |
| **Figure S45**. Calibration curve of Pt standards against normalized peak height ratio (a/b) | S47 |
| **ES7 References** | **S47** |

## ES1 Materials

**Chemical reagents.** Platinum (1001±12 mg/L TraceCERT® platinum in 5% *v*/*v* hydrochloric acid) for ICP trace analysis was purchased from Inorganic Ventures and stored at 276 K. All commercial solvents were purchased from Sigma Aldrich. **Pt1** and **Pt2** were synthesized and recrystallized according to published procedures to achieve > 95% purity.[1,2]

**Biological reagents.** Poly-L-lysine solution (0.01%, sterile-filtered, BioReagent, CAS# 25988-63-0), Roswell Park Memorial Institute (RPMI-1640, non-phenol red) and Sulforhodamine B were purchased from Sigma Aldrich. Hank's Balanced Salt Solution (HBSS) was purchased from VMR (Biochrom). Dulbecco's Modified Eagle Medium (DMEM), Phosphate Buffered Saline (PBS), trichloroacetic Acid (TCA), LysoTracker Red DND-99 ($\lambda_{ex/em}$ = 577/590 nm), MitoTracker Deep Red FM ($\lambda_{ex/em}$ = 644/665 nm) were purchased from Fisher Scientific. Gold nanoparticle fiducials (d=250 nm) were purchased from BBI solutions. Heat-inactivated fetal calf serum (FCS), penicillin/streptomycin (penn/strep), L-glutamine and trypsin/EDTA were purchased from PPA Laboratories, and prepared by the Technical Staff at the School of Life Sciences (University of Warwick).

**PC3 (human prostate) carcinoma cells.** PC3 (human prostate) carcinoma cells were purchased from the European Collection of Cell Cultures (ECACC; catalogue number 90112714). Every 6 months, Public Health England confirmed absence of mycoplasma.

**Synchrotron membranes and grids.** Silicon nitride ($Si_3N_4$) membranes were purchased from Silson ltd (frame size = 5 × 5 mm; frame thickness = 200 µm; membrane size = 1.5 × 1.5 mm), membrane = thickness 500 nm, catalogue number SiRN-5.0(0)-200-1.5-500). Quantifoil R2/2 holey carbon films (Au) 200 mesh F1 finder grids were purchased from Quantifoil Micro Tools GmbH (Germany).

## ES2 Instrumentation

**Inductively coupled Plasma-Optical Emission Spectroscopy (ICP-OES*).*** Prior to preparation of synchrotron-XRF samples, stocks of platinum complexes (prepared in 5% *v*/*v* DMSO, 95% *v*/*v* no phenol-red RPMI-1640) were prepared in 3.6% *v*/*v* nitric acid and analyzed on a Perkin Elmer Optima 5300 dv Optical Emission Spectrophotometer (ICP-OES instrument) by monitoring the platinum wavelengths of 265.95 and 204.94 nm. Calibration standards of platinum in the ranging 0-700 ppb were prepared and the salinity of the calibration was adjusted with sodium chloride to match that of the cell culture medium. Data were analyzed on WinLab V3 4.1 software.

**Multiplate reader.** 96-well plates for all SRB assays were analyzed using BioRad iMark microplate reader using the $\lambda$ = 492 nm filter.

**Plunge-freezing**. Carbon-gold TEM grids were plunge-frozen in liquid-nitrogen cooled liquid ethane using a Leica GP2 EM plunge-freezer (available at the B24 beamline). Silicon nitride ($Si_3N_4$) membranes were plunge-frozen in 30% liquid ethane:propane mixture (cooled on liquid nitrogen) using an in-house manufactured manual plunge-freezer at the School of Life Science, University of Warwick.

**Freeze-drying.** Silicon nitride ($Si_3N_4$) membranes were freeze-dried using Alpha 2-4 LDplus Christ model, operating at -85 C temperature and 0.0024 mbar vacuum.

## ES3 *In vitro* anticancer activity

**Culture medium.** Dulbecco's Modified Eagle's Medium (DMEM) was supplemented with 10% fetal calf serum, 1% penicillin/streptomycin and 1% L-glutamine (2 mM) and incubated at 37 ºC prior to use in tissue culture experiments.

**Defrosting PC3 cells.** A frozen ampoule of PC3 (human prostate) cancer cells (*ca.* $1 \times 10^6$ cells in 1 mL) stored in liquid nitrogen was rapidly defrosted at 37 ºC. DMEM (4 mL) was added, pipetted and the cell suspension centrifuged (22 ºC, 1000 rpm, 5 min). The supernatant medium was removed, cells re-suspended in fresh DMEM (4 mL), transferred to a T25 cell culture flask and incubated (37 °C, 5% $CO_2$) until 80-90% surface coverage was achieved.

**Cell passaging.** Upon 80-90% cell surface coverage, the supernatant medium was removed (via suction), cells washed with PBS (3 mL) and 0.25% trypsin/EDTA (1 mL, 5 min, 37 °C). DMEM was added to quench the trypsin activity and pipetted to form a single cell suspension, which was transferred to a T75 cell culture flask for further incubation (37 °C, 5% $CO_2$). This process was repeated upon reaching 80-90% cell surface coverage.

**Sulforhodamine B (SRB) Assay.** A single cell suspension was formed as previously described. Cells were counted (2 × 10 µL) using a haemocytometer and 5000 cells / well in 150 µL were seeded in 96-well F-bottom plates and incubated (48 h, 37 °C, 5% $CO_2$). Stock solutions of **cisplatin**, **Pt1** and **Pt2** were prepared in 5% *v*/*v* DMSO and 95% *v*/*v* non-phenol red RPMI (protected from the light) and diluted to concentrations in the range 0.01-100 µM. Cells were treated with varying concentrations of platinum complex (200 µL/ well) for either: (i) 2 h (under dark conditions) followed by 24 h recovery (drug-free media); (ii) 1 h followed by 1 h irradiation (in drug) with blue light (λ=465 nm, 17 J/cm$^2$) and 24 h recovery (drug-free media). 10% TCA (250 µL) was added to each well (1 h, 4 ºC), plates washed with water (10×), air-dried and 0.4% SRB dye (in 1% acetic acid) was added to each well (30 min). The plates were heat-dried and Tris base (10 mM, pH 10.5) was added to each well (1 h). The plates were analyzed at using a SkanIt multiplate analyser (UV, λ=492 nm). Data were processed in OriginPro Lab 2016.

**ICP-OES.** A calibration of platinum (5% *v*/*v* hydrochloride acid, 1001±12 mg Pt/L TraceCERT®) was prepared in 3.6% *v*/*v* nitric acid in the range 0-700 ppb, and the salinity adjusted with sodium chloride to match that of the RPMI matrix. Prior to synchrotron studies, solutions of **cisplatin**, **Pt1** and **Pt2** (5% DMSO, 95% RMPI-1640) were analyzed by ICP-OES to determine the exact concentration of platinum by monitoring the wavelengths λ=265.95 and 204.94 nm).

v

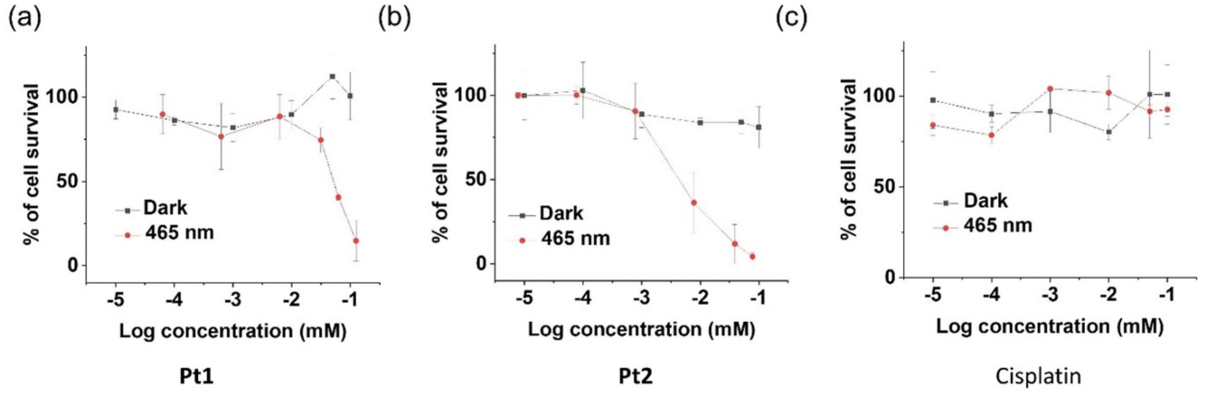


**Figure S1.** Dose-response curve showing percentage cell survival (%) against logarithm of Pt concentration for (a) **Pt1**, (b) **Pt2** and (c) cisplatin in PC3 (human prostate) cancer cells. Cells were treated with varying concentrations of platinum complex (200 μL/ well) for either: (i) 2 h (under dark conditions) followed by 24 h recovery (complex-free media); (ii) 1 h followed by 1 h irradiation (in presence of complex) with blue light (λ=465 nm, 17 J/cm$^2$) and 24 h recovery (drug-free media). The half-maximal inhibitory concentrations ($IC_{50}$ / μM) is defined as the concentration of drug at which 50% of cell growth inhibition is achieved.

## ES4 CryoSIM and CryoSXT

**Preparation of TEM grids.** Quantifoil Au-C R2/2 F1 200 mesh finder grids were incubated in FCS (2 mL/well in 6-well plate) for 12 h (37 °C, 5% $CO_2$). The FCS was removed and 1.5 × 10$^5$ cells/ mL of PC3 cells was added to each well (2 mL/well) for 24 h (37 °C, 5% $CO_2$). A solution of **Pt2** was prepared in non-phenol red RMPI-1640 in dark conditions (protected from the light). The supernatant medium was removed, and cells were treated with 0.25-1× photo$IC_{50}$ of **Pt2** (photo$IC_{50}$=6.48±0.84 μM) for 1 h, followed by irradiation with blue light (1 h, 465 nm, 17 J/cm$^2$) or kept in dark conditions (1 h). The supernatant was removed, and cells recovered in drug-free RMPI-1640 for 0 or 2 h. Grids were then incubated in Mitotracker Deep Red (100 nM) and LysotrackerRed (100 nM) for 20 min (310 K, 5% $CO_2$) before blotting with AuNP fiducials (d= 50 nm, *ca.* 3.5×10$^7$ particles per 10 μL) and plunge-freezing in liquid-nitrogen cooled liquid ethane using a Leica GP2 EM plunge-freezer.

**Fluorescence mapping.** Preliminary correlative microscopy analysis on plunge-frozen grids was performed on a Zeiss Axioimager with a CMS196M LED cryo-correlative stage (Linkam Scientific Ltd) for grid preliminary analysis and mapping (max resolution 50×, variable filters) by monitoring the fluorescence of Mitotracker Deep Red ($\lambda_{ex/em}$=644/665 nm), LysoTracker Red ($\lambda_{ex/em}$=577/590 nm) and blue fluorescence ($\lambda_{ex/em}$=405/450nm) from **Pt2** complex using the mPlum, YFP and DAPI filters, respectively.

**Structural-Illumination Microscopy (cryoSIM).** Prior to cryo-SXT measurements, cryo Structure Illumination Microscopy (super-resolution microscopy) was used to gain fluorescence information on vitrified PC3 cells treated with (i) Mitotracker Deep Red ($\lambda_{ex/em}$=644/665 nm); (ii) Lysotracker Red ($\lambda_{ex/em}$=577/590 nm); (iii) **Pt2** ($\lambda_{ex/em}$=405/450 nm) on Quantifoil grids. This was performed on an in-house developed super-resolution light microscopy facility at Diamond beamline B24,[3] which has a long distance air objective (100X, 2 mm, 0.9NA) with 4 available laser wavelengths (405, 488, 561 and 642 nm) with varying

laser power (10-100 $Wcm^{-2}$), and is coupled to a Linkam cryo-stage. Data were acquired using a DAPI 452 nm reflected filter, red 605 nm transmitted filter and far-red 655 nm transmitted filter to map the mitochondria, lysosomes and blue fluorescence of **Pt2** upon irradiation. A total of 3 maps per quantifoil grid were obtained: (i) z-stack brightfield mosaic; (ii) red (605 nm) + blue (452 nm) overlay; (iii) far-red (655 nm) + blue (452 nm) overlay (**Table S1**).

**Table S1.** Excitation/emission wavelengths of MitroTracker DeepRed, LysoTracker Red and **Pt2** with the excitation laser wavelengths and emission filters available at the B24 beamline cryo-SIM facility.[3]

| Fluorophore | Fluorophore $\lambda_{ex/em}$ (nm) | Excitation wavelength (nm) | Emission filter (nm) |
|---|---|---|---|
| **MitoTracker Deep Red** | 644/665 | 642 | 655 |
| **LysoTracker Red** | 577/590 | 561 | 605 |
| **Pt2** | 405/450 | 405 | 452 |

**CryoSIM Data Analysis.** Data were reconstructed in SoftWoRX 6.5.2 (GE Healthcare) using real optical transfer functions (generated from 3D-SIM images of fluorescent beads of 175 μm) to produce image stacks. Data were then processed in Fiji imaging software to separate the fluorescence channels.[4] The brightness of the blue channel was adjusted to eliminate blue fluorescence in the untreated control cells. The same parameters were then used to separate the channels in cells treated with $1\times IC_{50}$ of **Pt2** under dark and irradiated conditions. For all samples, the blue laser conditions were kept the same ($\lambda$=405 nm, 100 ms, 149.963 mW).

**CryoSIM Summary.** Fluorescently-labelled mitochondria (MitoTracker Deep Red, $\lambda_{ex/em}$=644/665 nm) and lysosomes (Lysotracker Red, $\lambda_{ex/em}$=577/590 nm), were clearly visible in the cytoplasm and enabled identification of the cell nucleus using cryo-SIM, providing information on cell ultrastructure and organelle distribution, Figs S2-S11. However, no blue fluorescent products from photoactivated **Pt2** were observed (perhaps because of quenching).

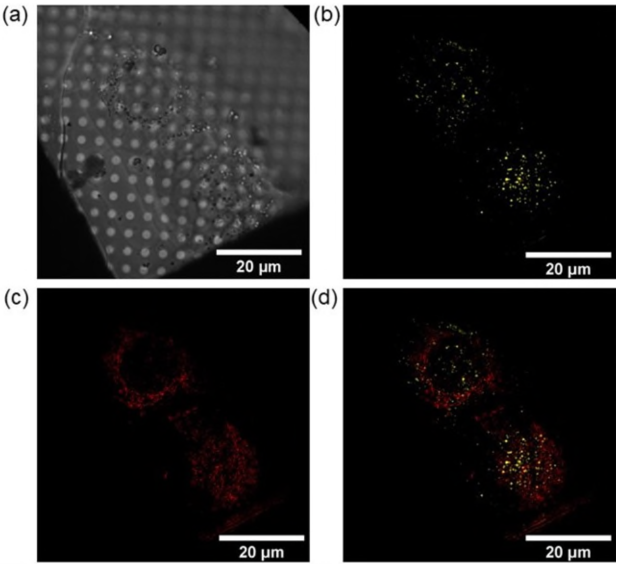


**Figure S2.** Two cryopreserved PC3 human prostate cancer cells grown on carbon-gold TEM grids and exposed to dark conditions (2 h, protected from light) and incubated with MitoTracker Deep Red ($\lambda$=644/665 nm) and Lysotracker Red ($\lambda$=577/590 nm). (a) Brightfield image. (b-d) Super-resolution fluorescence images showing (b) LysoTracker Red (lysosomes), (c) MitoTracker Deep Red (mitochondria) and (d) overlay of LysoTracker Red and MitoTracker Deep Red. Images were generated in Fiji.[4]

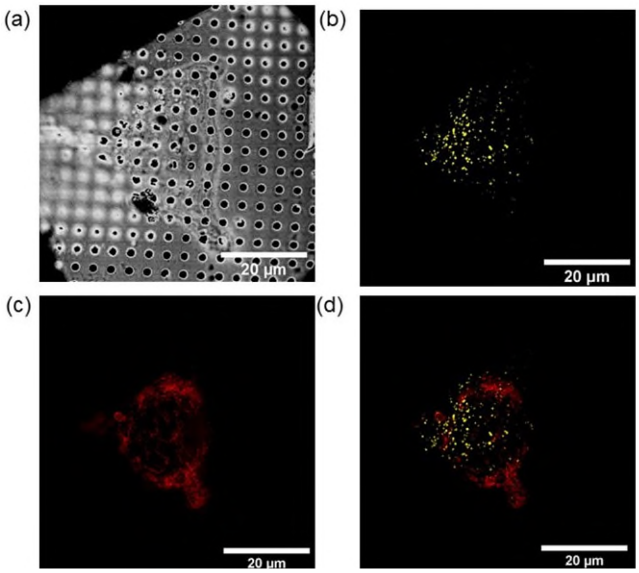


**Figure S3.** A cryopreserved PC3 cell grown on carbon-gold TEM grids and exposed to dark conditions (2 h, protected from light) and incubated with MitoTracker Deep Red (λ=644/665 nm) and Lysotracker Red (λ=577/590nm). (a) Brightfield image. (b-d) Super-resolution fluorescence images showing (b) LysoTracker Red (lysosomes), (c) MitoTracker Deep Red (mitochondria) and (d) overlay of LysoTracker Red and MitoTracker Deep Red. Images were generated in Fiji.[4]

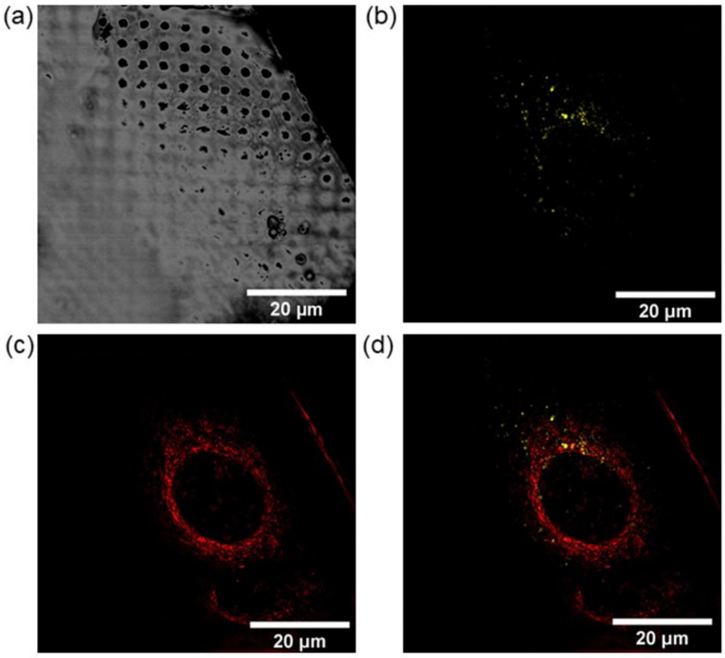


**Figure S4.** A cryopreserved PC3 cell grown on carbon-gold TEM grids and exposed to dark conditions (2 h, protected from light) and incubated with MitoTracker Deep Red (λ=644/665 nm) and Lysotracker Red (λ=577/590nm). (a) Brightfield image. (b-d) Super-resolution fluorescence images showing (b) LysoTracker Red (lysosomes), (c) MitoTracker Deep Red (mitochondria) and (d) overlay of LysoTracker Red and MitoTracker Deep Red. Images were generated in Fiji.[4]

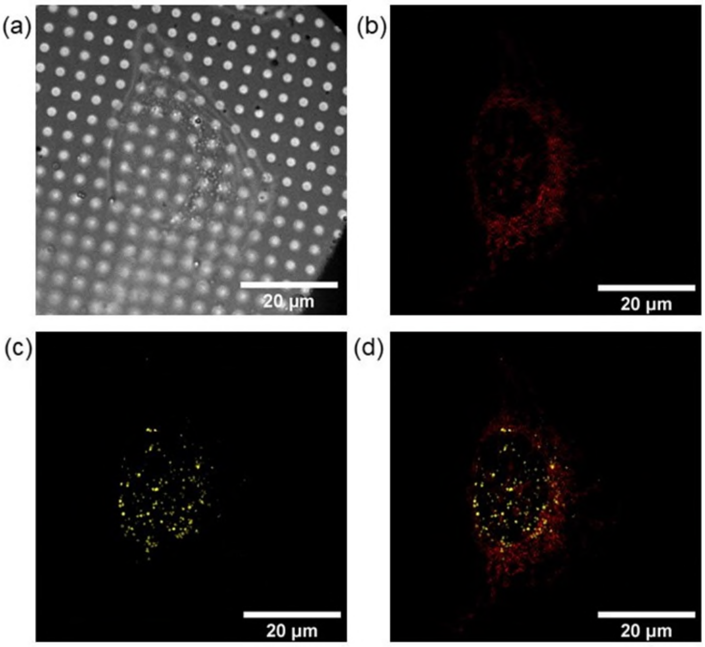


**Figure S5.** A cryopreserved PC3 cell grown on a carbon-gold TEM grids and exposed to dark conditions (1 h) followed by 1 h irradiation with blue light (1 h, 465 nm) and incubated with MitoTracker Deep Red (λ=644/665 nm) and Lysotracker Red (λ=577/590 nm). (a) Brightfield image. (b-d) Super-resolution fluorescence images showing (b) MitoTracker Deep Red (mitochondria), (c) LysoTracker Red (lysosomes) and (d) overlay of LysoTracker Red and MitoTracker Deep Red. Images were generated in Fiji.[4]

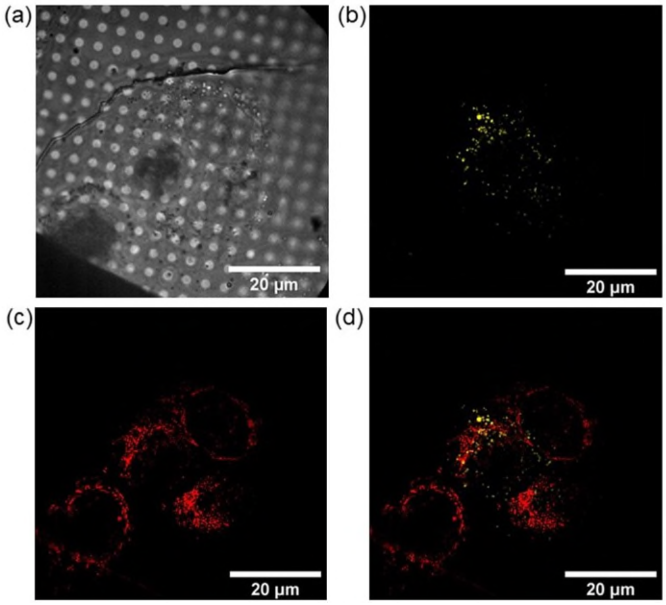


**Figure S6.** Cryopreserved PC3 cells grown on carbon-gold TEM grids and exposed to dark conditions (1 h) followed by 1 h irradiation with blue light (1 h, 465 nm) and incubated with MitoTracker Deep Red (λ=644/665 nm) and Lysotracker Red (λ=577/590nm). (a) Brightfield image. (b-d) Super-resolution fluorescence images showing (b) LysoTracker Red (lysosomes), (c) MitoTracker Deep Red (mitochondria) and (d) overlay of LysoTracker Red and MitoTracker Deep Red. Images were generated in Fiji.[4]

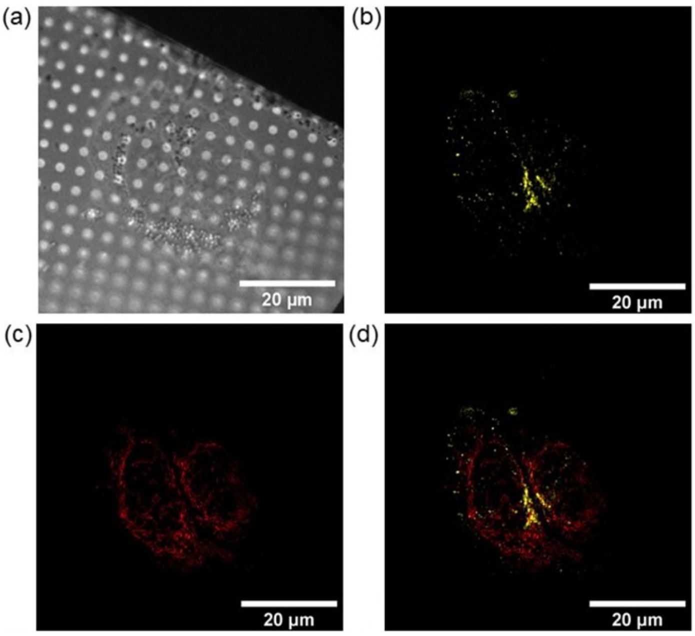


**Figure S7.** Cryopreserved PC3 cell grown on carbon-gold TEM grids and exposed to dark conditions (1 h) followed by 1 h irradiation with blue light (1 h, 465 nm) and incubated with MitoTracker Deep Red (λ=644/665 nm) and Lysotracker Red (λ=577/590 nm). (a) Brightfield image. (b-d) Super-resolution fluorescence images showing (b) LysoTracker Red (lysosomes), (c) MitoTracker Deep Red (mitochondria) and (d) overlay of LysoTracker Red and MitoTracker Deep Red. Images were generated in Fiji.[4]

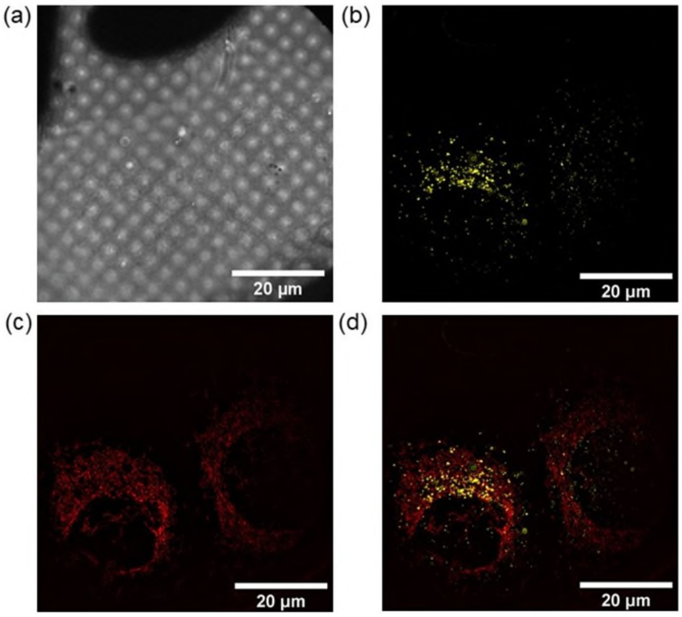


**Figure S8.** Two cryopreserved PC3 cells grown on a carbon-gold TEM grids and exposed 1× photoIC$_{50}$ (6.5 µM) of **Pt2** for 2 h under dark conditions (2 h, protected from light) and incubated with MitoTracker Deep Red (λ=644/665 nm) and Lysotracker Red (λ=577/590 nm). (a) Brightfield image. (b-d) Super-resolution fluorescence images showing (b) LysoTracker Red (lysosomes), (c) MitoTracker DeepRed (mitochondria) and (d) overlay of LysoTracker Red and MitoTracker Deep Red. Images were generated in Fiji.[4]

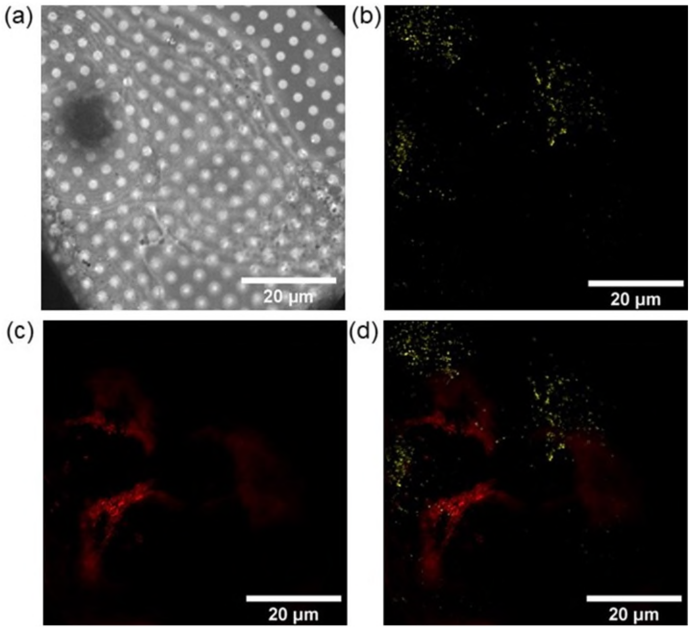


**Figure S9.** Cryopreserved PC3 cells grown on a carbon-gold TEM grids and exposed 1× photo$IC_{50}$ (6.5 µM) of **Pt2** for 2 h under dark conditions (2 h, protected from light) and incubated with MitoTracker Deep Red (λ=644/665 nm) and Lysotracker Red (λ=577/590 nm). (a) Brightfield image. (b-d) Super-resolution fluorescence images showing (b) LysoTracker Red (lysosomes), (c) MitoTracker Deep Red (mitochondria) and (d) overlay of LysoTracker Red and MitoTracker Deep Red. Images were generated in Fiji.[4]

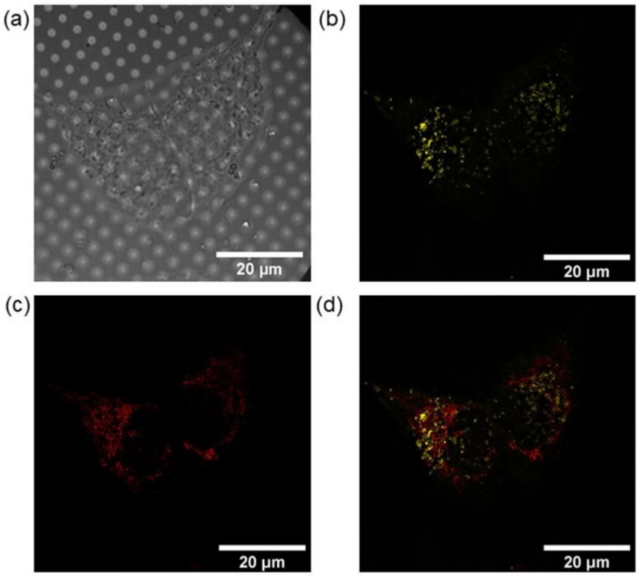


**Figure S10.** Two cryopreserved PC3 cells grown on a carbon-gold TEM grids and exposed 1× photo$IC_{50}$ (6.5 µM) of **Pt2** dark conditions (1 h) followed by 1 h irradiation with blue light conditions (1 h, 465 nm) and incubated with MitoTracker Deep Red (λ=644/665 nm) and Lysotracker Red (λ=577/590 nm). (a) Brightfield image. (b-d) Super-resolution fluorescence images showing (b) LysoTracker Red (lysosomes), (c) MitoTracker Deep Red (mitochondria) and (d) overlay of LysoTracker Red and MitoTracker Deep Red. Images were generated in Fiji.[4] Possible blue fluorescence of coumarin radicals ($\lambda_{em}$= 440 nm) was not observed.

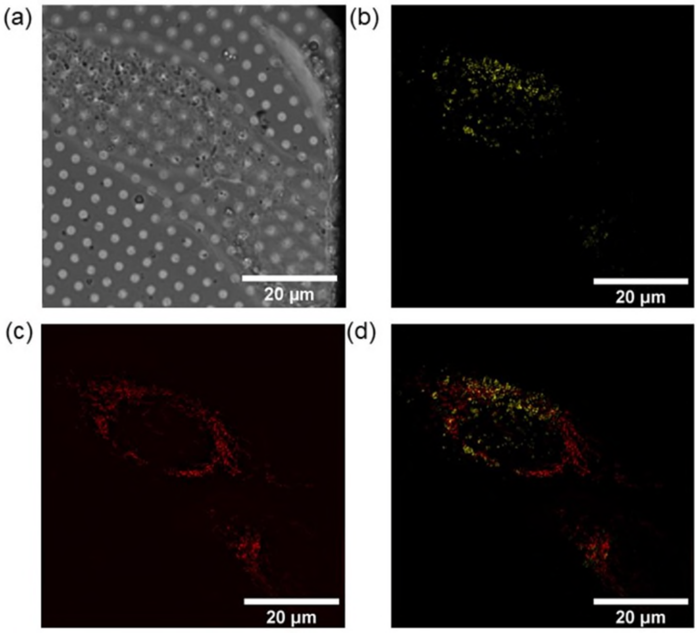


**Figure S11.** Two cryopreserved PC3 cells grown on a carbon-gold TEM grids and exposed 1× photoIC$_{50}$ (6.5 µM) of **Pt2** dark conditions (1 h) followed by 1 h irradiation with blue light conditions (1 h, 465 nm) and incubated with MitoTracker Deep Red (λ=644/665 nm) and Lysotracker Red (λ=577/590 nm). (a) Brightfield image. (b-d) Super-resolution fluorescence images showing (b) LysoTracker Red (lysosomes), (c) MitoTracker Deep Red (mitochondria) and (d) overlay of LysoTracker Red and MitoTracker Deep Red. Images were generated in Fiji.[4] Possible blue fluorescence of coumarin radicals ($\lambda_{em}$= 440 nm) was not observed.

**Cryo-SXT sample loading.** The pre-mapped quantifoil grids of interest were loaded into 4 sample holders at the B24 beamline under cryogenic conditions (-163 °C) and brightfield mosaic images were obtained for each individual grid using short X-ray exposures (0.5 s, 500 eV) and detected with a CCD (Pixis XO 1024B Princeton Scientific). From this, the positions of cells of interest were selected, and mosaics of individual regions were obtained. The X-ray camera (Pixis-XO 1024B) was then used in continuous acquisition mode to obtain detailed information on individual cells, and square regions (15.8×15.8 µm$^2$) of regions of interest were obtained. The view was brought into focus by using the positions of lipid droplets at -30 ° and +30 ° tilt angles. Once focussed, the tilt limit was identified by rotating to no more than -70 ° and +70 °, respectively. The cell depth of interest was adjusted by altering the position of the 40 nm zone plate objective. The cells of interest were then analyzed by cryo-SXT using 1 s exposure and 0.5 ° steps, unless specified otherwise.

**Tomogram reconstructions in IMOD.** Tomograms were aligned and reconstructed automatically (unless specified otherwise) using a processing pipeline developed by Diamond Light Source, using Batchruntomo SIRT and WBP algorithms. Tomograms which were not reconstructed sufficiently were reconstructed manually in IMOD software[5] using the following settings: axis type = single axis, frame type = single frame, pixel size = 15.8 nm, fiducial (gold nanoparticle) diameter = 250 nm, image rotation = 0 degrees, starting angle = -65°, increment step = 0.2-0.5°. An X-ray model was generated, the cross-correlation calculated (using high frequency cut-off radius of 0.1) which generated a coarse aligned stack, for which a fiducial model was constructed. Fiducials were manually selected for the tilt series and fine-aligned relative to the neighbouring views (no rotation and fixed magnification of 1). The tomogram was positioned (thickness = 1000 and binning = 3) and a boundary model generated (to remove

unwanted information from the tomogram). The final, reconstructed tomogram was generated in IMOD imaging software[5] and processed (to remove any noise) and exported as .rec files for volume segmentation analysis using SuRVoS.[6]

**Summary of tomograms**. A total of 19 X-ray tomograms (**Tomograms T1-19**, **T1-19** in Figs S12-S23) of 15.8 × 15.8 µm$^2$ areas of PC3 cells treated with coumarin complex **Pt2** under various conditions were acquired at a resolution of 40 nm (Table S2): (i) Untreated dark controls (Fig. S12, **T1-3**), (ii) Untreated irradiated (1 h, 465 nm, 17 J/cm$^2$) controls (Fig. S13, **T4-6**), (iii) 1× photoIC$_{50}$ (6.5 µM) of **Pt2** exposed to 2 h dark conditions (Figs S14, S15, **T7-9**), (iv) 0.25× photoIC$_{50}$ (1.6 µM) of **Pt2** for 1 h, followed by 1 h irradiation (in-drug) with blue 465 nm light (17 J/cm$^2$) (Figs S16, S17, **T10-12**), (v) 0.5× photoIC$_{50}$ (3.2 µM) of **Pt2** for 1 h, followed by 1 h irradiation (in-drug) with 465 nm light (17 J/cm$^2$), Figs S18, S19, **T13,14**), (vi) 1× photoIC$_{50}$ (6.5 µM) of **Pt2** for 1 h, followed by 1 h irradiation (in-drug) with 465 nm (17 J/cm$^2$) light (Figs S20, S21, **T15-17**), (vii) 1× photoIC$_{50}$ (6.5 µM) of **Pt2** for 1 h, followed by 1 h irradiation (in-drug) with 465 nm (17 J/cm$^2$) light and 2 h recovery in drug-free media (Figs S22, S23, **T18,19**).

**Table S2.** Summary of the tomograms of cryopreserved PC3 (human prostate) cancer cells treated with: (i) 1× photoIC$_{50}$ of **Pt2** (photoIC$_{50}$=6.48 ± 0.84 µM) for 2 h under dark conditions, (ii) 0.25-1× photoIC$_{50}$ (1.6-6.5 µM) of **Pt2** for 1 h, followed by 1 h irradiation (in-drug) with blue light (465 nm, 17 J/cm$^2$), (iii) 1× photoIC$_{50}$ of **Pt2** 1 h, followed by 1 h irradiation (in-drug) with blue light (465 nm, 17 J/cm$^2$) and 2 h recovery in drug-free media.

| Conditions | Tomo | Rotation steps (°) | Tilt range (°) | SI Figure | Video number |
|---|---|---|---|---|---|
| **Untreated control (dark)** | **T1** | 0.5 | -70 to +70 | **S12** | **Video_T1** |
| | **T2** | 0.2 | -65 to +65 | **S12** | **Video_T2** |
| | **T3** | 0.2 | -70 to +70 | **S12** | **Video_T3** |
| **Untreated control (465 nm)** | **T4** | 0.5 | -70 to +60 | **S13** | **Video_T4** |
| | **T5** | 0.2 | -65 to +65 | **S13** | **Video_T5** |
| | **T6** | 0.5 | -65 to +65 | **S13** | **Video_T6** |
| **1× photoIC$_{50}$ (dark)** | **T7** | 0.2 | -60 to +60 | **S14** | **Video_T7** |
| | **T8** | 0.5 | -65 to +65 | **S15** | **Video_T8** |
| | **T9** | 0.5 | -65 to +65 | **S15** | **Video_T9** |
| **0.25× photoIC$_{50}$ (465 nm)** | **T10** | 0.5 | -65 to +65 | **S16** | **Video_T10** |
| | **T11** | 0.5 | -65 to +65 | **S16** | **Video_T11** |
| | **T12** | 0.2 | -65 to +70 | **S17** | **Video_T12** |
| **0.5× photoIC$_{50}$ (465 nm)** | **T13** | 0.5 | -65 to +70 | **S18** | **Video_T13** |
| | **T14** | 0.2 | -65 to +50 | **S19** | **Video_T14** |
| **1× photoIC$_{50}$ (465 nm)** | **T15** | 0.5 | -55 to +60 | **S20** | **Video_T15** |
| | **T16** | 0.2 | -60 to +60 | **S21** | **Video_T16** |
| | **T17** | 0.2 | -65 to +65 | **S21** | **Video_T17** |
| **1× photoIC$_{50}$ (465 nm + 2 h) [illegible]** | **T18** | 0.5 | -55 to +55 | **S22** | **Video_T18** |
| | **T19** | 0.2 | -65 to +65 | **S23** | **Video_T19** |

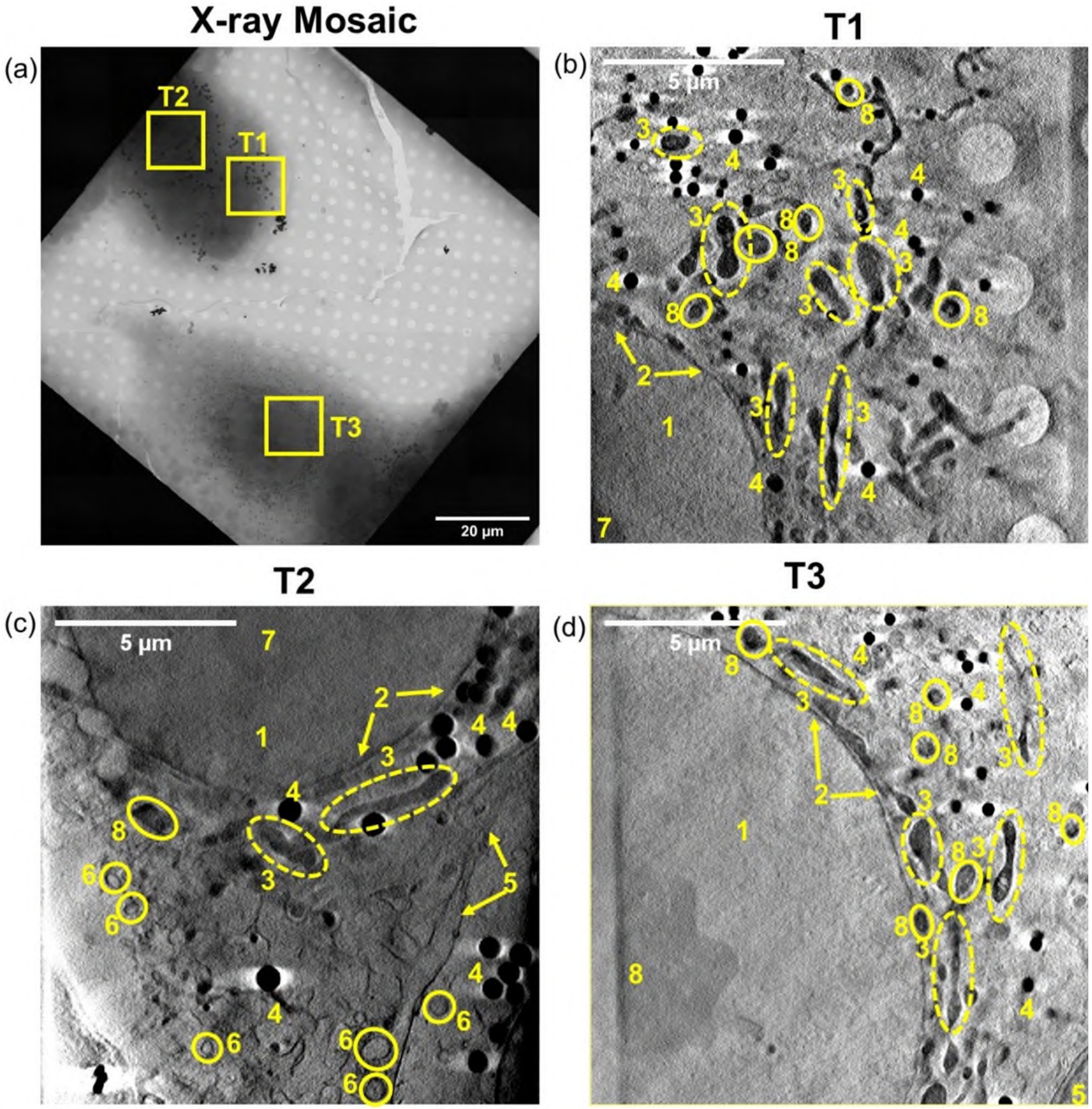


**Figure S12.** (a) X-ray mosaic of cryopreserved PC3 cells grown on Quantifoil TEM grids, exposed to dark conditions for 2 h, and the areas of interest (15.8×15.8 $\mu m^2$) mapped using cryo-SXT; (b-d) reconstructed tomograms (**T1-3, Videos_T1, T2, T3**) showing cellular features: (1) nucleus; (2) nuclear membrane; (3) mitochondria; (4) lipid droplets; (5) plasma membrane; (6) vesicles; (7) nucleolus; (8) dense organelles; (9) endosomes. Images were generated in IMOD software.[5]

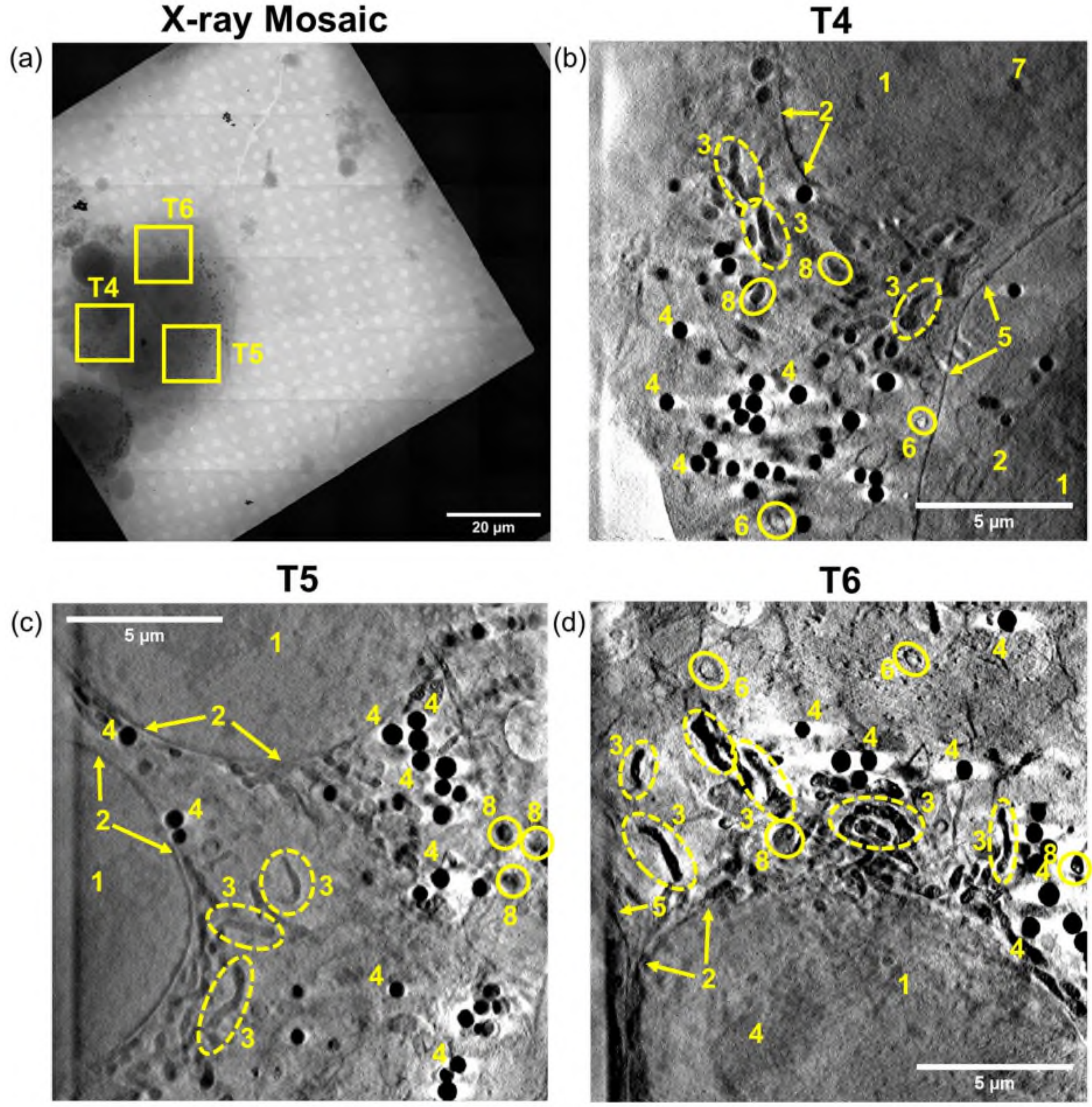


**Figure S13.** (a) X-ray mosaic of cryopreserved PC3 cells grown on Quantifoil TEM grids, exposed to 1 h irradiation (blue light, λ=465 nm) and the areas of interest (15.8×15.8 $\mu m^2$) mapped using cryo-SXT. (b-d) reconstructed tomograms (**T4-6**, **Videos_T4**, **T5**, **T6**) showing cellular features: (1) nucleus; (2) nuclear membrane; (3) mitochondria; (4) lipid droplets; (5) plasma membrane; (6) vesicles; (7) nucleolus; (8) dense organelles; (9) endosomes. Images were generated in IMOD software.[5]

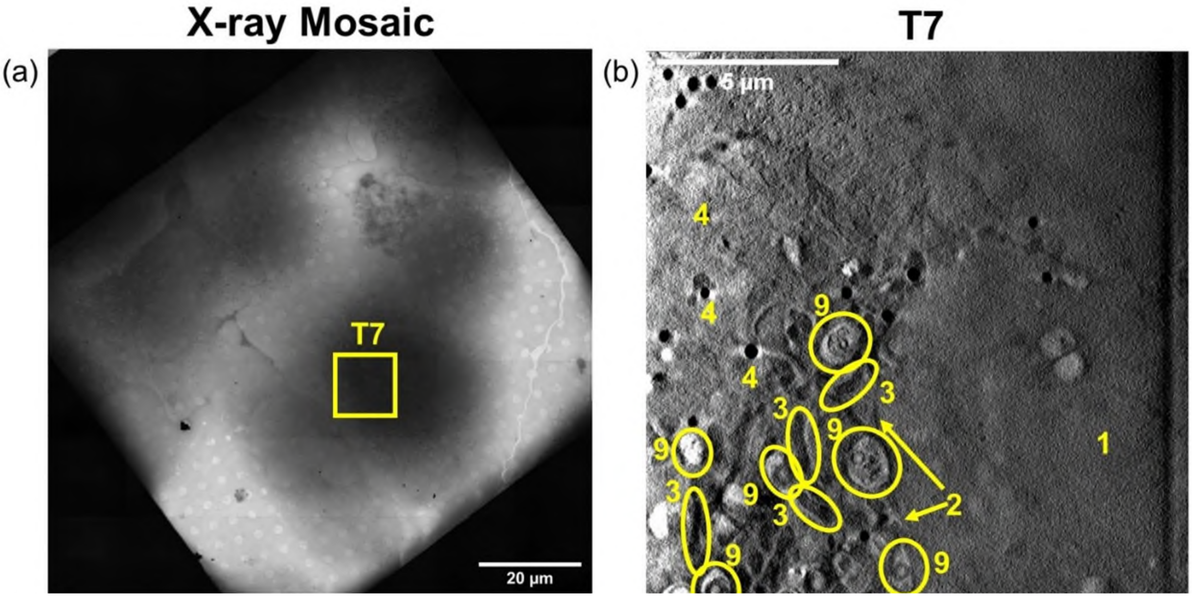


**Figure S14.** (a) X-ray mosaic of cryopreserved PC3 cells grown on Quantifoil TEM grids treated with 1× irradiated $IC_{50}$ (6.5 µM) of **Pt2** (2 h, dark conditions) and the areas of interest (15.8×15.8 $\mu m^2$) mapped using cryo-SXT; (b) reconstructed tomogram (**T7, Video_T7**) showing cellular features: (1) nucleus; (2) nuclear membrane; (3) mitochondria; (4) lipid droplets; (5) plasma membrane; (6) vesicles; (7) nucleolus; (8) dense organelles; (9) endosomes. Images were generated in IMOD software.[5]

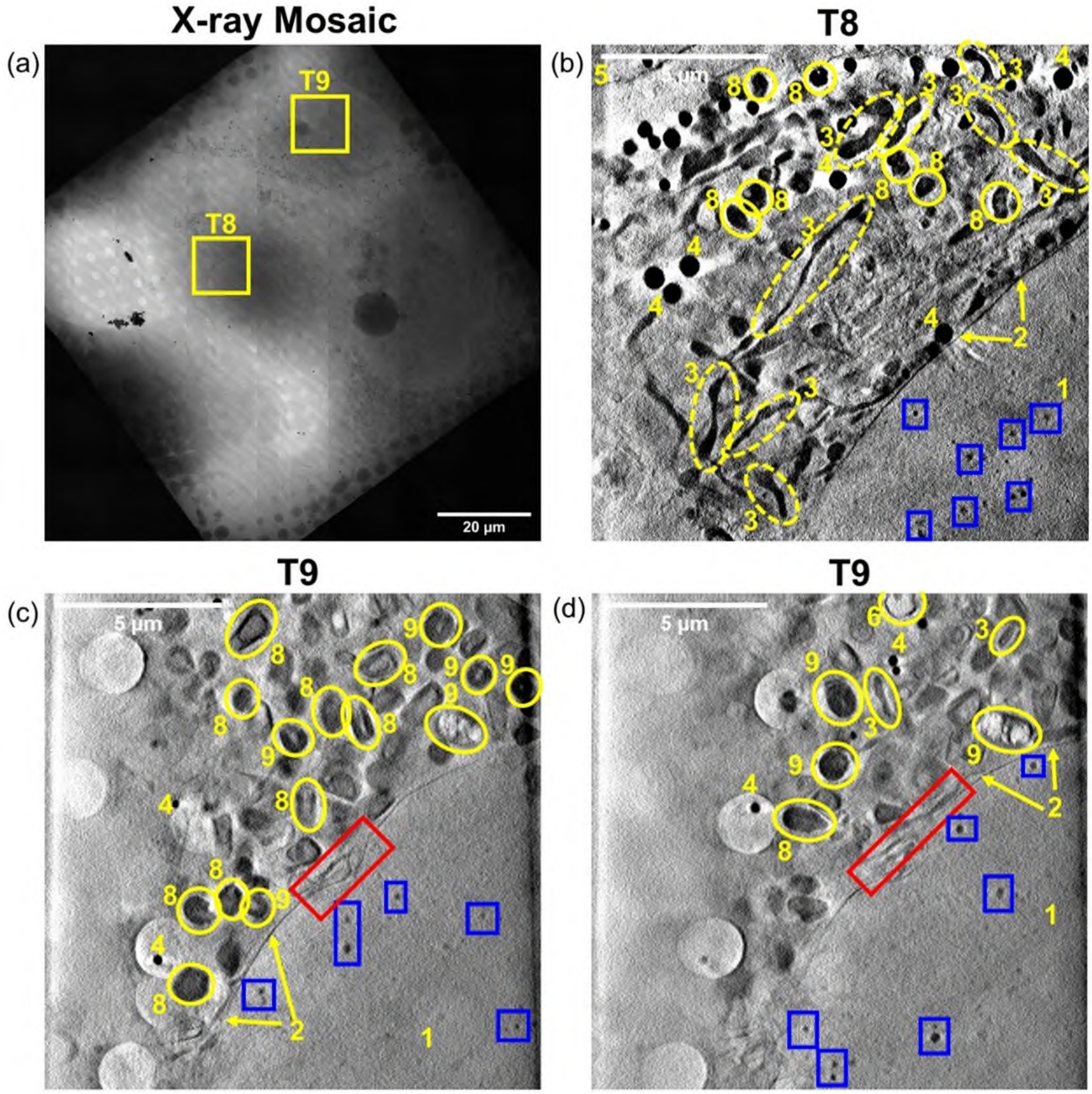


**Figure S15.** (a) X-ray mosaic of cryopreserved PC3 cells grown on Quantifoil TEM grids treated with 1× photo$IC_{50}$ (6.5 μM) of **Pt2** (2 h, dark conditions) and the areas of interest (15.8×15.8 $μm^2$) mapped using cryo-SXT. (b-d) reconstructed tomograms (**T8-9, Videos_T8, T9**) showing cellular features: (1) nucleus; (2) nuclear membrane; (3) mitochondria; (4) lipid droplets; (5) plasma membrane; (6) vesicles; (7) nucleolus; (8) dense organelles; (9) endosomes. Tiny black spots (high X-ray absorption) were observed in the nucleus of two independent PC3 cells treated with 6.5 μM of **Pt2** under dark conditions as represented in the blue boxed regions. Nuclear membrane invaginations were also observed (identified by the red box), a typical feature of cancer cells.[7] Images were generated in IMOD software.[5]

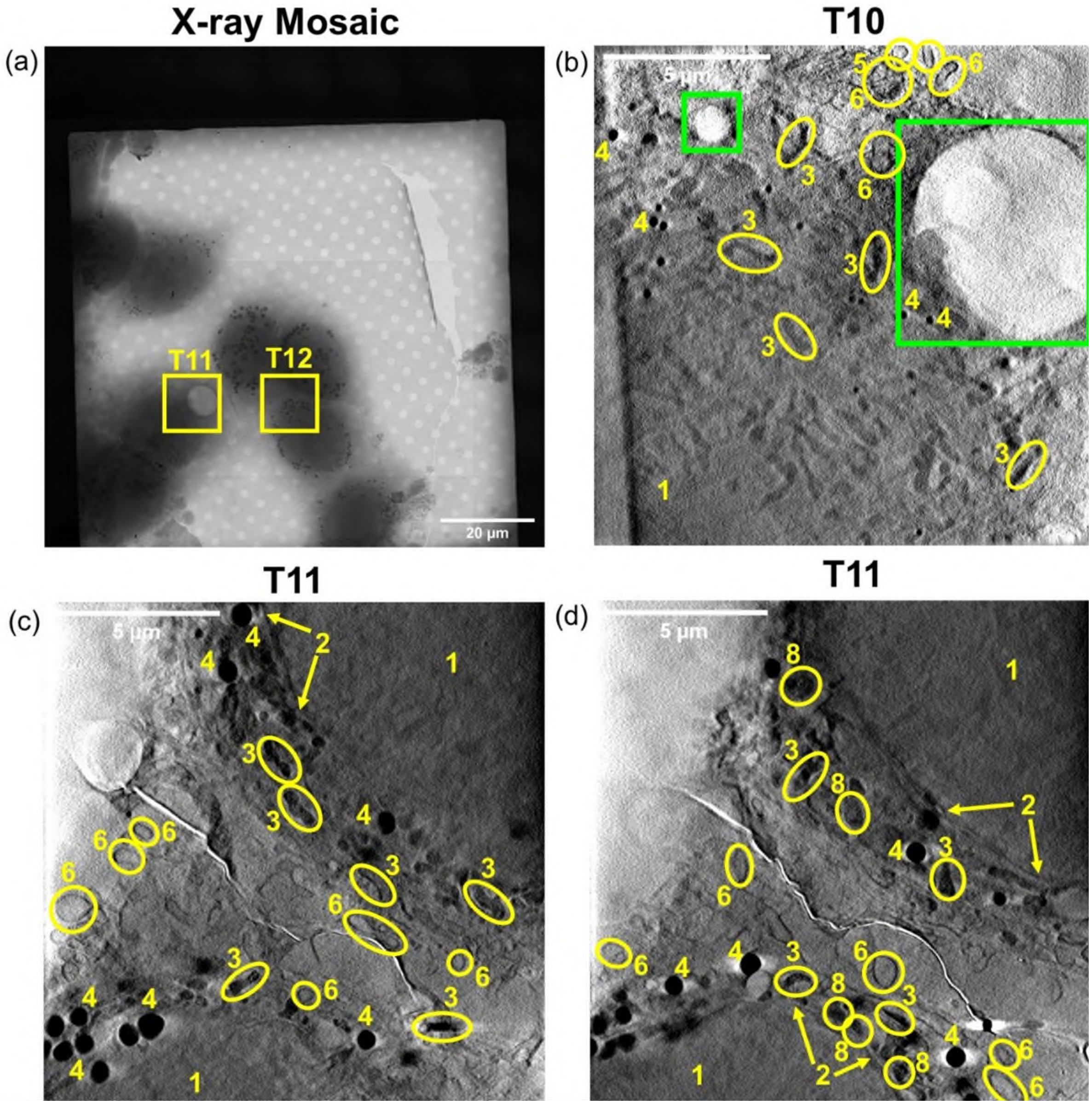


**Figure S16.** (a) X-ray mosaic of cryopreserved PC3 cells grown on Quantifoil TEM grids treated with 0.25×photoIC$_{50}$ (1.6 µM) **Pt2** for 1 h followed by 1 h irradiation (465 nm), with the areas of interest (15.8×15.8 µm$^2$) mapped using cryo-SXT. (b-d) reconstructed tomograms (**T10-11**, **Videos_T10**, **T11**) showing cellular features: (1) nucleus; (2) nuclear membrane; (3) mitochondria; (4) lipid droplets; (5) plasma membrane; (6) vesicles; (7) nucleolus; (8) dense organelles; (9) endosomes. A large cytoplasmic vacuole can be observed in **T10** (green outline), a common feature of programmed cell death.[8] Images were generated in IMOD software.[5]

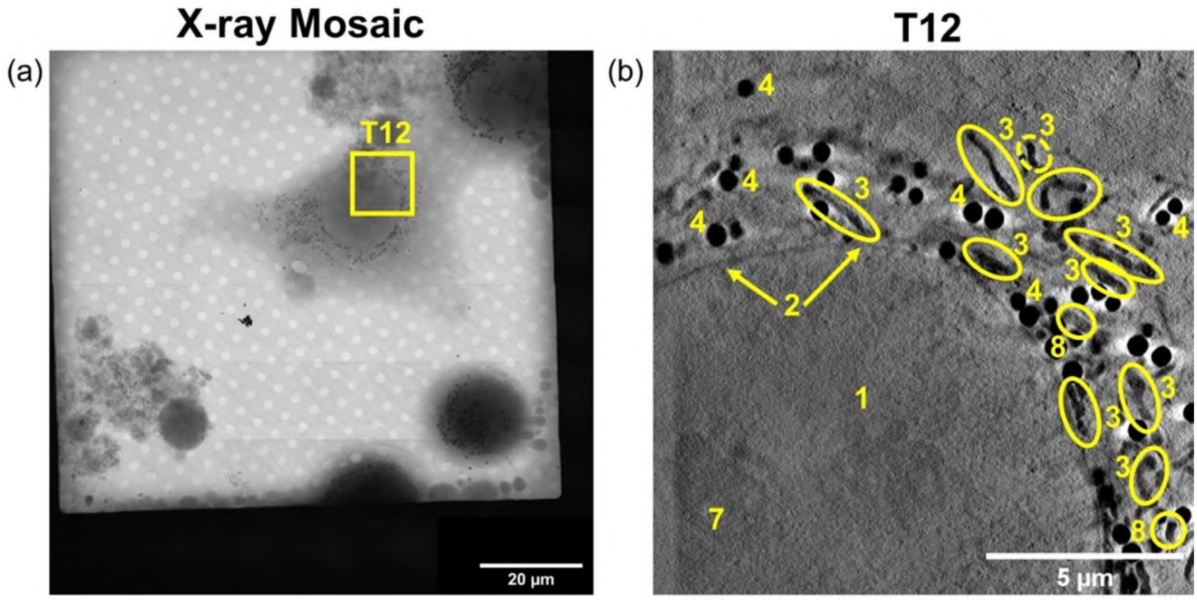


**Figure S17.** (a) X-ray mosaic of a cryopreserved PC3 cell grown on Quantifoil TEM grids treated with 0.25×photo$IC_{50}$ (1.6 µM) of **Pt2** for 1 h followed by 1 h irradiation (465 nm), with the areas of interest (15.8×15.8 $µm^2$) mapped using cryo-SXT; (b) reconstructed tomogram (**T12, Video_T12**) showing cellular features: (1) nucleus; (2) nuclear membrane; (3) mitochondria; (4) lipid droplets; (5) plasma membrane; (6) vesicles; (7) nucleolus; (8) dense organelles; (9) endosomes. Images were generated in IMOD software.[5]

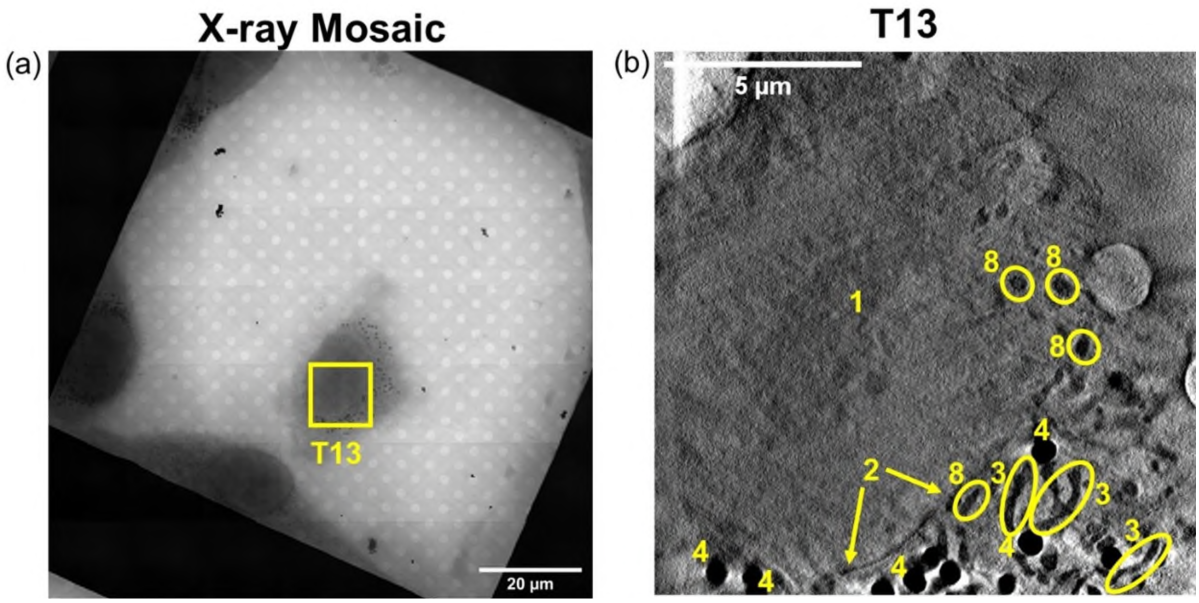


**Figure S18.** (a) X-ray mosaic of a cryopreserved PC3 cell grown on Quantifoil TEM grids treated with 0.5×photo$IC_{50}$ (3 µM) of **Pt2** for 1 h followed by 1 h irradiation (465 nm), with the areas of interest (15.8×15.8 $µm^2$) mapped using cryo-SXT. (b) Reconstructed tomogram (**T13, Video_T13)** showing cellular features: (1) nucleus; (2) nuclear membrane; (3) mitochondria; (4) lipid droplets; (5) plasma membrane; (6) vesicles; (7) nucleolus; (8) dense organelles; (9) endosomes. Images were generated in IMOD software.[5]

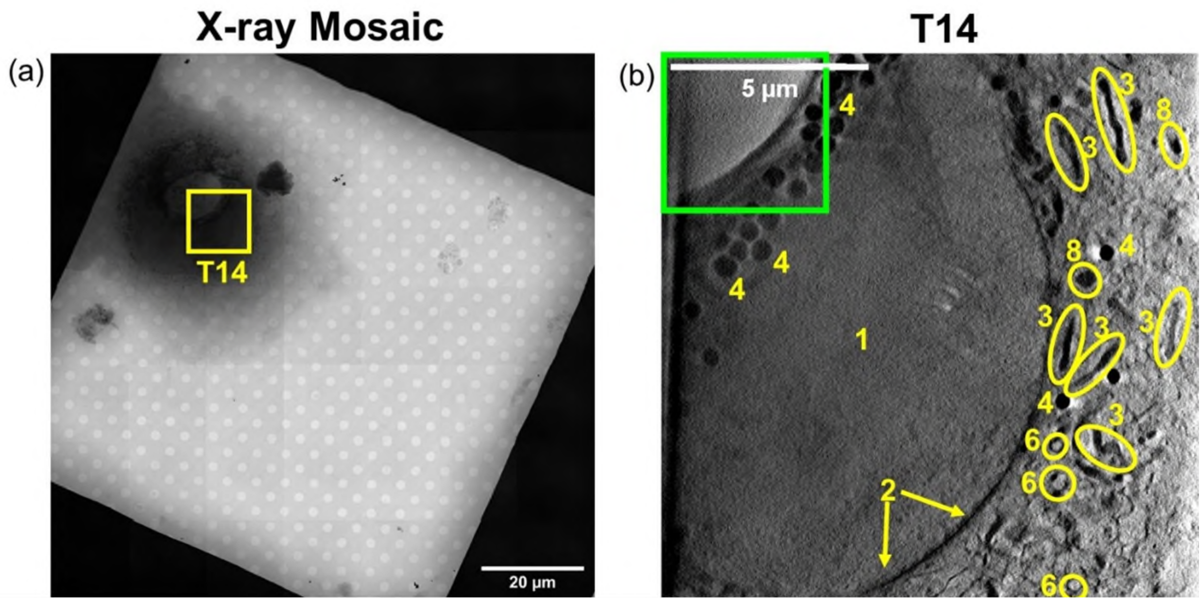


**Figure S19.** (a) X-ray mosaic of a cryopreserved PC3 cell grown on Quantifoil TEM grids treated with 0.5×photoIC$_{50}$ (3 µM) of **Pt2** for 1 h followed by 1 h irradiation (465 nm), with the areas of interest (15.8×15.8 µm$^2$) mapped using cryo-SXT. (b) Reconstructed tomogram (**T14, Video_T14**) showing cellular features: (1) nucleus; (2) nuclear membrane; (3) mitochondria; (4) lipid droplets; (5) plasma membrane; (6) vesicles; (7) nucleolus; (8) dense organelles; (9) endosomes. A large nuclear vacuole is observed (green box). Images were generated in IMOD software.[5]

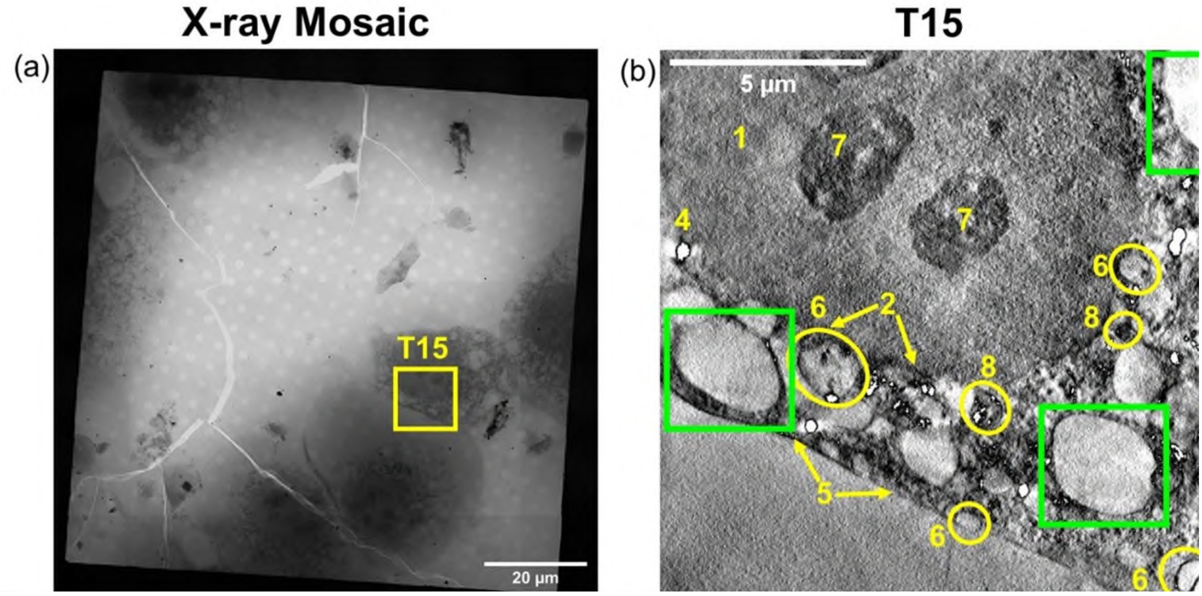


**Figure S20.** (a) X-ray mosaic of three prostate cancer cells grown on Quantifoil TEM grids treated with 1×photoIC$_{50}$ (6.5 µM) of **Pt2** for 1 h followed by 1 h irradiation (465 nm), with the areas of interest (15.8×15.8 µm$^2$) mapped using cryo-SXT. (b) Reconstructed tomogram (**T15, Video_T15**) showing cellular features: (1) nucleus; (2) nuclear membrane; (3) mitochondria; (4) lipid droplets; (5) plasma membrane; (6) vesicles; (7) nucleolus; (8) dense organelles; (9) endosomes. Cytoplasmic vacuoles (green boxes) are observed, which is a common feature of programmed cell death.[8] Images were generated in IMOD software.[5]

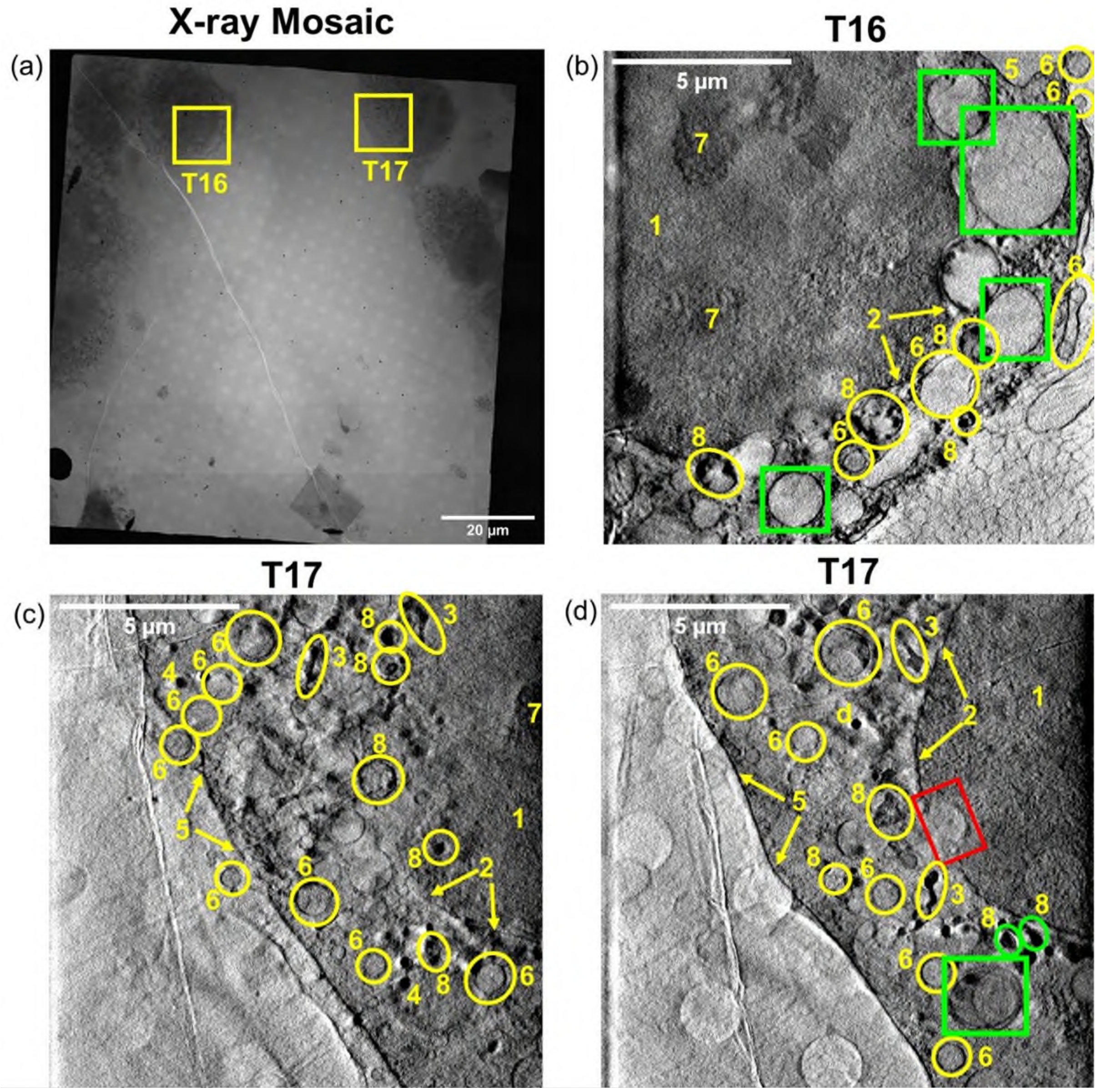


**Figure S21.** (a) X-ray mosaic of two PC3 cells grown on Quantifoil TEM grids treated with 1×photoIC$_{50}$ (6.5 µM) of **Pt2** for 1 h followed by 1 h irradiation (465 nm), with the areas of interest (15.8×15.8 µm$^2$) mapped using cryo-SXT. (b-d) Reconstructed tomograms (**T16-17**, **Videos_T16, T17**) showing cellular features: (1) nucleus; (2) nuclear membrane; (3) mitochondria; (4) lipid droplets; (5) plasma membrane; (6) vesicles; (7) nucleolus; (8) dense organelles; (9) endosomes. Cytoplasmic vacuoles were observed (shown in green), which is a common feature of programmed cell death.[8] Invaginations of the nuclear membrane are present in **T16-17** (shown in red), a common feature of cancer cells.[7] Images were generated in IMOD software.[5]

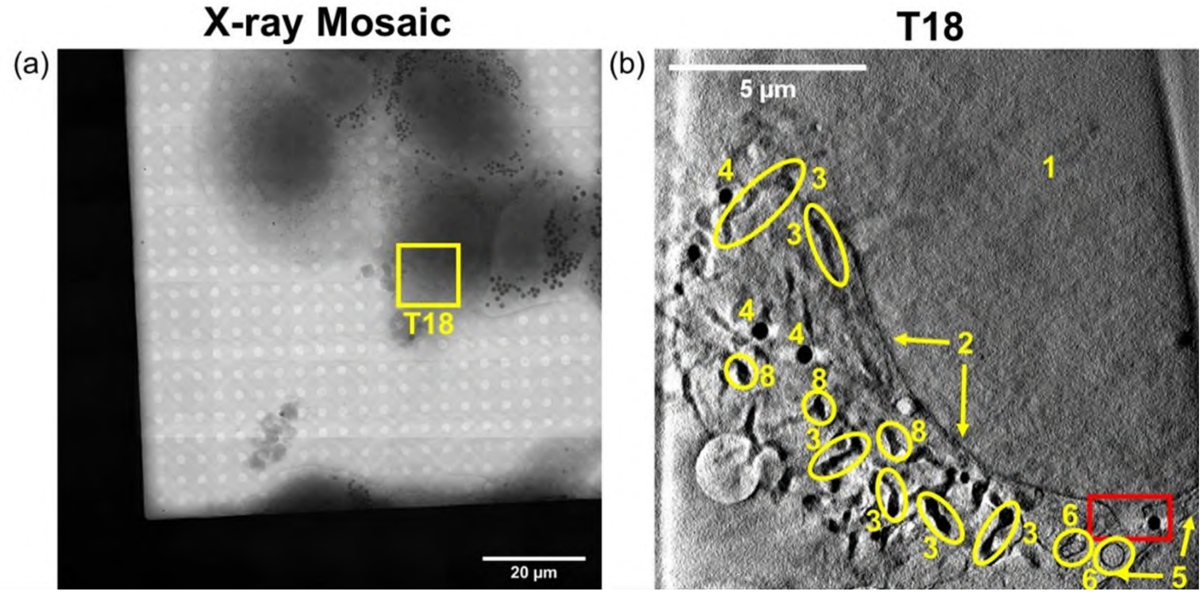


**Figure S22.** (a) X-ray mosaic of prostate cancer cells grown on Quantifoil TEM grids treated with 1×photo$IC_{50}$ (6.5 µM) of **Pt2** for 1 h followed by 1 h irradiation (465 nm) and 2 h recovery in drug-free media, the areas of interest (15.8×15.8 µm$^2$) mapped using cryo X-ray tomography. (b) Reconstructed tomogram (**T18, Video_T18**) showing cellular features: (1) nucleus; (2) nuclear membrane; (3) mitochondria; (4) lipid droplets; (5) plasma membrane; (6) vesicles; (7) nucleolus; (8) dense organelles; (9) endosomes. Invaginations of the nuclear membrane were present (red), a common feature of cancer cells.[7] Images were generated in IMOD software.[5]

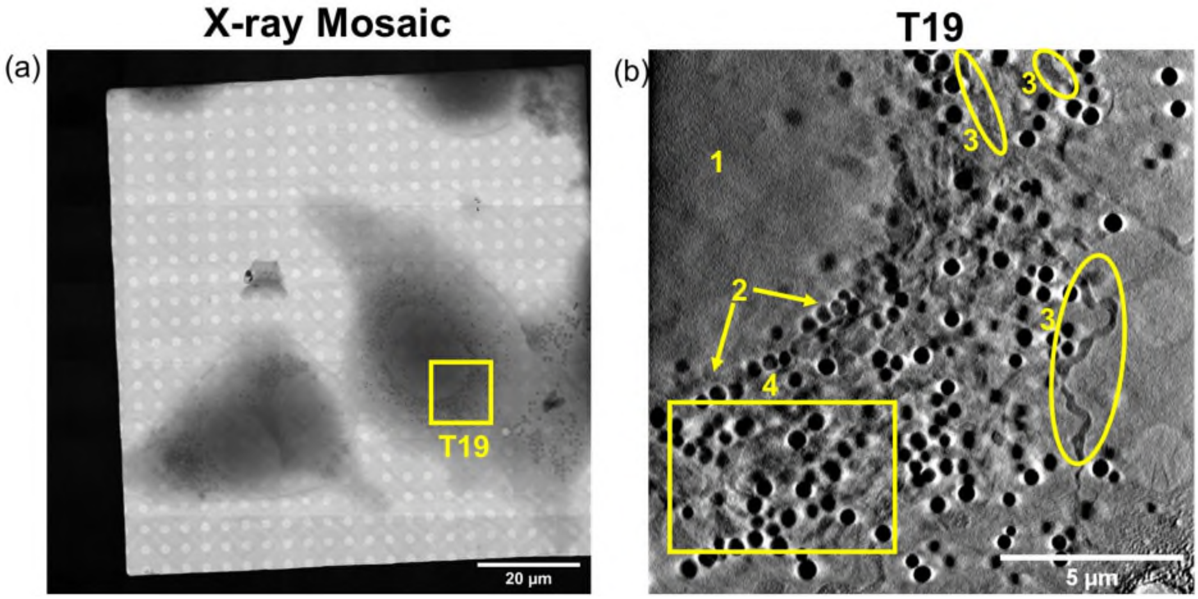


**Figure S23.** (a) X-ray mosaic of prostate cancer cells grown on Quantifoil TEM grids treated with 1× photo$IC_{50}$ (6.5 µM) of **Pt2** for 1 h followed by 1 h irradiation (465 nm) and 2 h recovery in drug-free media, showing the area of interest (15.8×15.8 µm$^2$) mapped using cryo X-ray tomography. (b) Reconstructed tomogram (**T19, Video_T19**) showing cellular features: (1) nucleus; (2) nuclear membrane; (3) mitochondria; (4) lipid droplets; (5) plasma membrane; (6) vesicles; (7) nucleolus; (8) dense organelles; (9) endosomes. Note that this PC3 prostate cancer cell has a significantly enhanced number of lipid droplets (total = 88) compared to the control cells. Images were generated in IMOD software.[5]

**3D segmentation and visualization.** Volume segmentation was performed on reconstructed tomograms (.rec) using SuRVoS[6] software and selecting regions of interest. A Gaussian filter channel was used to visualize the data and supervoxels (SP=6×6×6, compactness=20) were generated. A minimum of 10 mitochondria and 6 lipids from each tomogram (15.8×15.8 $\mu m^2$) were segmented to calculate average organelle sizes. Full segmentation of two tomograms (**T3** and **T16**; **Videos_T20**, **T21**) was performed, showing volumes of (i) nucleus; (ii) nucleoli; (iii) mitochondria; (iv) lipid droplets; (v) plasma membrane; (vi) vacuoles, (vii) dense organelles, (viii) unidentifiable damaged organelles. Segmented tomograms were visualized in Amira.[9]

**3D segmentation summary.** The volumes of mitochondria and lipid droplets ($\mu m^3$) in each tomogram (Tables S3-5) were not statistically significant different between cells treated with 0 to 1× photo$IC_{50}$ (6.5 µM) of **Pt2** under all conditions analyzed. Endosomes were identified in two independent PC3 cells treated with **Pt2** under dark conditions (Figs S13, S14, Table S6), with mean sizes of 0.26±0.13 and 0.33±0.23 $\mu m^3$, respectively. Additionally, small, dark spots observed in the cell nucleus of PC3 cells treated with **Pt2** under dark conditions ranged in volume between 0.002-0.042 and 0.003-0.031 $\mu m^3$ (Fig. S14). Full segmentation of two tomograms (i) untreated control (2 h dark conditions), (ii) 1× photo$IC_{50}$ of **Pt2** (1 h + 1 h blue light irradiation) in Fig. 5. illustrates the extreme morphological differences in 3D.

**Table S3.** Individual and mean mitochondrial volumes for cryopreserved PC3 cells treated with 0–1× photoIC$_{50}$ (0-6.5 µM) of **Pt2** under dark (2 h) or blue light (1 h exposure + 1 h 465 nm irradiation) conditions of tomograms **T1-19** as determined in SuRVoS:[6] (i) **T1-3** Untreated control (dark, 2 h), (ii) **T4-6** Untreated control (465 nm, 1 h), (iii) **T7-9** 1× photoIC$_{50}$ (6.5 µM) of **Pt2** (2 h dark), (iv) **T10-12** 0.25× photoIC$_{50}$ (1.6 µM) of **Pt2** (1 h + 1 h 465 nm), (v) **T13-14** 0.5× photoIC$_{50}$ (3.2 µM) of **Pt2** (1 h + 1 h 465 nm), (vi) **T15-17** 1× photoIC$_{50}$ (6.5 µM) of **Pt2** (1 h + 1 h 465 nm), (vii) **T18-19** 1× photoIC$_{50}$ (6.5 µM) of **Pt2** (1 h + 1 h 465 nm) with 2 h recovery in complex-free media. A minimum of 10 mitochondria were segmented per tomogram.

| **Tomogram number volume (µm$^3$)** | | | | | |
|---|---|---|---|---|---|
| **T1** | **T2** | **T3** | **T4** | **T5** | **T6** |
| 0.20 | 0.18 | 0.53 | 0.24 | 0.17 | 0.11 |
| 0.49 | 0.21 | 0.42 | 0.14 | 0.12 | 0.05 |
| 0.28 | 0.19 | 0.22 | 0.10 | 0.08 | 0.17 |
| 0.09 | 0.26 | 0.25 | 0.17 | 0.13 | 0.21 |
| 0.14 | 0.38 | 0.17 | 0.17 | 0.08 | 0.14 |
| 0.17 | 0.20 | 0.58 | 0.09 | 0.16 | 0.10 |
| 0.27 | 0.11 | 0.45 | 0.15 | 0.04 | 0.11 |
| 0.34 | 0.12 | 0.31 | 0.11 | 0.10 | 0.30 |
| 0.33 | 0.12 | 0.32 | 0.04 | 0.08 | 0.09 |
| 0.17 | 0.13 | 0.29 | 0.10 | 0.10 | 0.23 |
| 0.25±0.12 | 0.19±0.08 | 0.35±0.14 | 0.13±0.06 | 0.11±0.04 | 0.15±0.08 |
| **T7** | **T8** | **T9** | **T10** | **T11** | **T12** |
| 0.06 | 0.05 | 0.05 | 0.03 | 0.35 | 0.09 |
| 0.08 | 0.34 | 0.19 | 0.04 | 0.24 | 0.14 |
| 0.26 | 0.08 | 0.30 | 0.06 | 0.28 | 0.13 |
| 0.09 | 0.07 | 0.15 | 0.07 | 0.20 | 0.15 |
| 0.09 | 0.21 | 0.21 | 0.11 | 0.21 | 0.07 |
| 0.12 | 0.08 | 0.07 | 0.15 | 0.15 | 0.09 |
| 0.06 | 0.08 | 0.15 | 0.19 | 0.17 | 0.19 |
| 0.09 | 0.19 | 0.04 | 0.23 | 0.21 | 0.28 |
| 0.05 | 0.07 | 0.07 | 0.12 | 0.29 | 0.05 |
| 0.05 | 0.13 | 0.03 | 0.16 | 0.27 | 0.13 |
| 0.09±0.06 | 0.13±0.09 | 0.13±0.09 | 0.12±0.07 | 0.24±0.06 | 0.13±0.07 |
| **T13** | **T14** | **T15** | **T18** | **T19** | |
| 0.05 | 0.11 | 0.12 | 0.12 | 0.35 | |
| 0.11 | 0.14 | 0.13 | 0.03 | 0.15 | |
| 0.14 | 0.20 | 0.14 | 0.08 | 0.07 | |
| 0.07 | 0.13 | 0.08 | 0.09 | 0.30 | |
| 0.06 | 0.30 | 0.07 | 0.14 | 0.12 | |
| 0.11 | 0.17 | 0.09 | 0.08 | 0.12 | |
| 0.02 | 0.21 | 0.06 | 0.10 | 0.04 | |
| 0.06 | 0.23 | 0.05 | 0.11 | 0.07 | |
| 0.04 | 0.13 | 0.04 | 0.05 | 0.06 | |
| 0.04 | 0.11 | 0.07 | 0.05 | 0.11 | |
| 0.07±0.04 | 0.17±0.06 | 0.08±0.03 | 0.09±0.03 | 0.14±0.10 | |

**Table S4.** Individual and mean lipid droplet volumes in cryopreserved PC3 cells treated with 0.25–1× photoIC$_{50}$ (1.6-6.5 µM) of **Pt2** under dark (2 h) or blue light (1 h exposure and 1 h 465 nm irradiation) conditions (**T1-19**) as determined in SuRVoS:[6] (i) **T1-3** Untreated control (dark, 2 h), (ii) **T4-6** Untreated control (465 nm, 1 h), (iii) **T7-9** 1× photoIC$_{50}$ (6.5 µM) of **Pt2** (2 h dark), (iv) **T10-12** 0.25× (1.6 µM) photoIC$_{50}$ of **Pt2** (1 h + 1 h 465 nm), (v) **T13-14** 0.5× (3.2 µM) photoIC$_{50}$ of **Pt2** (1 h + 1 h 465 nm), (vi) **T15-17** 1× photoIC$_{50}$ (6.5 µM) of **Pt2** (1 h + 1 h 465 nm), (vii) **T18-19** 1× photoIC$_{50}$ (6.5 µM) of **Pt2** (1 h + 1 h 465 nm) with 2 h recovery in complex-free media. A minimum of 6 lipid droplets were segmented per tomogram.

| **Tomogram number volume (µm$^3$)** | | | | | | |
|---|---|---|---|---|---|---|
| **T1** | **T2** | **T3** | **T4** | **T5** | **T6** | **T7** |
| 0.11 | 0.12 | 0.18 | 0.14 | 0.12 | 0.23 | 0.04 |
| 0.09 | 0.10 | 0.13 | 0.12 | 0.09 | 0.10 | 0.05 |
| 0.05 | 0.08 | 0.14 | 0.16 | 0.07 | 0.17 | 0.04 |
| 0.05 | 0.09 | 0.26 | 0.24 | 0.09 | 0.09 | 0.04 |
| 0.07 | 0.08 | 0.38 | 0.15 | 0.07 | 0.12 | 0.07 |
| 0.06 | 0.09 | 0.31 | 0.10 | 0.12 | 0.12 | 0.04 |
| 0.07±0.02 | 0.09±0.02 | 0.23±0.10 | 0.15±0.05 | 0.09±0.02 | 0.14±0.05 | 0.05±0.01 |
| **T8** | **T9** | **T10** | **T11** | **T12** | **T13** | **T14** |
| 0.32 | 0.02 | 0.04 | 0.11 | 0.12 | 0.17 | 0.14 |
| 0.26 | 0.03 | 0.05 | 0.29 | 0.15 | 0.17 | 0.11 |
| 0.24 | 0.02 | 0.05 | 0.20 | 0.23 | 0.13 | 0.15 |
| 0.18 | 0.03 | 0.04 | 0.20 | 0.12 | 0.16 | 0.09 |
| 0.20 | 0.03 | 0.06 | 0.20 | 0.12 | 0.11 | 0.16 |
| 0.33 | 0.04 | 0.04 | 0.21 | 0.18 | 0.13 | 0.11 |
| 0.25±0.06 | 0.03±0.01 | 0.05±0.01 | 0.15± 0.04 | 0.20±0.06 | 0.15±0.06 | 0.13±0.03 |
| **T15** | **T16** | **T17** | **T18** | **T19** | | |
| 0.04 | 0.05 | 0.05 | 0.11 | 0.09 | | |
| 0.04 | 0.03 | 0.01 | 0.03 | 0.09 | | |
| 0.02 | 0.03 | 0.02 | 0.09 | 0.11 | | |
| 0.02 | 0.01 | 0.04 | 0.15 | 0.05 | | |
| 0.05 | 0.02 | 0.03 | 0.14 | 0.10 | | |
| 0.02 | 0.02 | 0.01 | 0.13 | 0.01 | | |
| 0.03±0.02 | 0.03±0.01 | 0.03±0.01 | 0.11±0.04 | 0.07±0.04 | | |

**Table S5.** The mean mitochondrial and lipid droplet volumes (μm$^3$) in cryopreserved PC3 cells treated with 0.25–1×$IC_{50}$ (1.6-6.5 μM) of **Pt2** under dark (2 h) or blue light (1 h + 1 h 465 nm, 17 J/cm$^2$) conditions (**T1-19**) as determined in SuRVoS:[6] (i) **T1-3** Untreated control (dark, 2 h), (ii) **T4-6** Untreated control (465 nm, 1 h), (iii) **T7-9** 1× photo$IC_{50}$ (6.5 μM) of **Pt2** (2 h dark), (iv) **T10-12** 0.25× photo$IC_{50}$ (1.6 μM) of **Pt2** (1 h + 1 h 465 nm), (v) **T13-14** 0.5× photo$IC_{50}$ (3.2 μM) of **Pt2** (1 h + 1 h 465 nm), (vi) **T15-17** 1× photo$IC_{50}$ (6.5 μM) of **Pt2** (1 h + 1 h 465 nm), (vii) **T18-19** 1× photo$IC_{50}$ (6.5 μM) of **Pt2** (1 h + 1 h 465 nm) with 2 h recovery in complex-free media. A minimum of 10 mitochondria and 6 lipid droplets per tomogram were segmented.

| Conditions | Tomogram number | Mean mitochondrial volume (μm$^3$) | Total lipids | Mean lipid droplet volume (μm$^3$) |
|---|---|---|---|---|
| **Controls (Dark)** | **T1** | 0.25 ± 0.12 | 68 | 0.07 ± 0.02 |
| | **T2** | 0.19 ± 0.08 | 44 | 0.09 ± 0.02 |
| | **T3** | 0.35 ± 0.14 | 53 | 0.23 ± 0.01 |
| **Controls (465 nm)** | **T4** | 0.13 ± 0.06 | 51 | 0.15 ± 0.05 |
| | **T5** | 0.11 ± 0.04 | 45 | 0.09 ± 0.02 |
| | **T6** | 0.15 ± 0.08 | 35 | 0.14 ± 0.05 |
| **1 × $IC_{50}$ (Dark)** | **T7** | 0.09 ± 0.06 | 24 | 0.05 ± 0.01 |
| | **T8** | 0.13 ± 0.09 | 26 | 0.25 ± 0.06 |
| | **T9** | 0.13 ± 0.09 | 28 | 0.03 ± 0.01 |
| **0.25 × $IC_{50}$ (465 nm)** | **T10** | 0.12 ± 0.07 | 17 | 0.05 ± 0.01 |
| | **T11** | 0.24 ± 0.06 | 23 | 0.15 ± 0.04 |
| | **T12** | 0.13 ± 0.07 | 46 | 0.20 ± 0.06 |
| **0.5 × $IC_{50}$ (465 nm)** | **T13** | 0.07 ± 0.04 | 42 | 0.15 ± 0.06 |
| | **T14** | 0.17 ± 0.06 | 36 | 0.13 ± 0.03 |
| **1 × $IC_{50}$ (465 nm)** | **T15** | n.d | 33 | 0.03 ± 0.02 |
| | **T16** | n.d | 26 | 0.03 ± 0.01 |
| | **T17** | 0.08 ± 0.03 | 28 | 0.03 ± 0.01 |
| **1 × $IC_{50}$ (465 nm)[a]** | **T18** | 0.09 ± 0.03 | 6 | 0.11 ± 0.04 |
| | **T19** | 0.14 ± 0.10 | 88 | 0.07 ± 0.04 |

[a] Cells treated with 1× photo$IC_{50}$ (6.5 μM) for 1 h, followed by 1 h irradiation with blue light (465 nm, 17 J/cm$^2$) and 2 h recovery in complex-free media.

**Table S6.** Volumes (μm$^3$) of endosome-like structures observed in two independent PC3 cells treated with 1× photoIC$_{50}$ (IC$_{50}$=6.5 μM) of **Pt2** for 2 h under dark conditions (tomograms **T7** and **T9**, respectively, in Figs S13 and S14). Data were analyzed in SuRVoS.[6]

| | **Tomogram number/Endosomal volume (μm$^3$)** | |
|---|---|---|
| **Endosome number** | **T7** | **T9** |
| **1** | 0.58 | 0.99 |
| **2** | 0.53 | 0.42 |
| **3** | 0.19 | 0.20 |
| **4** | 0.17 | 0.37 |
| **5** | 0.39 | 0.21 |
| **6** | 0.33 | 0.19 |
| **7** | 0.21 | 0.33 |
| **8** | 0.24 | 0.38 |
| **9** | 0.16 | 0.27 |
| **10** | 0.23 | 0.19 |
| **11** | 0.14 | 0.26 |
| **12** | 0.09 | 0.13 |
| **13** | 0.31 | |
| **14** | 0.23 | |
| **15** | 0.30 | |
| **16** | 0.21 | |
| **17** | 0.16 | |
| **Mean** | 0.26 ± 0.13 | 0.33 ± 0.23 |

# ES5 Synchrotron-XRF

**Preparation of silicon nitride membranes.** For nanofocussed XRF experiments at the I14 beamline (Diamond Light Source), silicon nitride ($Si_3N_4$) membranes were used as growth substrates for cells as they are completely transparent towards X-rays in the energy range of the beamline (5-23 keV) and are well-established for use in synchrotron-XRF.[10] $Si_3N_4$ membranes were washed with 70% ethanol (5 min) and 100% ethanol (5 min) and air-dried. 0.01% poly-L-lysine (1-2 drops) were added directly to the grid membrane and left at room temperature for 20 min. The poly-L-lysine was washed off using PBS (2×3 mL). Cell suspensions of *ca.* 7×10$^5$ cells/ mL were prepared in DMEM culture media and 50 µL of this was added directly to each membrane. The membranes were left to incubate for 2 h (37 °C, 5% $CO_2$). After 2 h, 3 mL the *same* cell suspension was added to each membrane and left to incubate for 24 h (37 °C, 5% $CO_2$). After 24 h, the supernatant was removed using a suction pump, and cells were treated with 5× photoIC$_{50}$ of **Pt1** (photoIC$_{50}$=55 µM), **Pt2** (photoIC$_{50}$=6.5 µM) or **cisplatin** (photoIC$_{50}$>100 µM) for (i) 2 h in the dark or (ii) 1 h incubation followed by 1 h irradiation (in drug) with blue light (λ = 465 nm). The supernatant drug was removed and cells washed with HBSS buffer (2 mL/ well). The membranes were dipped in sterile water (3 s), blotted (3 s) and plunge-frozen in 30% liquid ethane:propane mixture. The $Si_3N_4$ membranes were transferred to cryo-vials, covered in pierced parafilm, and freeze-dried for 48 h.

**I14 sample Loading.** $Si_3N_4$ membranes were loaded into the sample holder available at I14 using fine tweezers, with the flat side on the surface (cells facing upwards). The grids were individually screwed into the sample holders, and, loaded vertically onto the beamline (with the screw-side closest to the incoming X-ray beam).

**I14 beamline settings.** All synchrotron-XRF maps were obtained by raster scanning using an incident energy of 14 keV, 100×100 $nm^2$ step size, 0.1 s exposure and using an Si drift four-element detector. The photon flux was estimated by running an AXO (DRESDEN, GmbH) standard $Si_3N_4$ membrane containing known elemental quantifications to further enable quantification of platinum.

**Data analysis.** All elemental maps were fitted in PyMCa toolkit software (ESRF)[11] by aligning known elemental fluorescence emissions up to the incident energy (in this case 14 keV). Cell areas ($\mu m^2$), roundness factors, co-localization statistics and elemental mass fraction quantities were determined using ImageJ software.[12] All displayed elemental maps were generated in ImageJ using 16-colour filters.[12] Intracellular Pt was quantified by calibrating the flux to an AXO standard (containing known concentrations of elements), assuming a maximal cell thickness of 6 µm.[13]

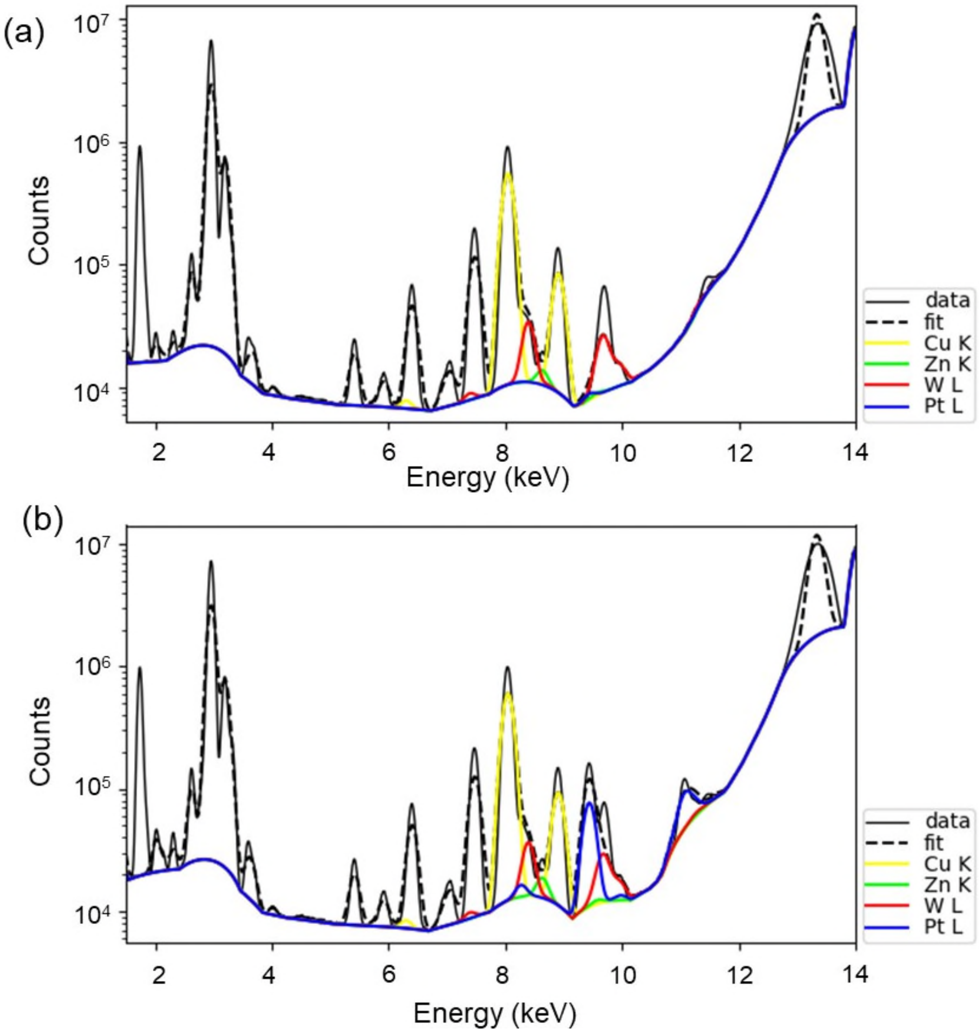


**Figure S24.** Representative XRF spectra for a cryo-fixed and freeze-dried PC3 (human prostate) cancer cell; raster scan: 100×100 $nm^2$ step size, 0.1 s dwell time as obtained by synchrotron-XRF. Data were fitted in PyMCa toolkit (ESRF).[11] The contribution of Cu K (**yellow**), Zn K (**green**), W L (**red**) and Pt L (**blue**) emission lines are shown. Note that Cu and W are scattered from the sample holder. (a) Untreated control. (b) Cell treated with 5× photo$IC_{50}$ (32.5 µM) of **Pt2** for 1 h (protected from light) followed by 1 h irradiation (465 nm, 17 $J/cm^2$).

**Elemental mapping summary.** A total of 18 single PC3 cells were mapped (cells **C1-18**): (i) untreated controls (exposed to 1 h irradiation at 465 nm; Figs S25-27, **C1-3**), (ii) 5× photoIC$_{50}$ (500 µM) cisplatin (1 h followed by 1 h irradiation at 465 nm, Figs S28-30, **C4-6**), (iii) 5× photoIC$_{50}$ (275 µM) of **Pt1** (2 h dark conditions, Figs S31-33, **C7-9**), (iv) 5× photoIC$_{50}$ (275 µM) of **Pt1** (1 h followed by 1 h irradiation at 465 nm, Figs S34-36, **C10-12**), (v) 5× photoIC$_{50}$ (32.5 µM) of **Pt2** (2 h dark conditions, Figs S37-39, **C13-15**), (vi) 5× photoIC$_{50}$ (32.5 µM) of **Pt2** (1 h followed by 1 h irradiation at 465 nm, Figs S40-42, **C16-18**). Representative elemental maps for Pt as well as endogenous K, P and Zn are shown in Figure 5 and show the different cellular distributions for these elements.

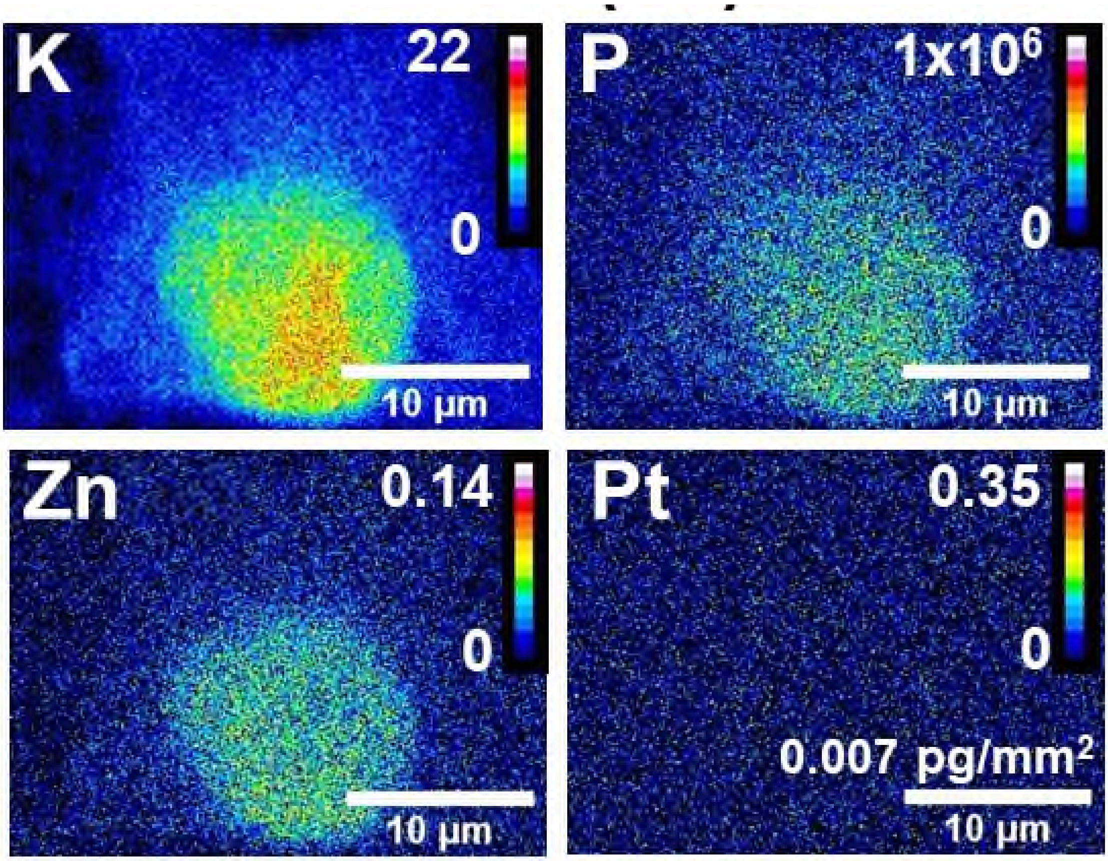


**Figure S25.** Synchrotron-XRF K, P, Zn and Pt elemental maps of a single cryo-fixed and freeze-dried PC3 prostate cancer cell (**C1**) grown on a $Si_3N_4$ membrane and exposed to 1 h irradiation at 465 nm, obtained using incident energy 14 keV at 100×100 nm$^2$ step size. Images were generated in ImageJ software.[12]

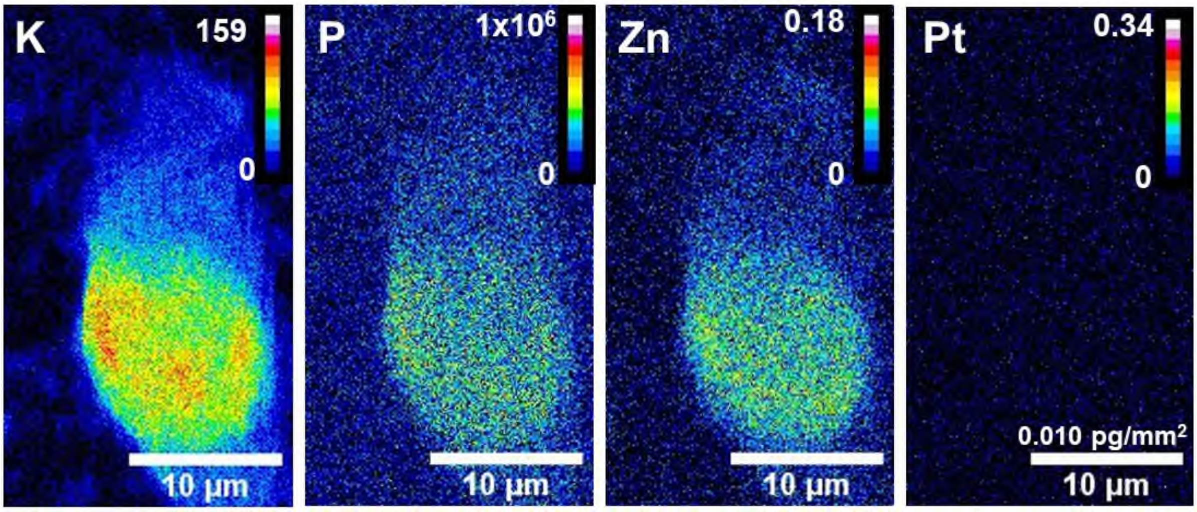


**Figure S26.** Synchrotron-XRF K, P, Zn and Pt elemental maps of a single cryo-fixed and freeze-dried PC3 prostate cancer cell (**C2**) grown on a $Si_3N_4$ membrane and exposed to 1 h irradiation with blue light (465 nm, 17 J/cm$^2$) obtained using incident energy 14 keV at 100×100 nm$^2$ step size. Images were generated in ImageJ software.[12]

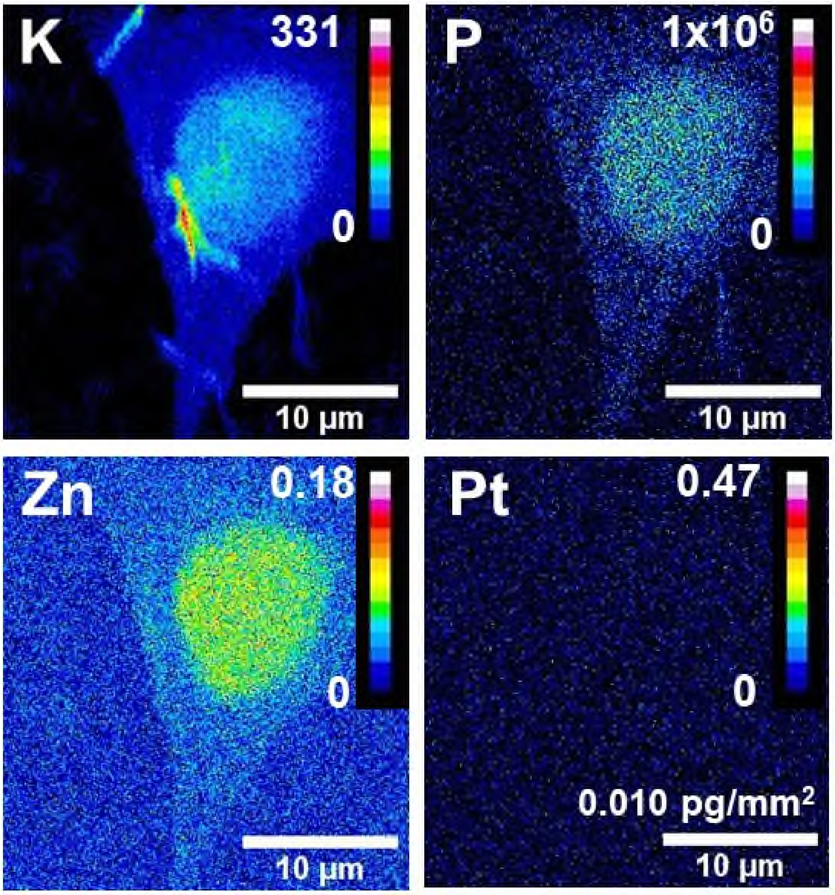


**Figure S27.** Synchrotron-XRF elemental maps of a single cryo-fixed and freeze-dried PC3 prostate cancer cell (**C3**) grown on a $Si_3N_4$ membrane and exposed to 1 h irradiation with blue light (465 nm, 17 J/cm$^2$), obtained using incident energy 14 keV at 100×100 nm$^2$ step size. Images were generated in ImageJ software.[12]

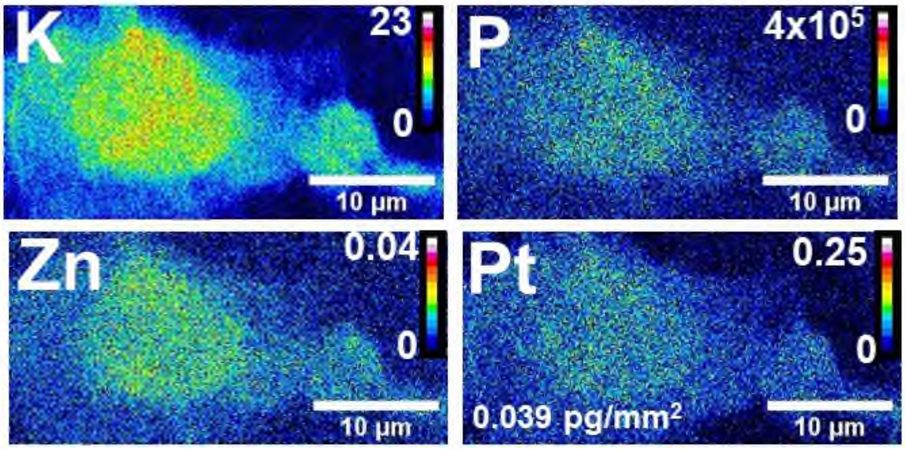


**Figure S28.** Synchrotron-XRF K, P, Zn and Pt elemental maps of a single cryo-fixed and freeze-dried PC3 prostate cancer cell (**C4**) grown on a $Si_3N_4$ membrane and treated with 5× photo$IC_{50}$ (500 µM) **cisplatin** for 1 h followed by 1 h irradiation with blue light (465 nm), obtained using incident energy 14 keV at 100×100 $nm^2$ step size. Images were generated in ImageJ software.[12]

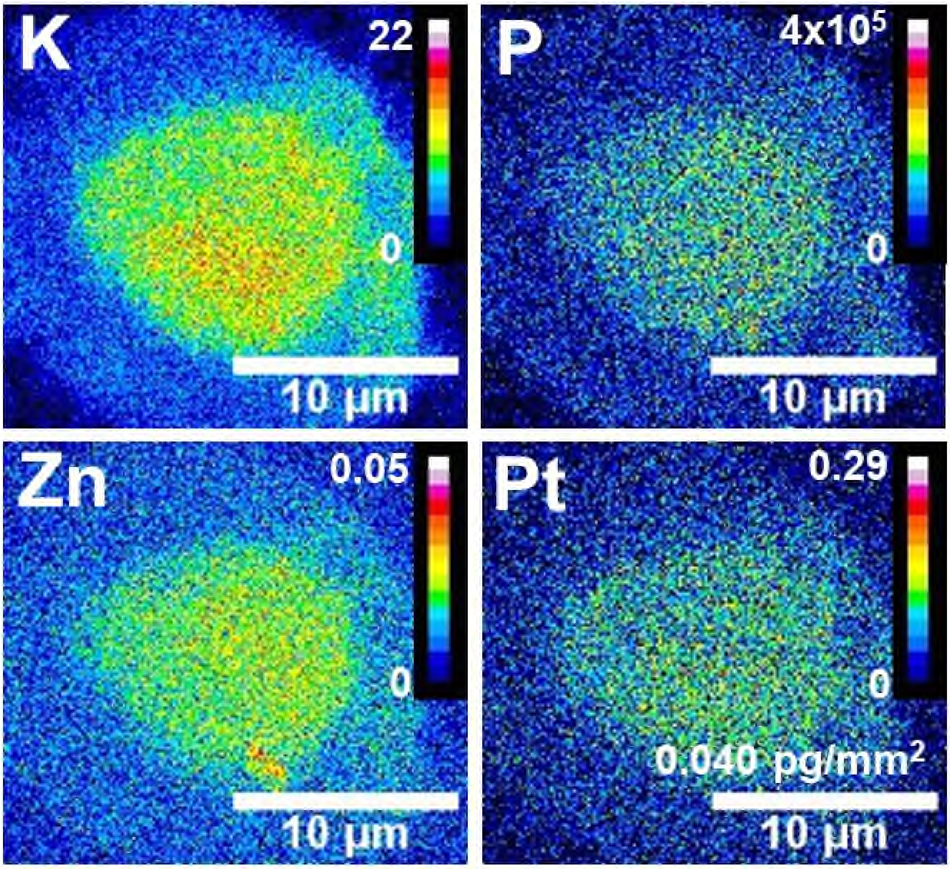


**Figure S29.** Synchrotron-XRF K, P, Zn and Pt elemental maps of a single cryo-fixed and freeze-dried PC3 prostate cancer cell (**C5**) grown on a $Si_3N_4$ membrane and treated with 5× photo$IC_{50}$ (500 µM) **cisplatin** for 1 h followed by 1 h irradiation with blue light (465 nm), obtained using incident energy 14 keV at 100×100 $nm^2$ step size. Images were generated in ImageJ software.[12]

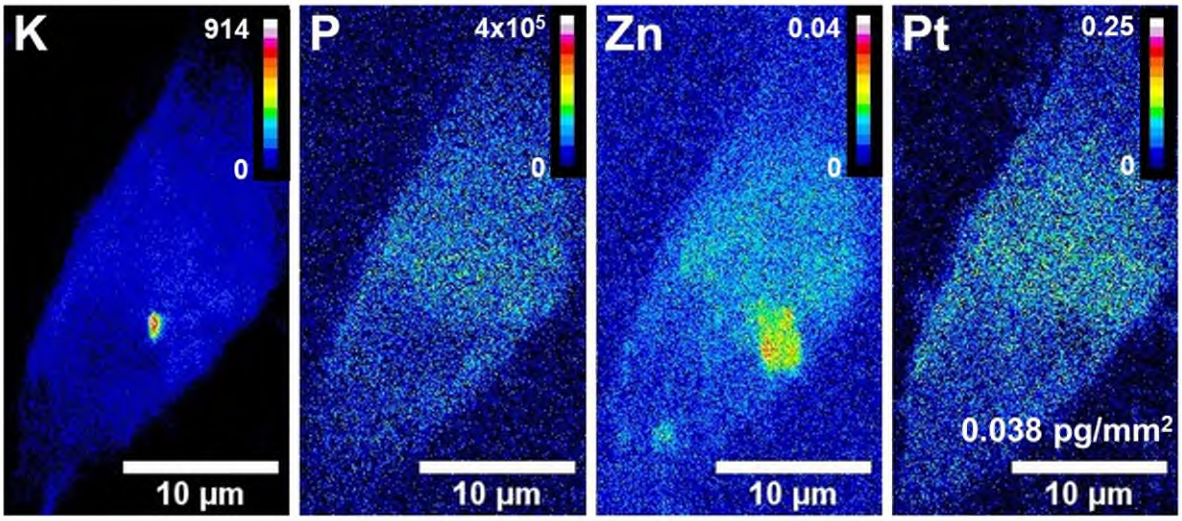


**Figure S30.** Synchrotron-XRF K, P, Zn and Pt elemental maps of a single cryo-fixed and freeze-dried PC3 prostate cancer cell (**C6**) grown on a $Si_3N_4$ membrane and treated with 5× photo$IC_{50}$ (500 µM) **cisplatin** for 1 h followed by 1 h irradiation with blue light (465 nm), obtained using incident energy 14 keV at 100×100 $nm^2$ step size. Images were generated in ImageJ software.[12]

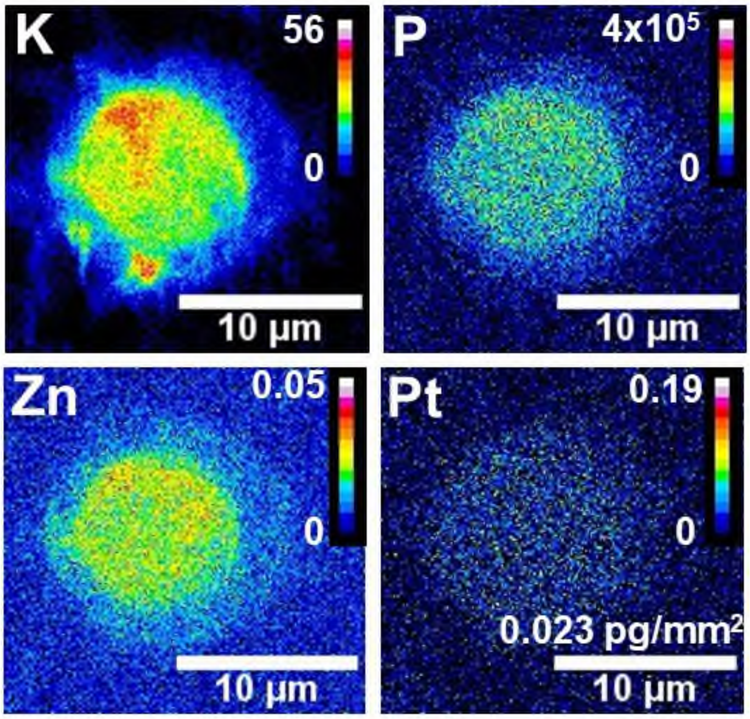


**Figure S31.** Synchrotron-XRF K, P, Zn and Pt elemental maps of a single cryo-fixed and freeze-dried PC3 prostate cancer cell (**C7**) grown on a $Si_3N_4$ membrane and treated with 5× photo$IC_{50}$ (275 µM) **Pt1** for 2 h under dark conditions (protected from the light), obtained using incident energy 14 keV at 100×100 $nm^2$ step size. Images were generated in ImageJ software.[12] The units of the calibration bar are pg/$mm^2$. The mean intracellular quantity of Pt was determined to be 0.023 pg/$mm^2$.

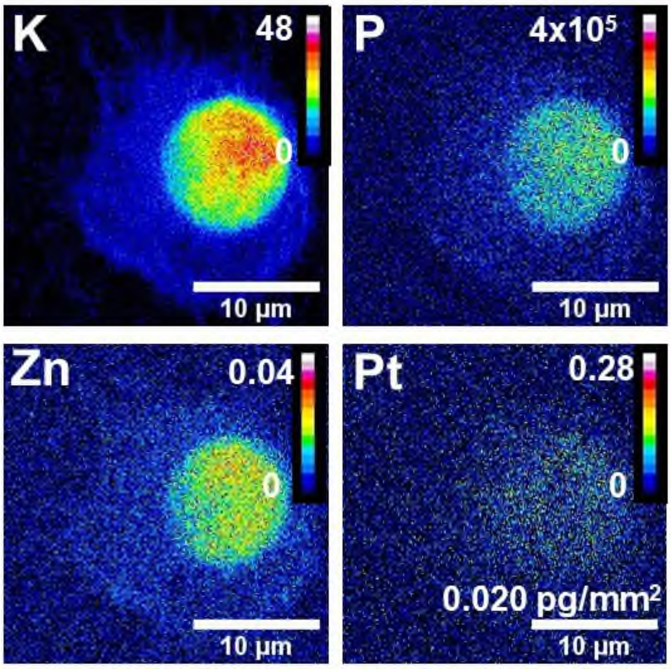


**Figure S32.** Synchrotron-XRF K, P, Zn and Pt elemental maps of a single cryo-fixed and freeze-dried PC3 prostate cancer cell (**C8**) grown on a $Si_3N_4$ membrane and treated with 5× photoIC$_{50}$ (275 µM) **Pt1** for 2 h under dark conditions (protected from the light), obtained using incident energy 14 keV at 100×100 nm$^2$ step size. Images were generated in ImageJ software.[12] The units of the calibration bar are pg/mm$^2$. The mean intracellular quantity of Pt was determined to be 0.020 pg/mm$^2$.

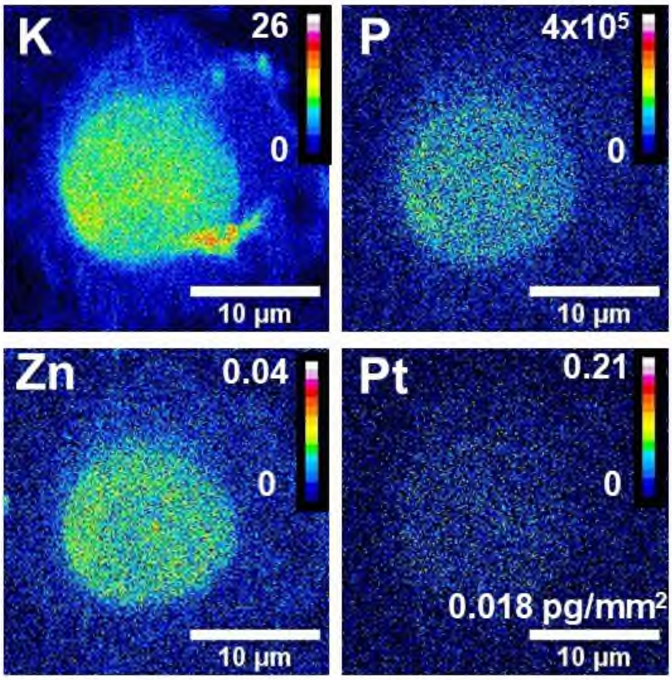


**Figure S33.** Synchrotron-XRF K, P, Zn and Pt elemental maps of a single cryo-fixed and freeze-dried PC3 prostate cancer cell (**C9**) grown on a $Si_3N_4$ membrane and treated with 5× photoIC$_{50}$ (275 µM) **Pt1** for 2 h under dark conditions (protected from the light), obtained using incident energy 14 keV at 100×100 nm$^2$ step size. Images were generated in ImageJ software.[12] The units of the calibration bar are pg/mm$^2$. The mean intracellular quantity of Pt was determined to be 0.018 pg/mm$^2$.

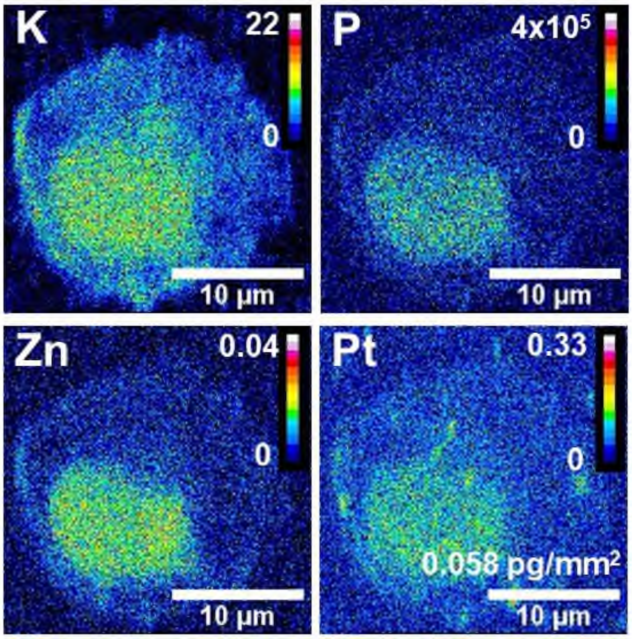


**Figure S34.** Synchrotron-XRF K, P, Zn and Pt elemental maps of a single cryo-fixed and freeze-dried PC3 prostate cancer cell (**C10**) grown on a $Si_3N_4$ membrane and treated with 5× photo$IC_{50}$ (275 µM) **Pt1** for 1 h followed by 1 h irradiation with blue light (465 nm), obtained using incident energy 14 keV 100×100 nm$^2$ step size. Images were generated in ImageJ software.[12] The units of the calibration bar are pg/mm$^2$. The mean intracellular quantity of Pt was determined to be 0.058 pg/mm$^2$.

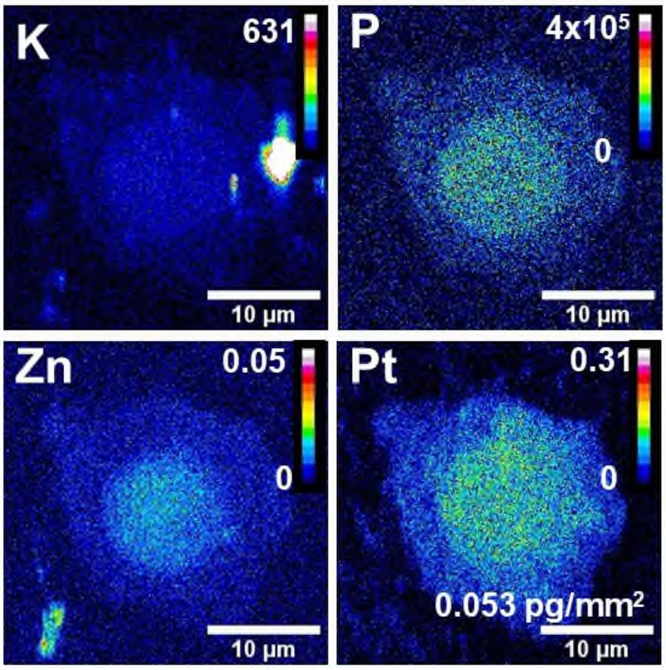


**Figure S35.** Synchrotron-XRF K, P, Zn and Pt elemental maps of a single cryo-fixed and freeze-dried PC3 prostate cancer cell (**C11**) grown on a $Si_3N_4$ membrane and treated with 5× photo$IC_{50}$ (275 µM) **Pt1** for 1 h followed by 1 h irradiation with blue light (465 nm), obtained using incident energy 14 keV 100×100 nm$^2$ step size. Images were generated in ImageJ software.[12] The units of the calibration bar are pg/mm$^2$. The mean intracellular quantity of Pt was determined to be 0.053 pg/mm$^2$.

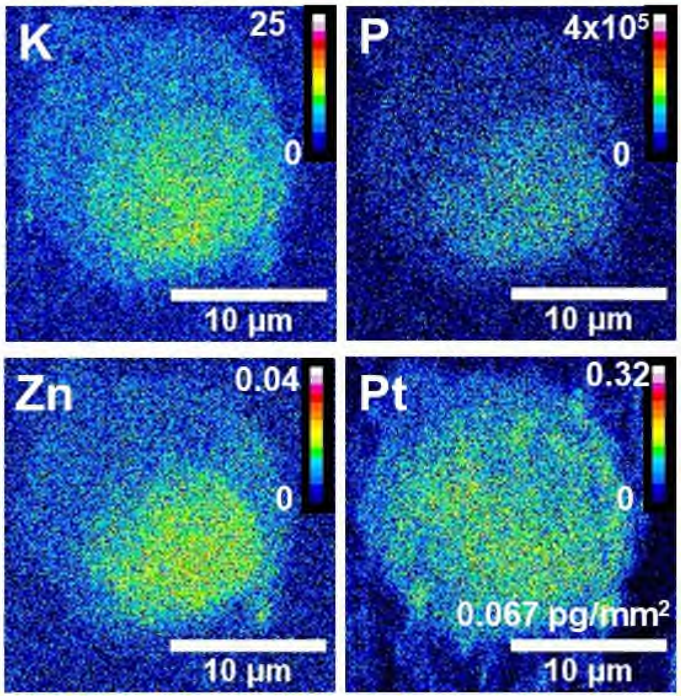


**Figure S36.** Synchrotron-XRF K, P, Zn and Pt elemental maps of a single cryo-fixed and freeze-dried PC3 prostate cancer cell (**C12**) grown on a $Si_3N_4$ membrane and treated with 5× photo$IC_{50}$ (275 µM) **Pt1** for 1 h followed by 1 h irradiation with blue light (465 nm), obtained using incident energy 14 keV at 100×100 $nm^2$ step size. Images were generated in ImageJ software.[12] The units of the calibration bar are pg/$mm^2$. The mean intracellular quantity of Pt was determined to be 0.067 pg/$mm^2$.

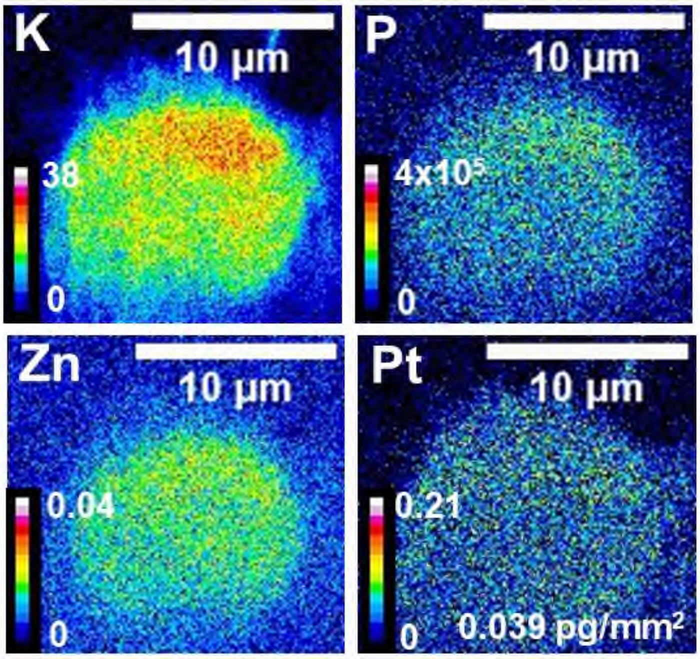


**Figure S37.** Synchrotron-XRF K, P, Zn and Pt elemental maps of a single cryo-fixed and freeze-dried PC3 prostate cancer cell (**C13**) grown on a $Si_3N_4$ membrane and treated with 5× photo$IC_{50}$ (32.5 µM) **Pt2** for 2 h under dark conditions (protected from the light), obtained using incident energy 14 keV at 100×100 $nm^2$ step size. Images were generated in ImageJ software.[12] The units of the calibration bar are pg/$mm^2$. The mean intracellular quantity of Pt was determined to be 0.039 pg/$mm^2$.

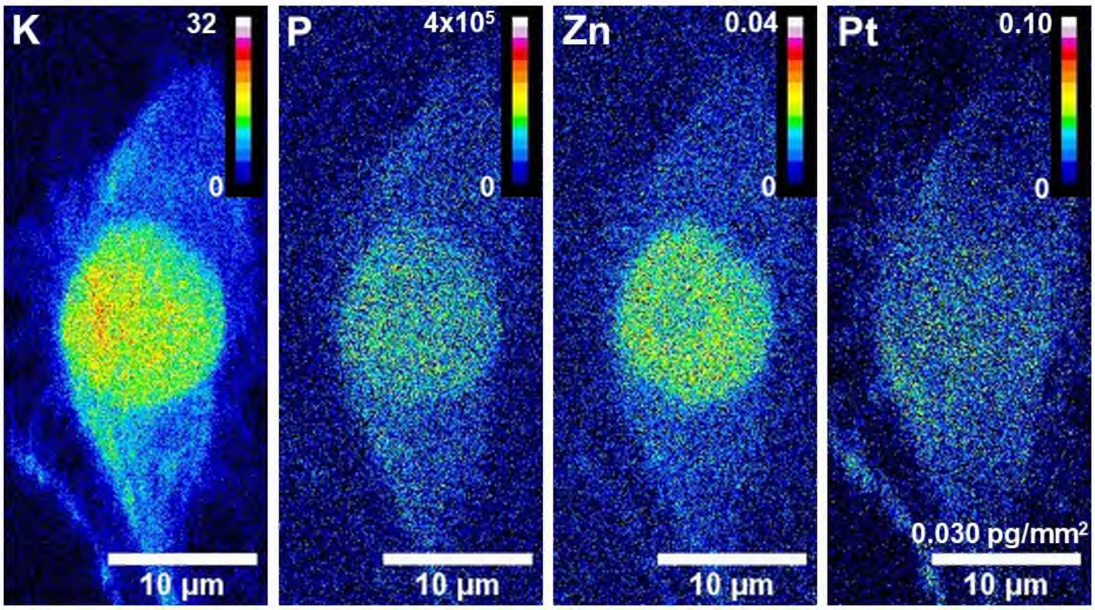


**Figure S38.** Synchrotron-XRF K, P, Zn and Pt elemental maps of a single cryo-fixed and freeze-dried PC3 prostate cancer cell (**C14**) grown on a $Si_3N_4$ membrane and treated with 5× photo$IC_{50}$ (32.5 µM) **Pt2** for 2 h under dark conditions (protected from the light), obtained using incident energy 14 keV at 100×100 $nm^2$ step size. Images were generated in ImageJ software.[12] The units of the calibration bar are pg/$mm^2$. The mean intracellular quantity of Pt was determined to be 0.030 pg/$mm^2$.

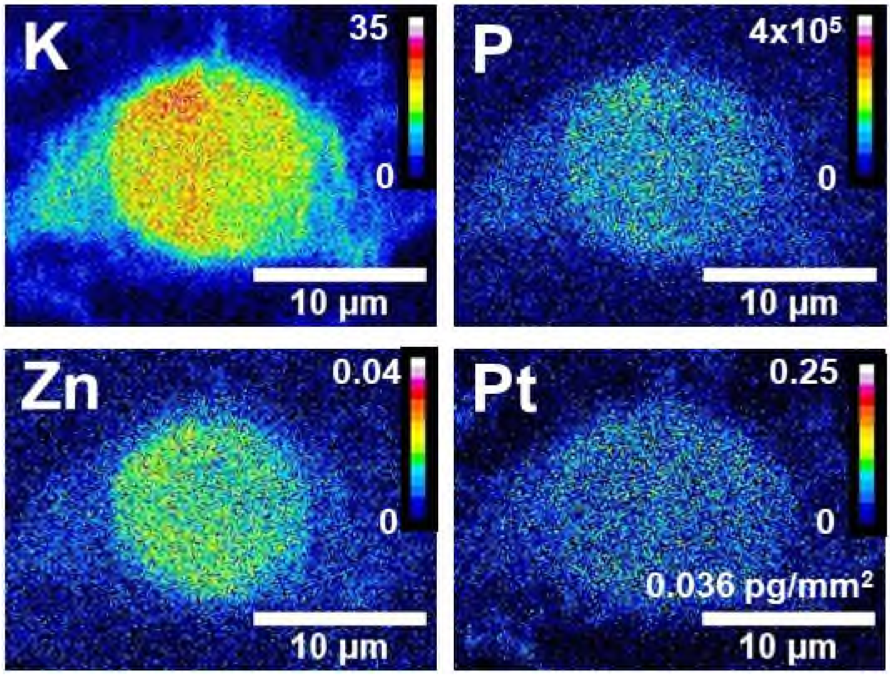


**Figure S39.** Synchrotron-XRF K, P, Zn and Pt elemental maps of a single cryo-fixed and freeze-dried PC3 prostate cancer cell (**C15**) grown on a $Si_3N_4$ membrane and treated with 5× photo$IC_{50}$ (32.5 µM) **Pt2** for 2 h under dark conditions (protected from the light), obtained using incident energy 14 keV at 100×100 $nm^2$ step size. Images were generated in ImageJ software.[12] The units of the calibration bar are pg/$mm^2$. The mean intracellular quantity of Pt was determined to be 0.036 pg/$mm^2$.

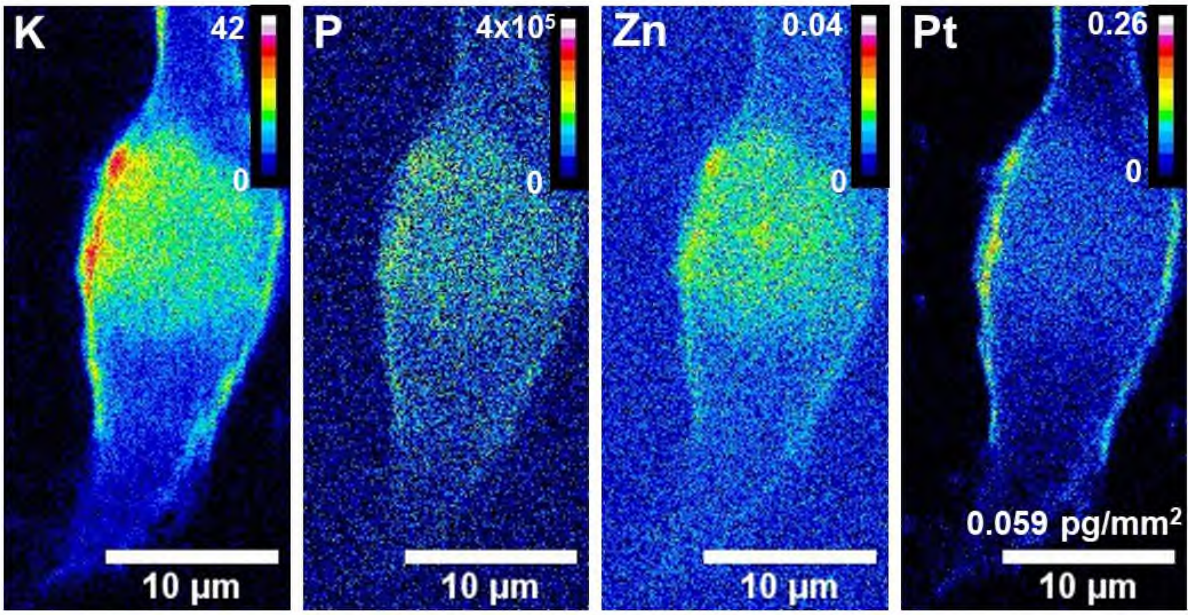


**Figure S40.** Synchrotron-XRF K, P, Zn and Pt elemental maps of a single cryo-fixed and freeze-dried PC3 prostate cancer cell (**C16**) grown on a $Si_3N_4$ membrane and treated with 5× photo$IC_{50}$ (32.5 μM) **Pt2** for 1 h followed by 1 h irradiation with blue light (465 nm), obtained using incident energy 14 keV at 100×100 $nm^2$ step size. Images were generated in ImageJ software.[12] The units of the calibration bar are pg/$mm^2$. The mean intracellular quantity of Pt was determined to be 0.059 pg/$mm^2$.

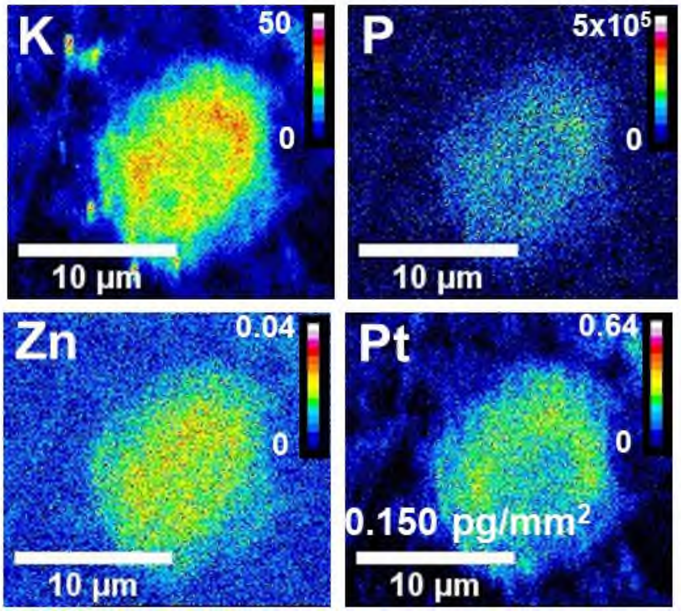


**Figure S41.** Synchrotron-XRF K, P, Zn and Pt elemental maps of a single cryo-fixed and freeze-dried PC3 prostate cancer cell (**C17**) grown on a $Si_3N_4$ membrane and treated with 5× photo$IC_{50}$ (32.5 μM) **Pt2** for 1 h followed by 1 h irradiation with blue light (465 nm), obtained using incident energy 14 keV at 100×100 $nm^2$ step size. Images were generated in ImageJ software.[12] The units of the calibration bar are pg/$mm^2$. The mean intracellular quantity of Pt was determined to be 0.15 pg/$mm^2$.

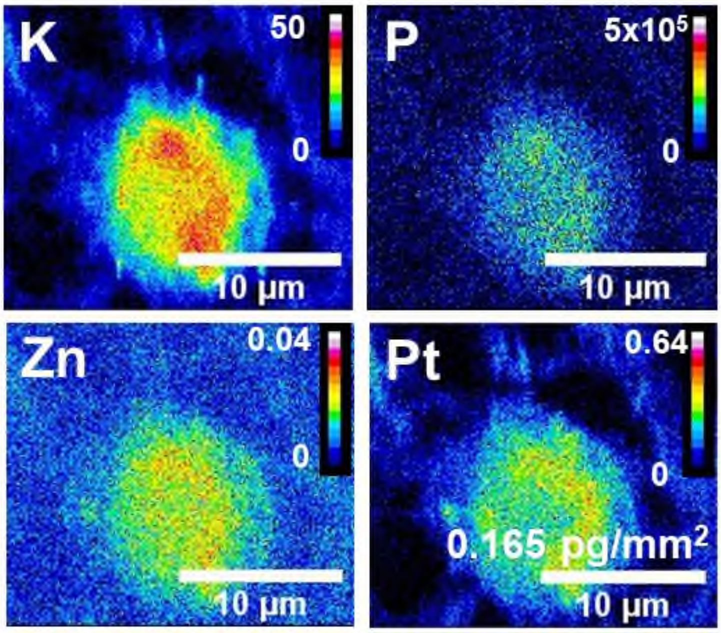


**Figure S42.** Synchrotron-XRF K, P, Zn and Pt elemental maps of a single cryo-fixed and freeze-dried PC3 prostate cancer cell (**C18**) grown on a $Si_3N_4$ membrane and treated with 5× photo$IC_{50}$ (32.5 µM) **Pt2** for 1 h followed by 1 h irradiation with blue light (465 nm), obtained using incident energy 14 keV at 100×100 $nm^2$ step size. Images were generated in ImageJ software.[12] The units of the calibration bar are pg/$mm^2$. The mean intracellular quantity of Pt was determined to be 0.165 pg/$mm^2$.

**Distribution of Pt in cells.** In PC3 cells treated with cisplatin for 1 h and exposed to 1 h irradiation (**C4-6**), Pt was found in both the cell nucleus and the cytoplasm (Fig. 5). The Pt XRF for cells treated with **Pt1** under dark conditions (**C7-10**) was low in intensity, but appears to somewhat co-localize with the spherical area of concentrated Zn in the cell nucleus. Cells treated with the same concentration of **Pt1** but exposed to 1 h blue light (**C10-12**) revealed significantly more intracellular Pt, sparsely distributed in the cell (including the cell nucleus). Cells treated with **Pt2** under dark conditions (**C13-15**) showed significantly less Pt compared to irradiated cells (**C16-18**). Overall, cells treated with **Pt2** showed sparse distributions of Pt (cytoplasm and the nucleus). It is notable that one of the less damaged (elongated) cells treated with **Pt2** exposed to blue light (**C16**) showed highly localized Pt around the perimeter of the cell.

**Cell morphology.** Cryo-fixed and dehydrated PC3 cells exposed to blue light (1 h, 465 nm) revealed clear, stretched-out morphologies (identified by endogenous elements: K, P and Zn) and well-rounded cell nuclei (**C1-3**). Cells treated with cisplatin for 1 h and exposed to 1 h irradiation exhibited a variety of cell morphologies. Cells treated with **Pt1** under dark or irradiated conditions (**C7-12**) appeared more rounded (compared to the untreated controls), with spherical nuclei. Cells treated with **Pt2** under both dark or irradiated conditions (1 h, 465 nm) revealed a variety of morphologies, varying in both size and circularity.

**Area and roundness factors.** The area (μm$^2$) and roundness factors of each individual cell were determined in triplicate using ImageJ software (Table S7). No statistically significant differences in cell areas (μm$^2$) were observed between treated cells (**C4-18**) and the controls (**C1-3**). Similarly, no statistical differences in roundness factors were observed between treated cells (**C4-18**) and untreated cells (**C1-3**). No significant differences in cell area (μm$^2$) or roundness factor were observed between cells treated with **Pt1** under dark and irradiated conditions (**C7-9** with **C10-12**, $p>0.05$) or with cells treated with **Pt2** (**C13-15** with **C16-18**, $p>0.05$). Due to the diverse range of cell morphologies exhibited by cells, the area of cell nuclei and the percentage area of nuclei were determined, revealing no statistically significant differences in the mean area of nuclei with respect to the whole cell area for cells treated with **Pt1**, **Pt2** or cisplatin (Table S9).

**Table S7.** Cell areas (μm$^2$) and roundness factors for cryo-fixed and freeze-dried PC3 cells treated with 5× photo$IC_{50}$ of **Pt1** ($IC_{50}$=55.6±0.9 μM), **Pt2** ($IC_{50}$= 6.48±0.84 μM) or **cisplatin** ($IC_{50}$>100 μM) under both dark (2 h) or light (1 h + 1 h 465 nm, 17 J/cm$^2$) as determined from the S, K, P and Zn XRF elemental maps. Data were analyzed in triplicate in ImageJ.[12]

| Conditions | Cell number | Area (μm$^2$)[a] | Mean | Roundness factor[b] | Mean |
|---|---|---|---|---|---|
| **Controls** | **C1** | 482±5 | 383±75 | 0.87±0.01 | 0.69±0.20 |
| | **C2** | 334±7 | | 0.43±0.01 | |
| | **C3** | 332±5 | | 0.78±0.04 | |
| **Cisplatin (465 nm)** | **C4** | 398±8 | 345±40 | 0.48±0.01 | 0.59±0.24 |
| | **C5** | 313±7 | | 0.88±0.02 | |
| | **C6** | 324±6 | | 0.37±0.01 | |
| **Pt1 (Dark)** | **C7** | 151±3 | 283±118 | 0.92±0.01 | 0.94±0.03 |
| | **C8** | 276±5 | | 0.94±0.02 | |
| | **C9** | 423±6 | | 0.97±0.01 | |
| **Pt1 (465 nm)** | **C10** | 319±2 | 302±55 | 0.91±0.01 | 0.90±0.01 |
| | **C11** | 355±6 | | 0.82±0.01 | |
| | **C12** | 231±5 | | 0.96±0.01 | |
| **Pt2 (Dark)** | **C13** | 133±2 | 211±67 | 0.97±0.02 | 0.64±0.26 |
| | **C14** | 287±2 | | 0.37±0.02 | |
| | **C15** | 212±2 | | 0.59±0.02 | |
| **Pt2 (465 nm)** | **C16** | 265±6 | 175±68 | 0.33±0.01 | 0.69±0.27 |
| | **C17** | 141±2 | | 0.86±0.02 | |
| | **C18** | 119±4 | | 0.89±0.02 | |

[a] Cell areas (μm$^2$) of PC3 cells treated with **Pt1**, **Pt2** or **cisplatin** under dark (2 h drug exposure) or irradiated (1 h drug exposure, 1 h irradiation with 465 nm) conditions as determined in triplicate using ImageJ. [b] Roundness factors of the same individual cells as calculated in ImageJ.

**Table S8.** Individual areas of cell nuclei (μm$^2$) and the percentage of intracellular area which is occupied by nuclei in cryofixed and freeze-dried PC3 cells treated with 5× photo$IC_{50}$ of **Pt1** ($IC_{50}$= 55.6±0.9 μM), **Pt2** ($IC_{50}$=6.48±0.84 μM) or **cisplatin** ($IC_{50}$>100 μM) under dark (2 h) or light (1 h + 1 h 465 nm, 17 J/cm$^2$) conditions as determined from the Zn XRF elemental maps.

| Conditions | Cell number | Area of nucleus (μm$^2$) | Area nucleus (%) | Mean area nucleus (%) |
|---|---|---|---|---|
| **Controls** | **C1** | 139±4 | 30 | 32±7 |
| | **C2** | 136±3 | 41 | |
| | **C3** | 92±3 | 28 | |
| **Cisplatin (465 nm)** | **C4** | 123±1 | 31 | 30±1 |
| | **C5** | 92±3 | 30 | |
| | **C6** | 97±5 | 30 | |
| **Pt1 (Dark)** | **C7** | 66±1 | 43 | 35±7 |
| | **C8** | 82±3 | 30 | |
| | **C9** | 139±1 | 33 | |
| **Pt1 (465 nm)** | **C10** | 81±4 | 25 | 30±5 |
| | **C11** | 105±4 | 30 | |
| | **C12** | 79±2 | 34 | |
| **Pt2 (Dark)** | **C13** | 91±2 | 68 | 52±16 |
| | **C14** | 106±5 | 37 | |
| | **C15** | 107±3 | 50 | |
| **Pt2 (465 nm)** | **C16** | 94±1 | 36 | 46±11 |
| | **C17** | 82±2 | 58 | |
| | **C18** | 53±2 | 45 | |

**Table S9.** Intracellular quantities of Pt (ppm) in cryopreserved and freeze-dried PC3 cells treated with 5× photo$IC_{50}$ of **Pt1** ($IC_{50}$=55.6±0.9 μM), **Pt2** ($IC_{50}$=6.48±0.84 μM) or **cisplatin** ($IC_{50}$> 100 μM) under dark (2 h) or light (1 h + 1 h 465 nm, 17 J/cm$^2$), conditions as determined from the synchrotron-XRF maps.

| Conditions | Cell | [Pt] per cell (ppm) | Mean [Pt] / cell (ppm) | % Pt / nucleus | Mean % Pt in nucleus |
|---|---|---|---|---|---|
| **Cisplatin** | **C4** | 5.40±0.04 | 5.5±0.2 | 40±1 | 42±8 |
| | **C5** | 5.68±0.05 | | 53±1 | |
| | **C6** | 5.34±0.02 | | 34±1 | |
| **Pt1 (Dark)** | **C7** | 3.21±0.01 | 2.8±0.3 | 88±4 | 74±15 |
| | **C8** | 2.75±0.02 | | 55±2 | |
| | **C9** | 2.51±0.01 | | 78±3 | |
| **Pt1 (465 nm)** | **C10** | 8.10±0.05 | 8.2±0.8 | 52±4 | 57±9 |
| | **C11** | 7.4±0.1 | | 50±2 | |
| | **C12** | 9.2±0.1 | | 69±3 | |
| **Pt2 (Dark)** | **C13** | 5.41±0.07 | 4.9±0.6 | 70±7 | 52±17 |
| | **C14** | 4.12±0.02 | | 31±3 | |
| | **C15** | 5.02±0.01 | | 54±2 | |
| **Pt2 (465 nm)** | **C16** | 8.21±0.08 | 17±7 | 36±5 | 55±15 |
| | **C17** | 20.9±0.2 | | 64±8 | |
| | **C18** | 22.88±0.03 | | 64±4 | |

**Elemental co-localization.** Correlations between the distribution of Pt and Zn in PC3 cells were determined for cells treated with cisplatin (**C4-6**), **Pt1** (**C7-12**) and **Pt2** (**C13-18**), and are reported as Pearson R-values (r) and Spearman Rank Correlations (Table S11). Cells treated with cisplatin (**C4-6**) showed moderate correlation between Pt and Zn (mean r=0.34±0.03). Intracellular Pt in cells treated with **Pt1** under dark conditions (**C7-10**) showed a moderate co-localization with Zn (r=0.14–0.23; mean=0.19±0.05). Interestingly, the co-localization between Pt and Zn was significantly enhanced in cells treated with **Pt1** and exposed to blue light (**C10-12**, ranging from r=0.40–0.49; mean=0.44±0.05) when compared to dark conditions (r = 0.19±0.05, $p$=0.0036). The same trend was observed for cells treated with **Pt2** in the dark (**C13-15**, mean r=0.34±0.04) and irradiated (**C16-18**, mean r=0.58±0.08; $p$=0.0423).

**Table S10.** Pearson's R-values and Spearman Rank coefficients between Pt and Zn for PC3 prostate cancer cells treated with 5× photo$IC_{50}$ of **Pt1** ($IC_{50}$=55.6±0.9 μM), **Pt2** ($IC_{50}$=6.48±0.84 μM) or **cisplatin** ($IC_{50}$>100 μM) under dark or light (λ = 465 nm) conditions and cryo-fixed and freeze-dried, as determined from the XRF elemental maps.

| Conditions | Cell number | Pearson's R-value[a] | Spearman rank coefficient[a] |
|---|---|---|---|
| **Cisplatin** | **C4** | 0.30 | 0.31 |
| | **C5** | 0.38 | 0.36 |
| | **C6** | 0.33 | 0.35 |
| **Pt1 (Dark)** | **C7** | 0.23 | 0.17 |
| | **C8** | 0.20 | 0.12 |
| | **C9** | 0.14 | 0.11 |
| **Pt1 (465 nm)** | **C10** | 0.49 | 0.44 |
| | **C11** | 0.42 | 0.38 |
| | **C12** | 0.40 | 0.41 |
| **Pt2 (Dark)** | **C13** | 0.35 | 0.38 |
| | **C14** | 0.29 | 0.24 |
| | **C15** | 0.37 | 0.34 |
| **Pt2 (465 nm)** | **C16** | 0.48 | 0.46 |
| | **C17** | 0.63 | 0.57 |
| | **C18** | 0.62 | 0.59 |

[a] Statistical co-localization (Pearson's R-values and Spearman rank coefficients) of intracellular platinum and zinc as calculated in ImageJ.[12]

## ES6 XANES

**Preparation of $Si_3N_4$ membranes.** $Si_3N_4$ membranes were prepared as previously described (**ES5**, page S26). Cells were treated with 5× photo$IC_{50}$ of **Pt1** (photo$IC_{50}$=55 µM) or **Pt2** (photo$IC_{50}$=6.5 µM) for (i) 2 h in the dark or (ii) 1 h incubation followed by 1 h irradiation (in the presence of the complex) with blue light (λ = 465 nm).

**Preparation of solid pellets**. Reference samples were prepared for XANES analysis. In addition to a metallic Pt foil, solid pellet samples of cisplatin, potassium tetrachloroplatinate ($K_2PtCl_4$), potassium hexachloroplatinate ($K_2PtCl_6$), diamminetetrachloroplatinum ($Pt(NH_3)_2Cl_4$) and **Pt1** were prepared by grinding 3-4 mg with 25-30 mg of dried cellulose using a pestle and mortar and formed in a 10 mm die by applying a 1.5 T force with a pellet press (Specac, UK). The resulting pellets were sealed in Kapton tape to protect them from the environment and to allow labelling. Because of the light sensitivity of $Pt^{IV}$ photoactivatable complex **Pt1**, the pellet was prepared under dark conditions and taped in aluminized mylar to protect it from light.

**Analysis of Pt standards.** In order to improve signal quality and offer a user-friendly experience when dealing with pellets and foils, reference samples are measured upstream from the focusing optics by placing them between two ion chambers which monitor the position of the full beam. XANES spectra were acquired in the energy range 11.46–11.73 keV, across the Pt $L_3$-edge (11.57 keV). The energy stepping was variable, with step sizes ranging from 8 eV away from the edge to 0.5 eV across the edge. Typical analysis of solid pellets and the metal sheet took no longer than 30 min each, as spatial resolution was not required.

**Pt standards summary.** XANES spectra of cisplatin and $K_2PtCl_4$ (white lines at 11571.2 and 11570.7 keV, respectively) were similar, owing to their 2+ oxidation states and square-planar coordination. They were also similar to the XANES spectra of other Pt(II) compounds reported in the literature. The XANES spectra of $K_2[PtCl_6]$, *cis*-$[Pt(NH_3)_2Cl_4]$ and **Pt1** with white lines at 11571.9, 11572.3 and 11573.4 keV, respectively, were shifted to higher energy (by *ca.* 2 eV) and had more intense absorption edges compared to the Pt(II) compounds. The white line of the Pt(0) metal sheet was at 11570.6 keV.

**Validation of compressed-sensing method.** For $Si_3N_4$ cell samples the beam was focussed to a 50 nm spot, and the sample was raster-scanned across region of interests (ROI) previously selected from XRF mapping. At each point of the ROI a full XRF spectrum is acquired, and element-specific images are derived by extracting the corresponding signal from the spectrum. In addition to the traditional data acquisition method of scanning the energy with the monochromator, a compressed sensing approach currently in development at I14 was applied to some samples as a method test. This involved acquiring data at only 8 selected energy points (compressed-sensing) in the energy range (11.4692, 11.5748, 11.5758, 11.5778, 11.5813, 11.5823, 11.6033, 11.7593 keV) rather than the 150 of the default (energy-scanning) method.

***(i) Energy-scanning.*** This is the traditional XANES data acquisition method, which scans the energy with the monochromator (150 energies). The analysis of 5×5 $µm^2$ regions using this method took *ca.* 10-12 h.

***(ii) Compressed-sensing.*** XANES data acquisition using 8 selected energy points in the energy range (11.4692-11.7593 keV) rather than the 150 of the default (energy-scanning) method. The analysis of 5×5 $µm^2$ regions using this method took *ca.* 1-1.5 h, i.e. was up to 10× faster than the conventional energy-scanning method.

Preliminary XANES studies were performed on cryo-fixed and freeze-dried PC3 cells treated with 5× photo$IC_{50}$ of **Pt1** (275 µM) for 1 h, followed by 1 h irradiation with blue light (465 nm, 17 J/cm$^2$) over the Pt $L_3$-edge (11.57 keV) using two methods: (i) energy-scanning, (ii) compressed-sensing. Two independent 5×5 µm$^2$ Pt-containing regions of interest (ROIs) were analyzed by XANES mapping using the nanofocussed beam (50×70 nm) in the range 11.46-11.73 keV (0.2 s dwell time, 200 nm step size) using both methods. The energy stepping was variable, with step sizes ranging from 8 eV away from the edge to 0.5 eV across the edge. Spatially-resolved XANES spectra were constructed from XRF scans at the specified energies, generating a full spatially-resolved XRF map at each energy over the Pt L-edge. In this experiment, the XRF maps at each energy in the range 11.46–11.73 keV were averaged as opposed to obtaining spatially-resolved (pixel-by-pixel).

**Extraction of XANES data.** The XRF peaks of alignment elements (known elements in the sample) were integrated to produce 1 value per pixel using Python software. The images were then stacked and unwanted information removed. From this, the same alignment parameters were applied to the Pt XRF peak ($L_3M_5$=9.44 keV). Principle Component Analysis (mathematical decomposition) was performed on the XRF spectra, followed by cluster analysis (both in Python) to obtain de-noised XANES spectra.[14]

**Normalization of XANES spectra**. XANES spectra were analyzed in Athena XAS Data Analysis Software.[15] Data were normalized using edge-step normalization formulae. Edge-step normalization extrapolates the difference between pre-edge subtraction and post-edge regression to the edge itself. In this case, pre-edge normalization was performed in the energy range 11.47-11.54 keV and the post-edge normalization was performed in the range 11.59-11.75 keV, and a normalization order = 2.

**Linear Combination Fitting (LCF) analysis of Pt1 treated cells.** A linear combination fit (LCF) of the normalized data was performed (11.55- 11.60 keV), revealing ca. 80% intracellular $Pt^{IV}$ by the “compressed-sensing” method and ca. 75% Pt(IV) by the “energy-scanning” XANES method (Table S11).

**Table S11.** Percentages of intracellular $Pt^{IV}$ (%) in cryofixed and dried PC3 cells treated with 5× photo$IC_{50}$ **Pt1** ($IC_{50}$=55.6±0.9 µM) under blue light conditions (1 h + 1 h 465 nm, 17 J/cm$^2$), determined by performing a linear combination fit (LCF) on the normalized data.

| Method | Pt(II) standard | Pt(IV) standard | Pt(IV) (%) |
|---|---|---|---|
| **Energy-scanning** | **Cisplatin** | **Pt1** | 76.5±1.7 |
| | **$K_2PtCl_4$** | **Pt1** | 74.2±2.0 |
| **Compressed-sensing** | **Cisplatin** | **Pt1** | 80.3±2.2 |
| | **$K_2PtCl_4$** | **Pt1** | 81.8±1.9 |

**Peak height normalization.** Due to similarities in peak energy and shape of $Pt^{II}$ and $Pt^{IV}$, conventional curve-fitting techniques cannot be used to reliably determine the relative proportions of $Pt^{II}$:$Pt^{IV}$ species in a mixture. Hence, a method developed by Hambley et. al specifically for $Pt^{IV}/Pt^{II}$ systems was also used to analyze the data. This method normalizes the maximal edge-absorption (height of peak) with the post-edge minima after the white line (Table S12).[16-17] The normalized peak ratios (a/b) of cells treated with **Pt1** were determined to be 1.68±0.02 and 1.74±0.03, for energy-scanning and compressed-sensing, respectively (Table S12). No statistically significant differences in a/b were observed between the two acquisition methods, thus, validating the use of compressed-sensing method.

**XANES of Pt2-treated cells.** Cryo-fixed and freeze-dried PC3 (human prostate) cancer cells were treated with 5× photo$IC_{50}$ of **Pt2** (photo$IC_{50}$=6.5 µM) for (i) 2 h in the dark or (ii) 1 h incubation followed by 1 h irradiation (in drug) with blue light (λ=465 nm, 17 J/$cm^2$) were analyzed by XANES using the compressed-sensing method. A total of 3 independent 15×15 $\mu m^2$ Pt-containing cellular ROIs per condition (dark and light) whole cells were mapped at both a low resolution (0.4 s dwell time, 500 nm step size) and high resolution (0.1 s dwell time, 100 nm step size) using the compressed-sensing method (with energies 11.4692, 11.5748, 11.5758, 11.5778, 11.5813, 11.5823, 11.6033, 11.7593 keV) across the Pt $L_3$-edge (11.57 keV), using the nanofocussed beam (50×70 nm). Two of the three 15×15 $\mu m^2$ regions analyzed per condition were acquired using 0.1 s dwell time and 100 nm step size (Fig. S44-S45). The final 15×15 $\mu m^2$ regions analyzed per condition were acquired using 0.4 s dwell time and 500 nm step size (Fig. 7, Figs. S44, S45). Spatially-resolved XANES spectra were constructed from XRF scans at the specified energies, generating a full spatially-resolved XRF map at each energy over the Pt-edge. Principle Component Analysis (mathematical decomposition) was performed on the XRF spectra, followed by cluster analysis (both in Python) to obtain XANES spectra. XANES spectra were analyzed in Athena XAS Data Analysis Software.[15] In this experiment, the XRF maps at each energy in the range 11.46–11.73 keV were averaged as opposed to spatially-resolved (pixel-by-pixel) mapping.

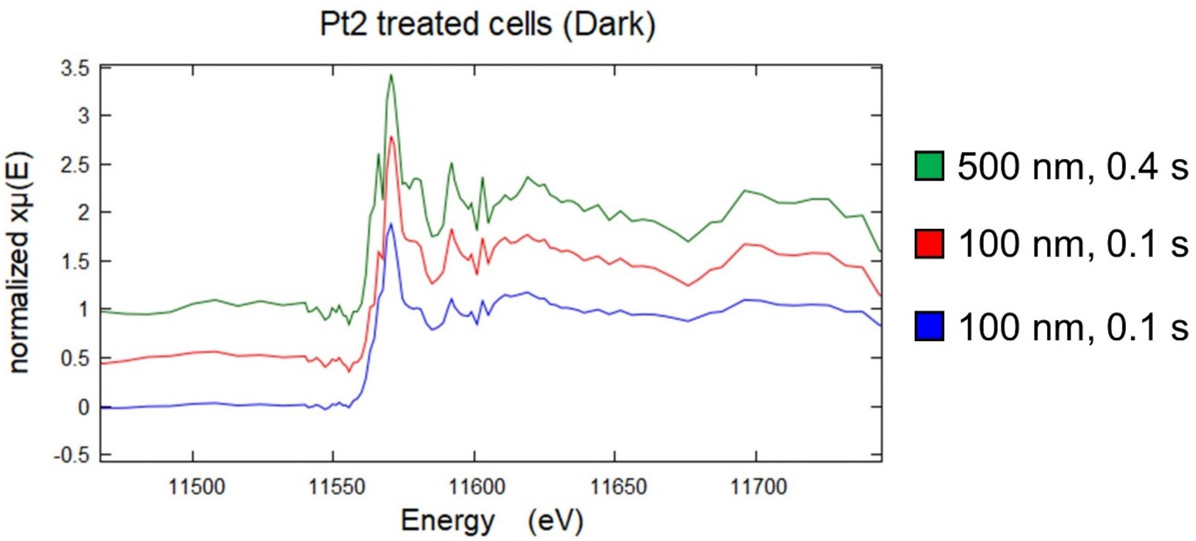


**Figure S43.** Stacked plot of normalized XANES spectra of intracellular Pt in two independent 15×15 $\mu m^2$ regions of cryo-fixed and dried PC3 cells treated with 5× photo$IC_{50}$ (32.5 μM) of **Pt2** under dark conditions (2 h protected from light) using either (i) 100 nm step size and 0.1 s exposure (**red** and **blue**) or (ii) 500 nm step size and 0.4 s exposure (**green**). The spectra were analyzed in Athena XAS Data Analysis Software.[15] Pre-edge normalization was performed in the energy range 11.47-11.54 keV and the post-edge normalization was performed in the range 11.59-11.75 keV, and a normalization order = 2. Only the XANES region is shown.

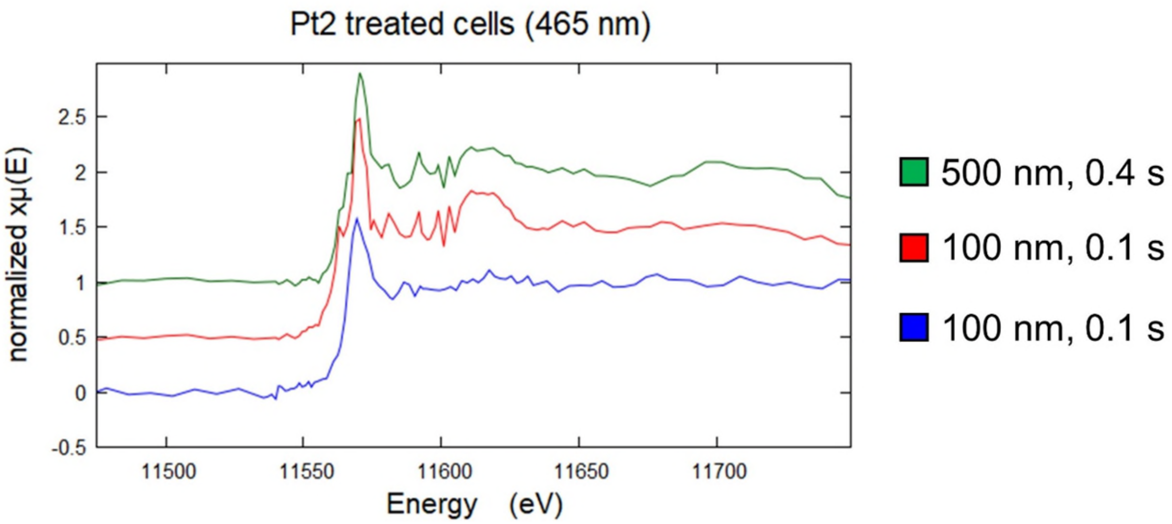


**Figure S44.** Stacked plot of normalized XANES spectra of intracellular Pt in two independent 5×5 $\mu m^2$ regions of cryo-fixed and dried PC3 cells treated with 5× photo$IC_{50}$ (32.5 μM) of **Pt2** under blue light conditions (1 h exposure +1 h 465 nm, 17 $J/cm^2$) using either (i) 100 nm step size and 0.1 s exposure (**red** and **green**) or (ii) 500 nm step size and 0.4 s exposure (**blue**). Spectra were analyzed in Athena XAS Data Analysis Software.[15] Pre-edge normalization was performed in the energy range 11.47-11.54 keV and the post-edge normalization was performed in the range 11.59-11.75 keV, and a normalization order = 2. Only the XANES region is shown.

## Linear Combination Fitting (LCF) analysis of Pt2 treated cells.

Linear combination fit (LCF) analysis was performed on the averaged spectra obtained from 15×15 µm$^2$ regions of cells treated with **Pt2** under dark conditions (Table S12). This revealed 100±11 and 100±24% Pt(IV) for the two regions analyzed at higher resolution (100 nm, 0.1 s), and 100±30 % for the single region analyzed at lower resolution (500 nm, 0.4 s), when using cisplatin and **Pt1** as LCF standards (Table 1). Similarly, LCF using $K_2PtCl_4$ and **Pt1** as standards revealed 94.6±9.3 and 100±20% Pt(IV) (low resolution) and 100±26% Pt(IV) (high resolution). LCF analysis was performed on the averaged spectra obtained from 15×15 µm$^2$ regions of cells treated with **Pt2** and then irradiated (Table 1). This revealed 95.2±16.0 and 100.0±16.5% Pt(IV) for the two regions analyzed at higher resolution (100 nm, 0.1 s), and 68.5±6.7% for the single region analyzed at lower resolution (500 nm, 0.4 s), when using cisplatin and **Pt1** as LCF standards (Table 1). LCF using $K_2PtCl_4$ and **Pt1** as standards revealed 87.1±13.3 and 92.9±13.8% Pt(IV) (low resolution) and 73.5±5.7% Pt(IV) (high resolution).

**Table S12**. Comparison of the amounts of $Pt^{IV}$ and $Pt^{II}$ in PC3 human prostate cancer cells after treatment with coumarin complex **Pt2** in the dark or after irradiation with blue light.

| Condition[a] | Region[b] | White line (eV) | Dwell time (s) | Step size (nm) | Pt(II) standard | Pt(IV) standard | Pt(IV) (%)[c] |
|---|---|---|---|---|---|---|---|
| Dark | 1 | 11570.5 | 0.1 | 100 | Cisplatin | **Pt1** | 100±11 |
| | | | 0.1 | 100 | $K_2PtCl_4$ | **Pt1** | 94.6±9.3 |
| | 2 | 11570.5 | 0.1 | 100 | Cisplatin | **Pt1** | 100±24 |
| | | | 0.1 | 100 | $K_2PtCl_4$ | **Pt1** | 100±20 |
| | 3 | 11570.7 | 0.4 | 500 | Cisplatin | **Pt1** | 100±30 |
| | | | 0.4 | 500 | $K_2PtCl_4$ | **Pt1** | 100±26 |
| Irradiated | 1 | 11570.4 | 0.1 | 100 | Cisplatin | **Pt1** | 95.2±16.0 |
| | | | 0.1 | 100 | $K_2PtCl_4$ | **Pt1** | 87.1±13.3 |
| | 2 | 11570.3 | 0.1 | 100 | Cisplatin | **Pt1** | 100±16.5 |
| | | | 0.1 | 100 | $K_2PtCl_4$ | **Pt1** | 92.9±13.8 |
| | 3 | 11570.2 | 0.4 | 500 | Cisplatin | **Pt1** | 68.5±6.7 |
| | | | 0.4 | 500 | $K_2PtCl_4$ | **Pt1** | 73.5±5.7 |

[a] Whole PC3 cells were treated with 5× photo$IC_{50}$ of **Pt2** under dark (2 h) or irradiated conditions (1 h exposure + 1 h 465 nm, 17 J/cm$^2$) followed by cryofixation and dehydration. [c] Intracellular percentages of $Pt^{IV}$ and $Pt^{II}$ were determined by performing a linear combination fit (LCF) on the normalized data for X-ray absorption edges.

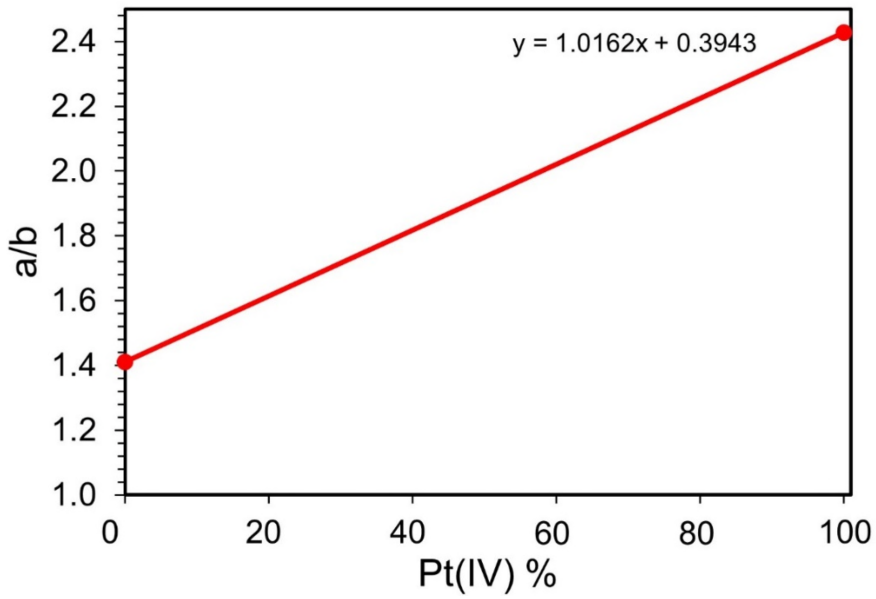


**Figure S45.** Percentage Pt(IV) vs. normalized peak height ratio (a/b) using an average of the Pt(II) standards (a/b = 1.41 ± 0.05) and average of the Pt(IV) standards (a/b = 2.4 ± 0.3) (values for standards in Table 5). From this, the % intracellular Pt(IV) for cells treated with **Pt2** under dark (a/b = 2.74 ± 0.05) and irradiated (a/b = 2.13 ± 0.04) conditions were calculated to be 116% and 85%, respectively (values in Table 6). Normalization to 100% Pt(IV) under dark conditions, gives 73% Pt(IV) under irradiated conditions.

## ES7 References

1. Farrer, N. J.; Woods, J. A.; Salassa, L.; Zhao, Y.; Robinson, K. S.; Clarkson, G.; Mackay, F. S.; Sadler, P. J., A Potent Trans-Diimine Platinum Anticancer Complex Photoactivated by Visible Light. *Angew. Chem. Int. Ed.* **2010,** *49* (47), 8905-8908.
2. Shi, H.; Imberti, C.; Clarkson, G. J.; Sadler, P. J., Axial functionalisation of photoactive diazido platinum(IV) anticancer complexes. *Inorg. Chem. Front.* **2020,** *7* (19), 3533-3540.
3. Phillips, M. A.; Harkiolaki, M.; Miguel, D.; Pinto, S.; Parton, R. M.; Palanca, A.; Garcia-Moreno, M.; Kounatidis, I.; Sedat, J. W.; Stuart, D. I.; Castello, A.; Booth, M. J.; Davis, I.; Dobbie, I. M., CryoSIM: super-resolution 3D structured illumination cryogenic fluorescence microscopy for correlated ultrastructural imaging. *Optica* **2020,** *7* (7), 802-812.
4. Schindelin, J.; Arganda-Carreras, I.; Frise, E.; Kaynig, V.; Longair, M.; Pietzsch, T.; Preibisch, S.; Rueden, C.; Saalfeld, S.; Schmid, B.; Tinevez, J. Y.; White, D. J.; Hartenstein, V.; Eliceiri, K.; Tomancak, P.; Cardona, A., Fiji: an open-source platform for biological-image analysis. *Nat. Methods* **2012,** *9* (7), 676-82.
5. Kremer, J. R.; Mastronarde, D. N.; McIntosh, J. R., Computer visualization of three-dimensional image data using IMOD. *J. Struct. Biol.* **1996,** *116* (1), 71-6.
6. Luengo, I.; Darrow, M. C.; Spink, M. C.; Sun, Y.; Dai, W.; He, C. Y.; Chiu, W.; Pridmore, T.; Ashton, A. W.; Duke, E. M. H.; Basham, M.; French, A. P., SuRVoS: Super-Region Volume Segmentation workbench. *J. Struct. Biol.* **2017,** *198* (1), 43-53.
7. Chow, K.-H.; Factor, R. E.; Ullman, K. S., The nuclear envelope environment and its cancer connections. *Nat. Rev. Cancer* **2012,** *12* (3), 196-209.

8. Shubin, A. V.; Demidyuk, I. V.; Komissarov, A. A.; Rafieva, L. M.; Kostrov, S. V., Cytoplasmic vacuolization in cell death and survival. *Oncotarget* **2016,** *7* (34), 55863-55889.
9. (2017) SOLUTION - Amira for Life Sciences. https://www.fei.com/software/amira-for-life-sciences/.
10. Carter, E. A.; Rayner, B. S.; McLeod, A. I.; Wu, L. E.; Marshall, C. P.; Levina, A.; Aitken, J. B.; Witting, P. K.; Lai, B.; Cai, Z.; Vogt, S.; Lee, Y.-C.; Chen, C.-I.; Tobin, M. J.; Harris, H. H.; Lay, P. A., Silicon nitride as a versatile growth substrate for microspectroscopic imaging and mapping of individual cells. *Mol. Biosyst.* **2010,** *6* (7), 1316-1322.
11. Solé, V. A.; Papillon, E.; Cotte, M.; Walter, P.; Susini, J., A multiplatform code for the analysis of energy-dispersive X-ray fluorescence spectra. *Spect. Chim. Acta B* **2007,** *62* (1), 63-68.
12. Rueden, C. T.; Schindelin, J.; Hiner, M. C.; DeZonia, B. E.; Walter, A. E.; Arena, E. T.; Eliceiri, K. W., ImageJ2: ImageJ for the next generation of scientific image data. *BMC Bioinform.* **2017,** *18* (1), 529.
13. Gregg, J. L.; McGuire, K. M.; Focht, D. C.; Model, M. A., Measurement of the thickness and volume of adherent cells using transmission-through-dye microscopy. *Pflugers Arch.* **2010,** *460* (6), 1097-104.
14. Lerotic, M.; Mak, R.; Wirick, S.; Meirer, F.; Jacobsen, C., MANTiS: a program for the analysis of X-ray spectromicroscopy data. *J Synchrotron Radiat* **2014,** *21* (Pt 5), 1206-12.
15. Ravel, B.; Newville, M., ATHENA, ARTEMIS, HEPHAESTUS: data analysis for X-ray absorption spectroscopy using IFEFFIT. *J. Synchrotron Radiat.* **2005,** *12* (Pt 4), 537-41.
16. Hall, M. D.; Daly, H. L.; Zhang, J. Z.; Zhang, M.; Alderden, R. A.; Pursche, D.; Foran, G. J.; Hambley, T. W., Quantitative measurement of the reduction of platinum(IV) complexes using X-ray absorption near-edge spectroscopy (XANES). *Metallomics* **2012,** *4* (6), 568-575.
17. Hall, M. D.; Foran, G. J.; Zhang, M.; Beale, P. J.; Hambley, T. W., XANES determination of the platinum oxidation state distribution in cancer cells treated with platinum(IV) anticancer agents. *J. Am. Chem. Soc.* **2003,** *125* (25), 7524-5.
